# Galactic Cosmic Rays Throughout the Heliosphere and in the Very Local Interstellar Medium

**DOI:** 10.1007/s11214-022-00912-4

**Published:** 2022-07-15

**Authors:** Jamie S. Rankin, Veronica Bindi, Andrei M. Bykov, Alan C. Cummings, Stefano Della Torre, Vladimir Florinski, Bernd Heber, Marius S. Potgieter, Edward C. Stone, Ming Zhang

**Affiliations:** 1 Department of Astrophysical Sciences, Princeton University, Princeton, NJ 08544 USA; 2 Physics and Astronomy Department, University of Hawaii, Honolulu, HI 96822 USA; 3 Ioffe Institute, 194021 St. Petersburg, Russia; 4 California Institute of Technology, Pasadena, CA 91125 USA; 5 INFN Milano-Bicocca, Piazza della Scienza 3, 20126 Milano, Italy; 6 Center for Space Plasma and Aeronomic Research (CSPAR) and Department of Space Science, University of Alabama in Huntsville, Huntsville, AL 35805 USA; 7 Institute for Experimental and Applied Physics, Christian Albrechts University in Kiel, Kiel, Germany; 8 Department of Physics and Space Sciences, Florida Institute of Technology, Melbourn, FL 32901 USA

## Abstract

We review recent observations and modeling developments on the subject of galactic cosmic rays through the heliosphere and in the Very Local Interstellar Medium, emphasizing knowledge that has accumulated over the past decade. We begin by highlighting key measurements of cosmic-ray spectra by Voyager, PAMELA, and AMS and discuss advances in global models of solar modulation. Next, we survey recent works related to large-scale, long-term spatial and temporal variations of cosmic rays in different regimes of the solar wind. Then we highlight new discoveries from beyond the heliopause and link these to the short-term evolution of transients caused by solar activity. Lastly, we visit new results that yield interesting insights from a broader astrophysical perspective.

## Introduction

The past decade of cosmic ray history was characterized by reaching several important milestones. The Voyager probes crossed beyond the external boundary of the heliosphere and into a new plasma region commonly referred to as the “Very Local Interstellar Medium”, thereby enabling the first observations of low-energy GCRs (few to hundreds of MeV/nuc) before they undergo significant modulation inside the heliosphere. Meanwhile, the Payload for Antimatter Matter Exploration and Light-nuclei Astrophysics (PAMELA) (Pamela Collaboration 2017) mission completed ten years of observations, while the Alpha Magnetic Spectrometer (AMS–02) (Aguilar 2021b) began its long-duration mission. These data have initiated a new era in cosmic rays observations at 1 AU (Earth), both in exploring the very high rigidity (1 to 3 TV), and providing highly-accurate details of how GCR spectra evolve within the heliosphere over time (i.e. with solar activity). This, amongst many things, represents significant advancement for the monitoring of space radiation (see, e.g., Aguilar 2021a) and has also led to a better understanding of how charge-sign dependent behaviour varies during different phases of solar activity (see also Aslam et al. 2021). Together, the above three missions have produced a significant amount of new data that not only provides strong constraints on galactic propagation models, but also allows the scientific community to explore phenomena that were only previously inferred. The results have thus, collectively, reinforced some paradigms – such as that of solar modulation (Potgieter 2017) – and have also led to entirely unanticipated discoveries, providing ample hints at the potential for new insights in both physical (see, e.g., Cuoco et al. 2018; Cui et al. 2018; Cholis et al. 2019) and astrophysical scenarios (see, e.g., Boschini et al. 2020c, 2021).

In this chapter we review the state-of-art of measurements and models of galactic cosmic rays (GCRS) throughout the heliosphere and in the Very Local Interstellar Medium (VLISM). In Sects. 2, 3 and 4 we present key measurements of the GCR spectra performed by the Voyagers, PAMELA and AMS–02. We begin by detailing Voyagers’ first measurements of the pristine local interstellar spectra (Sect. 2). Then we highlight key spectral results at 1 AU from PAMELA and AMS–02 (Sects. 3–4). Next, we review how these collective data sets have led to a more complete understanding of solar modulation, first by describing advancements in global models (Sect. 5), and then by considering how these descriptions relate to observations of temporal and spatial variations of throughout the heliosphere (Sect. 6). Afterward, we survey the limits of solar modulation, including in the heliosheath where levels are strongest, and at the heliopause boundary – beyond which, the effects are insignificant (Sect. 7). From here, we present on the discovery of a time-dependent, pitch-angle-dependent, and species-dependent anisotropy (Sect. 8). We relate these findings to solar-induced transient disturbances which progress as distinct events in the inner heliosphere and coalesce into large-scale structures which propagate through the heliosheath (Sect. 9) eventually exert their influence on the surrounding VLISM plasma (Sect. 10). Lastly, we provide a more astrophysical perspective by exploring observations of GCRs on broader scales, highlighting examples such as anisotropies at the TeV scale and the contribution of nearby sources to GeV-TeV leptons (Sect. 11).

## The Very Local Interstellar Spectra

Due to the effects of solar modulation and the presence of anomalous cosmic rays in the heliosphere, the energy spectra of GCR nuclei in the VLISM were essentially unknown at energies below a few hundred MeV/nuc prior to the crossing of the heliopause by Voyager 1 in 2012. For example, Wiedenbeck (2013) showed that the interstellar spectra of protons could vary by factors of $>100$ below $\sim100~\text{MeV}$ and yet the energy spectrum at 1 AU could be the same to within 1%. Further, due to adiabatic energy losses (e.g. Strauss et al. 2011) incurred during their transport from the VLISM, the energies of the GCR nuclei observed at 1 AU are reduced from their energies in the VLISM by typically hundreds of MeV/nuc. Estimates of the GCR electron spectra in the VLISM also varied, by up to a factor of 10 at 10 MeV (see Cummings et al. 2016). The Voyager 1 (V1) and Voyager 2 (V2) observations in the VLISM have now provided these low-energy spectra down to energies as low as 3 MeV/nuc for elements with nuclear charge $Z = 1$ to 28 and down to 2.7 MeV for the total electron ($e^{+} + e^{-}$) component of GCRs (Stone et al. 2013; Cummings et al. 2016; Stone et al. 2019).

### In-Situ Measurements

**Fig. 1:**
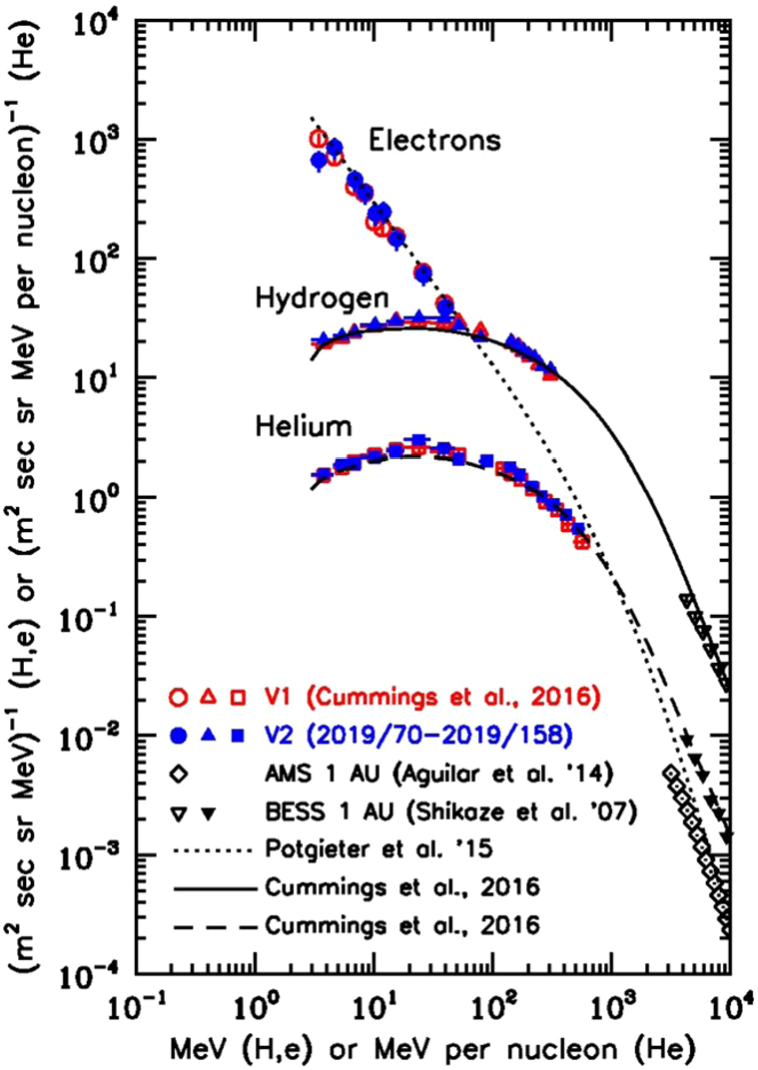
Reproduced from Cummings et al. (2016). Energy spectra of H, He, and total electrons ($e^{+} + e^{-}$) are shown for V1 and V2 in the VLISM over the time periods of 2012/342–2015/181 (V1; red) and 2019/70–2019/158 (V2; blue). Also shown are high-energy portions of observed spectra at 1 AU that are expected to be only slightly affected by solar modulation effects. The lines represent theoretical estimates of interstellar spectra

Figure 1, from Stone et al. (2019), shows that the energy spectra of GCR H, He, and total electrons are essentially the same at V1 and V2, respectively, despite a spatial separation of 167 AU between the two spacecraft at the time V2 crossed the heliopause. Cummings et al. (2016) also showed that the radial gradient of GCR protons from 3 to 346 MeV was consistent with zero over a distance of 9.2 AU into the VLISM.

Figure 1 also shows that the GCR H and He spectra in the VLISM have broad intensity maxima in the energy range of 10 to 50 MeV/nuc. The spectral shape is similar for H and He in the units shown and the H/He ratio is $12.2\pm0.09$ (Cummings et al. 2016). The maximum H intensity is $\sim15$ times higher than the maximum intensity observed at 1 AU during solar minimum conditions (Cummings et al. 2016). It is interesting to note that the paradigm of GCR electron intensities being 1% of protons only holds at high energies and that the GCR electron intensity exceeds that of protons below $\sim50~\text{MeV}$. The electron spectrum exhibits a power-law with index of −1.3 over the energy range of observations, (2.7 to 74.1 MeV) whereas the protons and helium spectra have flattened and are even decreasing in intensity at low energies. As a result, the GCR electron intensity at 3 MeV is a factor of $\sim50$ higher than that of GCR protons.

These VLISM energy spectra have important implications for astrophysics, some of which were explored in Cummings et al. (2016). For example, it was estimated therein that the energy density of GCRs in the VLISM is in the range of 0.83 to $1.02~\text{eV/cm}^{3}$ and the ionization rate of atomic H is in the range $1.51 \times 10^{-17}$ to $1.64\times 10^{-17}~\text{s}^{-1}$. This ionization rate is a factor of 11 to 12 lower than that inferred from astro-chemistry techniques for diffuse molecular clouds (Indriolo et al. 2015), suggesting that the GCR spectra are likely variable across the galaxy.

The determination of the Local Interstellar Spectra (LIS) is an excellent example of how Earth-orbit spectrometers and interplanetary probes may provide complementary information. Below few tens of GeV, the intensity of GCRs at Earth decreases with respect to the GCR energy spectrum outside the heliosphere. This effect is due to the interaction of GCRs with the expanding solar wind and its embedded turbulent magnetic field, as well as transport effects such as convection, diffusion, adiabatic energy losses, and particle drifts arising from the global curvature and gradients of the Heliospheric Magnetic Field (HMF) (see, e.g. Potgieter 2013a; Boschini et al. 2019). In previous decades, Earth-orbit observations could only exploit the LIS at high energy where solar modulation effects were considered negligible (see, e.g. Strauss and Potgieter 2014b), whereas the low-energy part of the LIS could only be inferred from Galactic propagation models (Cummings et al. 2016; Stone et al. 2019; Webber 2016; Webber et al. 2013). However, since the two Voyager probes have ventured beyond the heliopause, this situation has improved significantly.

For example, by combining Voyager 1 data with AMS–02, PAMELA, and earlier BESS-Polar measurements, the work of Cholis et al. (2016), Corti et al. (2016), and Ghelfi et al. (2016) derived the LIS for protons and He and then used the force-field approximation (Gleeson and Axford 1968) to aim for a generalization of the modulation potential dependent upon time, charge sign, and rigidity.

In general, the use of numerical modulation codes to derive physically-motivated LIS has become more comprehensive with time. For example, several authors have derived the LIS for electrons and positrons using 3D numerical modulation models (Potgieter and Nndanganeni 2013; Potgieter et al. 2015; Aslam et al. 2021). More comprehensive approaches have also enabled the derivation of the LIS for protons, Helium, Oxygen, and Carbon, as well as He-3 and He-4 isotopes and the averaged ratio of Boron to Carbon (observed by PAMELA) (e.g., Bisschoff and Potgieter 2014, 2016; Bisschoff et al. 2019; Ngobeni et al. 2020). These latter models used Voyager 1 and PAMELA data together with GALPROP calculations for interstellar propagation.

**Fig. 2:**
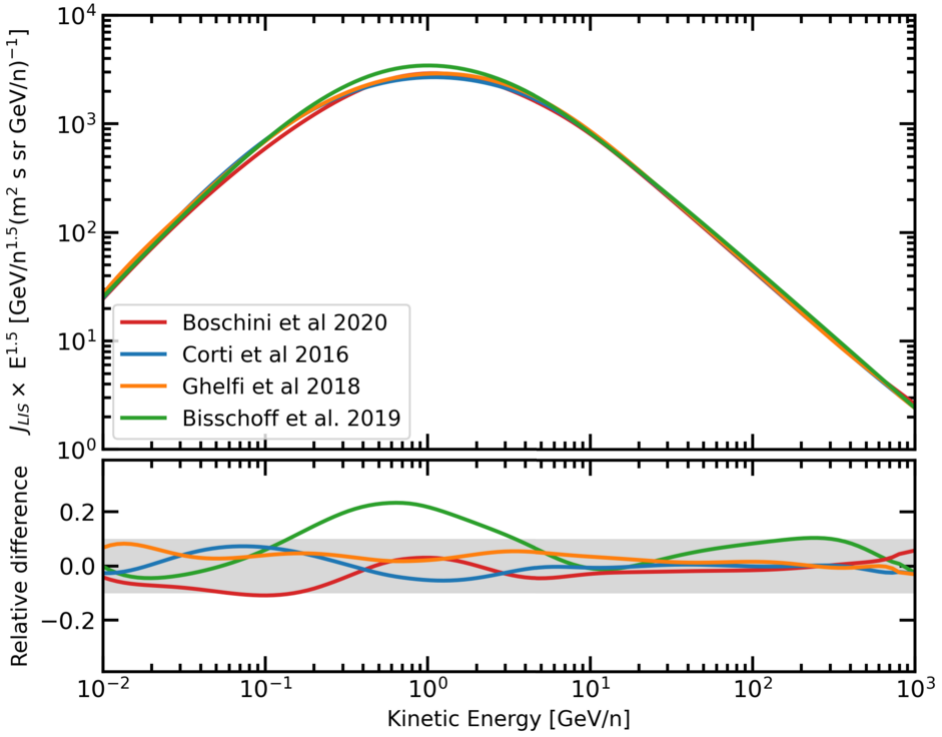
Top panel: Proton LIS differential intensity ($J_{LIS}$) obtained from Bisschoff and Potgieter (2016) (green line), Boschini et al. (2020b) (red line), Corti et al. (2016) (blue line) and (Ghelfi et al. 2016, orange line) (orange line). Bottom panel: LIS relative difference for an average intensity between the last three results; the grey band highlight an arbitrary 10% agreement band

Boschini et al. (2017, 2018a,b, 2020a,b, 2021, 2022) inferred LIS for GCRs $e^{-}$, $\bar{p}$ and ions with $Z < 28$ by combining Voyager, AMS–02 and HEAO3-C2 (Engelmann et al. 1990) data within the so-called GALPROP-HelMod framework (Boschini et al. 2017) that derived LIS through an iterative procedure that cross-tune the free galactic and heliospheric propagation parameters in the numerical models. For protons, the comparison among these LIS expressions is reported in Fig. 2. As shown here, the expressions agree well, within 10% of each other at both low and high energies. However, in the intermediate energy range, the LIS could only be inferred using galactic propagation models, contributing to a spread of global uncertainty. See Bisschoff et al. (2021) for an updated list of the LIS for several GCRs (and their anti-particles) relevant to solar modulation studies.

## Solar Modulation and New Evidence of Charge-Sign Dependence by PAMELA

The PAMELA cosmic ray detector (Picozza et al. 2007) operated onboard the Russian Resurs-DK1 satellite from 2006 to 2016. Its continuous and high-precision measurement of several cosmic ray species – including charged anti-matter particles – contributed significantly to the understanding of solar modulation from the prolonged solar minimum before 2010 until after solar maximum modulation of solar cycle 24, including the reversal on the HMF ‘polarity’ in 2013–2014 (see the review by Boezio et al. 2017, and references therein). Figure 10 of Adriani et al. (2017) shows a full set of GCR spectra observed by PAMELA, along with solar energetic particles and particles trapped in the Earth’s magnetosphere. PAMELA also measured the time-dependent solar modulation of GCR protons from 0.4 GV to 30 GV at Earth, shown by Boezio et al. (2017) from July 2006 to May 2014 (see their Fig. 7). Proton fluxes during the minimum to maximum conditions of solar activity through solar cycle 24 were specifically described by Martucci et al. (2018), and Marcelli et al. (2020, 2022) reported on the time dependent modulation of Helium nuclei between July 2006 and December 2009, and from January 2010 to September 2014, respectively.

**Fig. 3:**
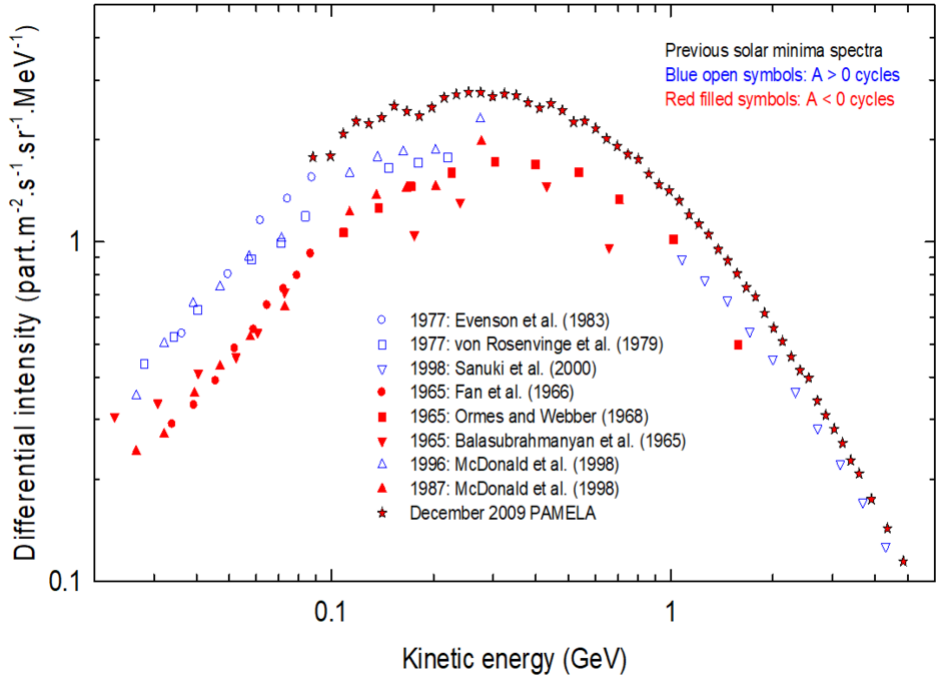
Proton spectra observed during five solar minimum modulation periods. $\text{A}>0$ spectra are shown as blue symbols and those for $\text{A}<0$ in red. The PAMELA proton spectrum for the end of 2009 is indicated by stars. References to the data sets were given by Strauss and Potgieter (2014a)

At the end of 2009, PAMELA reported the highest flux of Galactic Cosmic Rays (GCRs) ever recorded (also seen by NASA’s Advanced Composition Explorer, ACE; see, e.g. Mewaldt et al. 2010; Leske et al. 2013). According to drift model predictions of the 22-year cycle in the solar modulation of GCRs, it was expected that the 2009 proton spectrum would agree with those of previous $\text{A}<0$ cycles, but instead it was substantially higher and softer than any other previous spectra, as shown in Fig. 3. The reason for this was discussed in detail by Potgieter et al. (2014) and Strauss and Potgieter (2014a), who concluded that drifts were indeed present, but the global modulation in 2009 was diffusion dominated, thereby causing drift effects – although not the drift velocities – to be subdued by diffusion. According to this argument, the proton spectra for the present solar minimum modulation (2020–2021) could be even higher if modulation conditions are similar to those during 2006 to 2009, because spectra during an $\text{A}>0$ cycle are expected to give higher fluxes at kinetic energies below about 500 MeV (see also predictions by Potgieter and Vos 2017; Krainev et al. 2021).

**Fig. 4:**
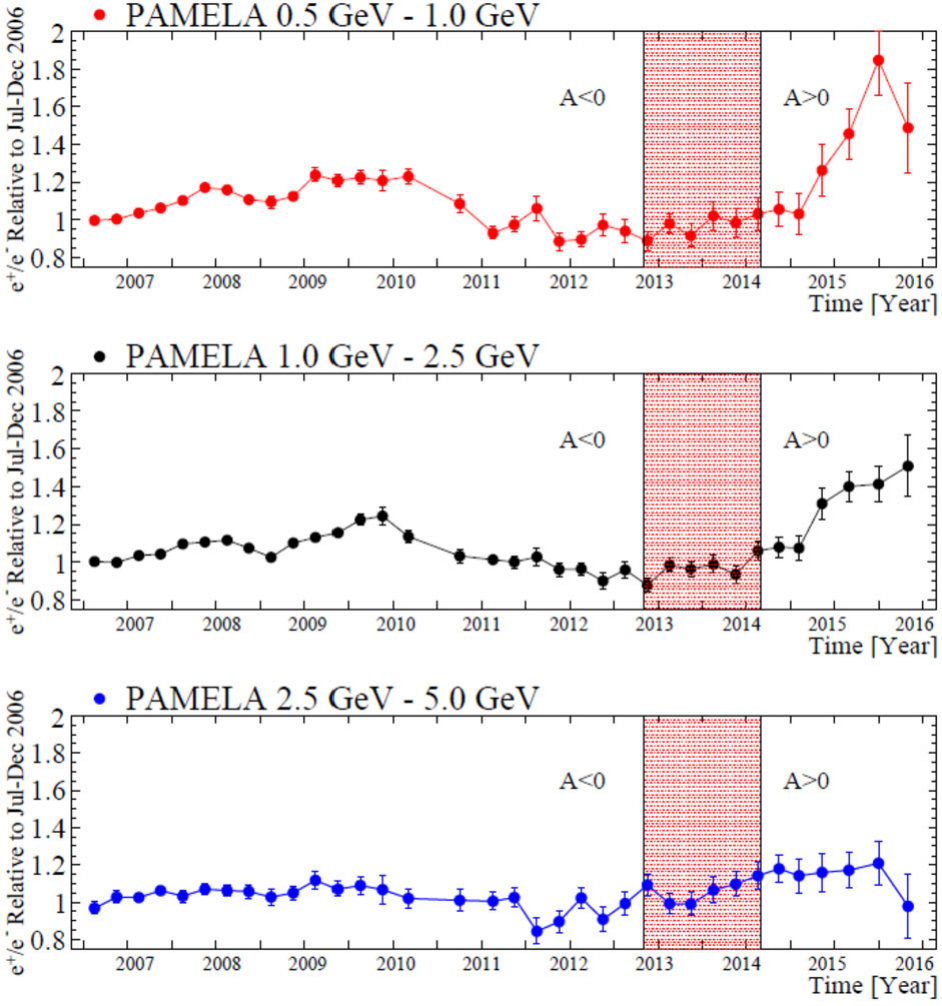
Charge-sign dependence shown by three energy intervals of the positron to electron ratio measured by PAMELA at Earth for three energy intervals between 0.5 GeV and 5.0 GeV over the time period of July 2006 to December 2015, normalized to 2006. The shaded area indicates the period with no well-defined HMF polarity (from Fig. 1 of Adriani et al. 2016)

A most exciting observation from PAMELA was that of the time-dependence of charge-sign modulation in terms of electrons and positrons, as shown in Fig. 4. The figure illustrates how the positron to electron ratio had changed from July 2006 to December 2015 with respect to 2006 and what happened when the HMF ‘polarity’ had changed from the $\text{A}<0$ cycle before 2013 to the $\text{A}>0$ cycle after 2014. Evidently, the ratio changed by about a factor of 2 for 0.5 to 1.0 GeV particles but much less in the 2.5 to 5.0 GeV range. PAMELA also measured a well-defined charge-sign dependent effect during the prolonged solar minimum period from 2006 to 2009, evidenced by the difference in how proton and electron intensities evolved with decreasing solar activity during this period. The corresponding electron to proton ratio in comparison with modeling results was shown by Di Felice et al. (2017) and Adriani et al. (2017).

## A Solar Cycle of Measurements from AMS

The Alpha Magnetic Spectrometer (AMS) is a state-of-the-art particle detector that measures charged particles from the GeV to TeV energy range. It was installed on the International Space Station (ISS) in May 2011, where it will operate for the duration of the station, until 2028. AMS began taking data during the ascending phase to solar maximum during SC 24. AMS has performed continuous measurements of GCR fluxes for nearly a full solar cycle, and after 10 years of operation, has collected more than 176 billion events – including protons, electrons, positrons, nuclei and light isotopes. AMS has five sub-detectors that enable redundant measurements of particle charge, velocity, and energy. The instrument’s large acceptance is key to its ability to collect the high statistics necessary for studying rare species and performing precise measurements of the time evolution of GCRs. The mission’s long duration will allow for precise time-dependent measurements of GCRs during multiple phases of solar activity, and will ultimately lead to a better understanding of the propagation of charged particles through the solar wind and its embedded magnetic field.

**Fig. 5:**
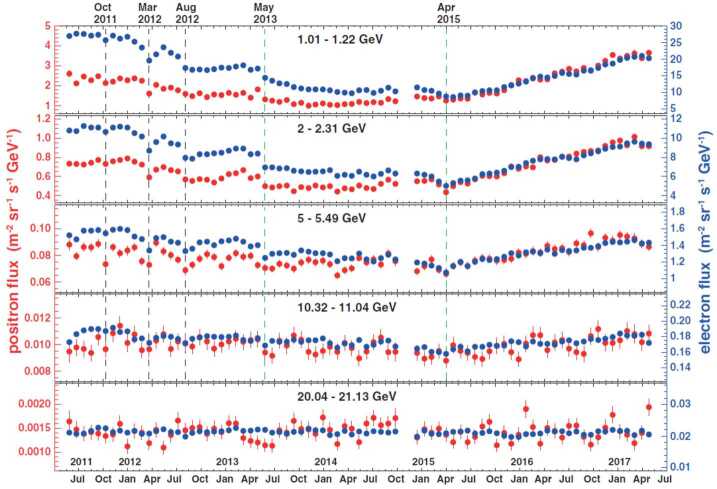
Fluxes of cosmic-ray positrons (red, left axis) and electrons (blue, right axis) as functions of time, for five energy bins, measured by AMS. The error bars represent statistical uncertainties. Prominent and distinct time structures visible in both the positron spectrum and the electron spectrum and at different energies are marked by dashed vertical lines (from Aguilar 2018b)

**Fig. 6:**
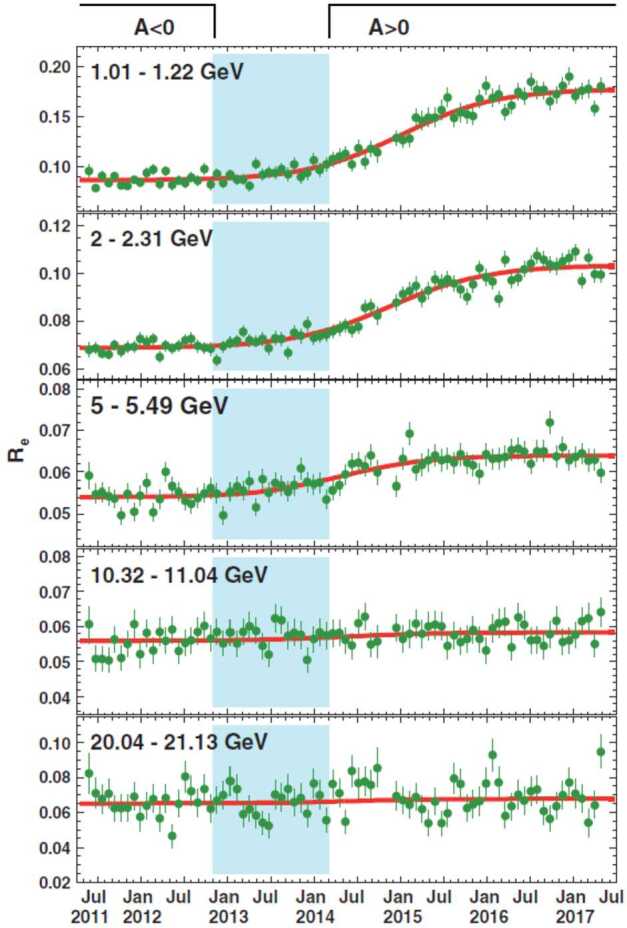
The ratio of the positron flux to the electron flux as a function of time measured by PAMELA from May 2011 to May 2017, with error bars indicating statistical uncertainties. The best-fit parametrization of a logistic function is shown by the red curves. The polarity of the HMF is denoted by $\text{A}<0$ and $\text{A}>0$, while the shaded area marks the period when the polarity is not well-defined (figure from Aguilar 2018b)

Time-dependent structures in the GCR energy spectra are expected from the solar modulation. Of the convective, diffusive, particle drift, and adiabatic energy loss mechanisms responsible for solar modulation, only particle drift is dependent on the sign of the charge. Since the only difference between electrons and positrons is reflected in the latter, their simultaneous measurement offers a unique way to study solar modulation effects that are strictly charge-sign dependent. From May 2011 to May 2017, AMS accumulated precise, high-statistic measurements of the time variation of electron and positron fluxes from 1 to 50 GeV (Aguilar 2018b). The data over these 79 Bartels rotations (BR, 27 days) exhibited profound short- and long-term variations, as shown in Fig. 5. The short-term variations occurred simultaneously with approximately the same relative amplitude for both electrons and the positrons, and the effect of solar modulation gradually diminished with increasing energies. At energies above 20 GeV, neither the electron flux nor the positron flux exhibited significant time dependence. The short time structures are not visible in the positron to electron flux ratio ($e^{+}$/$e^{-}$), as evidenced by Fig. 6. Instead, a long-term behavior is observed, characterized by a smooth transition that occurs after the polarity reversal of the solar magnetic field. The transition lasts $830\pm30$ days, and although its duration is independent of energy, its magnitude decreases as a function of energy. The midpoint of the transition relative to the polarity reversal of the solar magnetic field changes by $260\pm30$ days from 1 to 6 GeV.

Since the transport of cosmic rays within the heliosphere is rigidity dependent, it is generally expected that particles with the same rigidity should show the same behavior over time. However, some Parker-transport based models have shown that particles with the same rigidity might exhibit a different time behavior due to differences in their velocities (i.e. different mass-to-charge-ratio) and different rigidity dependence of their LIS (Corti et al. 2019a). These findings are supported by recent measurements from AMS of proton, Helium, Carbon and Oxygen fluxes.

**Fig. 7:**
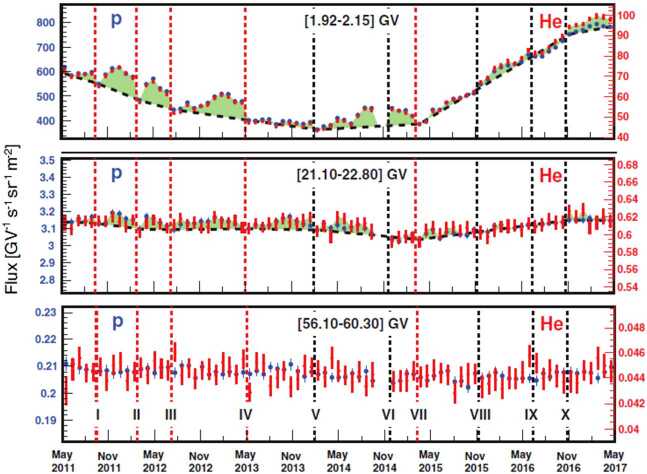
The AMS proton (blue, left axis) and helium (red, right axis) fluxes as function of time for 3 rigidity bins. Detailed structures (green shading and dashed lines) are clearly present below 40 GV. The vertical dashed lines denote boundaries between these structures at I) September 27, 2011; II) March 7, 2012; III) July 20, 2012; IV) May 13, 2013; V) February 7, 2014; VI) December 1, 2014; VII) March 19, 2015; VIII) November 17, 2015; IX) June 20, 2016; X) November 28, 2016. The red vertical dashed lines denote structures that have also been observed by AMS in the electron and positron fluxes. The error bars represent the quadratic sum of the statistical and time dependent systematic errors (figure from Aguilar 2018a)

AMS–02 performed precise measurements of proton and Helium fluxes over the 79 BRs from May 2011 to May 2017, in the 1 to 60 GV rigidity range (Aguilar 2018a). Figure 7 shows the measured time profiles of these fluxes at different rigidity bins. Fine structures related to solar modulation are present for both species and their variations are nearly identical in both time and relative amplitude. However, the structures are observed in protons up to $\sim40~\text{GV}$ and Helium up to $\sim20~\text{GV}$, and their amplitudes progressively decrease with increasing rigidity.

**Fig. 8:**
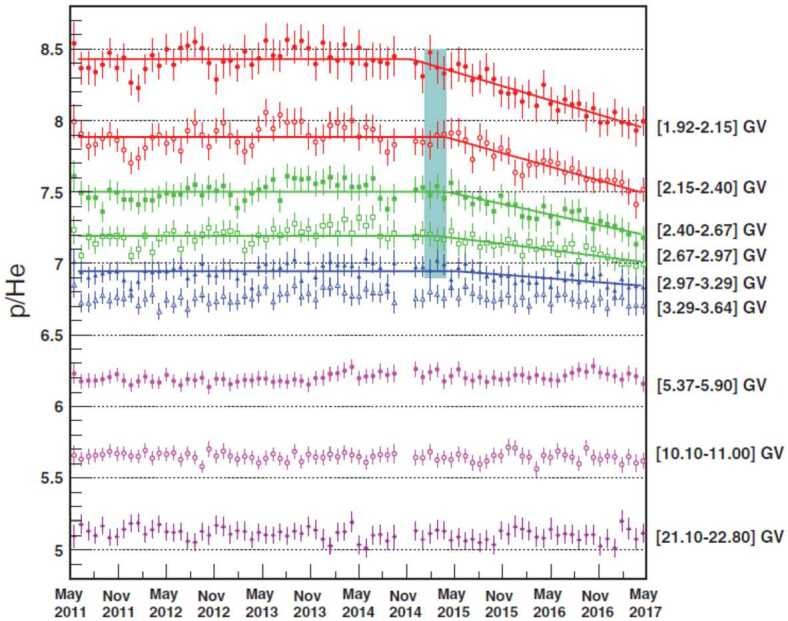
The AMS p/He flux ratio as function of time for 9 characteristic rigidity bins. The errors are the quadratic sum of the statistical and time dependent systematic errors. The solid lines are the best fit for the first 5 rigidity bins from [1.92–2.15] GV to [2.97–3.29] GV. The blue vertical band (February 28, $2015\pm42$ days) is the average of the best fit values of transition time for these rigidity bins (figure from Aguilar 2018a)

The p/He flux ratio measured by AMS is shown in Fig. 8. For rigidities greater than 3 GV, when both species reach relativistic energies, the p/He ratio is independent of time, indicating that the effects of modulation are the same for cosmic ray protons and Helium at relativistic energies. On the other hand, below $\sim3~\text{GV}$, the observed p/He flux ratio is steadily decreasing with time starting with the start of the flux recovery period after the solar maximum. This long term variation may be due to both differences in the diffusion coefficient due to different velocity dependence, and the different shapes of the LIS versus rigidity. Since protons and Helium nuclei have a different mass-to-charge ratio it is not possible to disentangle the contribution of the LIS and of the velocity dependence of the diffusion coefficient, but numerical models are needed. Recent work by Corti et al. (2019a) suggested that this p/He variation is related to velocity differences of the two species.

In principle, particles with the same mass to charge ratios are expected to have the same diffusion coefficients for a given rigidity; therefore differences in the time behaviour can be related to differences in their LIS (Corti et al. 2019b). As such, the simultaneous measurement of different particles with similar mass-to-charge ratio, such as Helium, Carbon and O, provides unique information on their LIS. The time evolution of Carbon and Oxygen fluxes in the rigidity range [$2, 60$] GV was measured by AMS from May 2011 to October 2019 and presented at COSPAR 2020 (Donnini 2021). This represents the first and unique measurement of the time dependence of Carbon and Oxygen fluxes as a function of rigidity. The time profile of Carbon and Oxygen fluxes shows identical short- and long-term structures. As for other species, the amplitude of the structure decreases with increasing rigidity and becomes non-observable above $\sim25~\text{GV}$. The C/O flux ratio was observed to be time independent in the whole rigidity range. Since Carbon and Oxygen have the same mass-to-charge ratio, it is possible to conclude that the rigidity dependence of their LIS is very similar above 2 GV. The same conclusion can be drawn from the flux measurements performed by Voyager below 1 GV (Cummings et al. 2016).

## Advances in Global Models of Solar Modulation

In a review of the global modulation of GCRs during the quiet solar activity period of 2006 to 2009, Potgieter (2017) emphasized the point that determining and understanding of the total, global modulation in the heliosphere had always been one of the primary objectives of observational, theoretical and numerical studies. In this context, the observation of the position of the termination shock (TS), and later the position of the heliopause (HP) in the nose direction of the heliosphere and the corresponding VLIS’s for several GCR particle species at low kinetic energies, have been major steps forward. Together with PAMELA and AMS–02 observations at very high kinetic energies, the VLIS’s for several GCR species could be determined far better than before, as described above. Another objective was to gain insight into the physical processes responsible for the solar modulation of GCRs such as the relative roles of the processes described by Parker’s transport equation (Parker 1965). This has been done through comprehensive and global numerical modeling.

As explained above, for GCRs the solar minimum modulation period from 2006 to the end of 2009 was quite unusual. This was characterized by a much weaker HMF compared to previous cycles and record setting GCR intensities (see also Giacalone et al. [2022] chapter for details regarding anomalous cosmic rays).

**Fig. 9:**
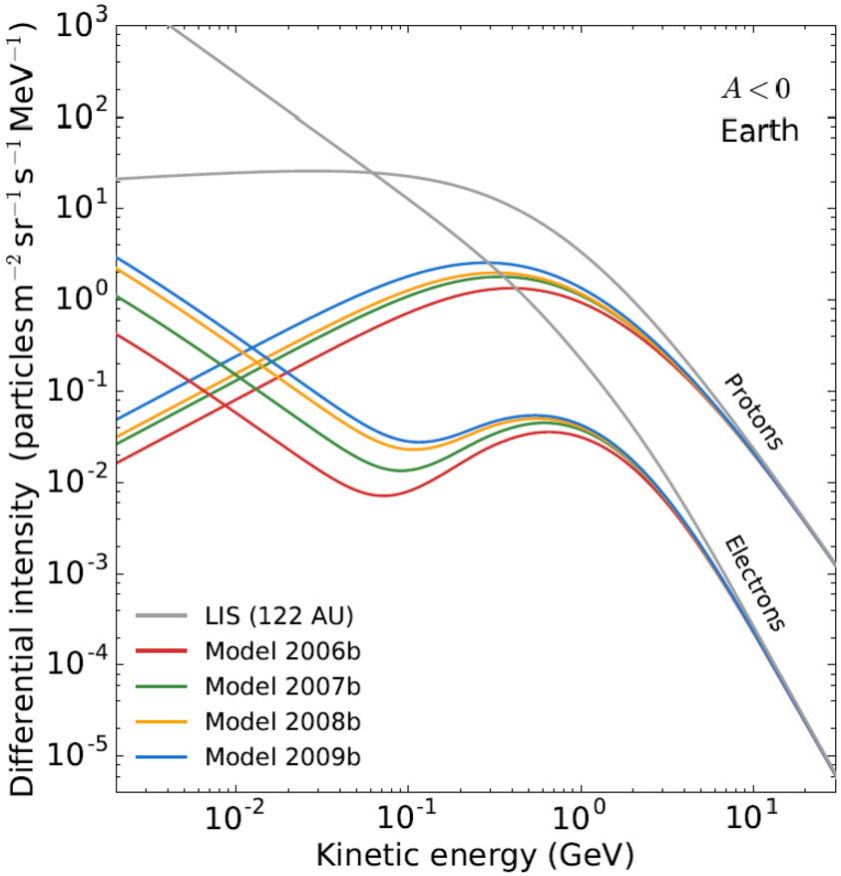
Differences between computed electron and proton spectra at Earth are shown for 2006 (lowest spectra) and 2007, 2008 and 2009 (highest spectra), based on the PAMELA observations during this period. Below 100 MeV, where there are no corresponding observations, these computed spectra are predictions of what could have been observed during this $\text{A}<0$ polarity cycle solar minimum (from Fig. 4 of Potgieter and Vos 2017)

The proton, electron, and Helium spectra observed down to 80 MeV/nuc by PAMELA (Adriani et al. 2017) have been extensively reproduced through comprehensive simulations, with explanations given by Potgieter et al. (2014), Potgieter and Vos (2017) and Ngobeni et al. (2020). These efforts were reviewed in detail by Potgieter (2017). Figure 9 is an example of the modelling done for GCR protons and electrons for 2006 to 2009, during an $\text{A}<0$ polarity cycle, illustrating the vast differences between the modulation of these particles with respect to their VLIS’s at 122 AU. It should be noted that, for electrons, the spectra below about 50 MeV would change significantly if Jovian electrons were included in this simulation. For such computed spectra, see Nndanganeni and Potgieter (2018); for recent observations of these low energy electrons, see Vogt et al. (2018) and Mechbal et al. (2020).

**Fig. 10:**
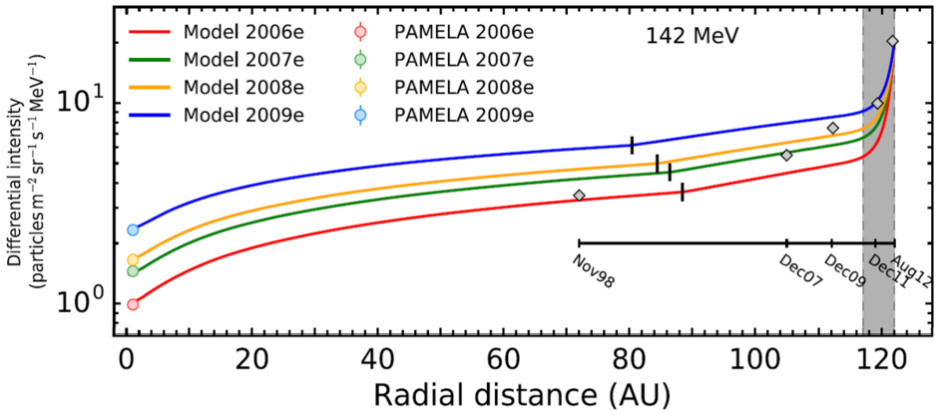
Computed radial intensities for 182 MeV protons are shown from the Earth for 2006 (red line) and 2009 (blue line) up to the HP fixed at 122 AU, while the TS position is shifted with time as indicated by the short vertical black lines. Four profiles are compared to Voyager 1 measurements beyond 100 AU (Webber et al. 2013). Shaded part beyond 116 AU is the HP region where significant additional modulation occurs. Figure from Vos and Potgieter (2016)

Corti et al. (2019b) addressed with numerical modeling the proton to Helium ratio observed by AMS–02 during the solar maximum of solar cycle 24, with similar studies done by Tomassetti (2017) and Tomassetti et al. (2019). Ngobeni et al. (2020) focused specifically on the difference between GCR protons and Helium, emphasizing the contribution to the total modulation of Helium (He) by the two isotopes He-3 and He-4. They computed the proton to total He ratio for 2006 to 2009 and found that modulated spectra do not undergo identical spectral changes below about 3 GV mainly due to differences in their VLIS’s and further illustrated what kind of differences could be expected caused by the difference in their VLIS’s and in their different A/Z ratio. Vos and Potgieter (2016) did a comprehensive study of the global radial dependence of GCR protons for this period. This is shown in Fig. 10. They also presented computed radial and latitudinal gradients for the inner heliosphere based on PAMELA and Ulysses observations for the solar minimum of cycle 23/24 (see their Fig. 9).

**Fig. 11:**
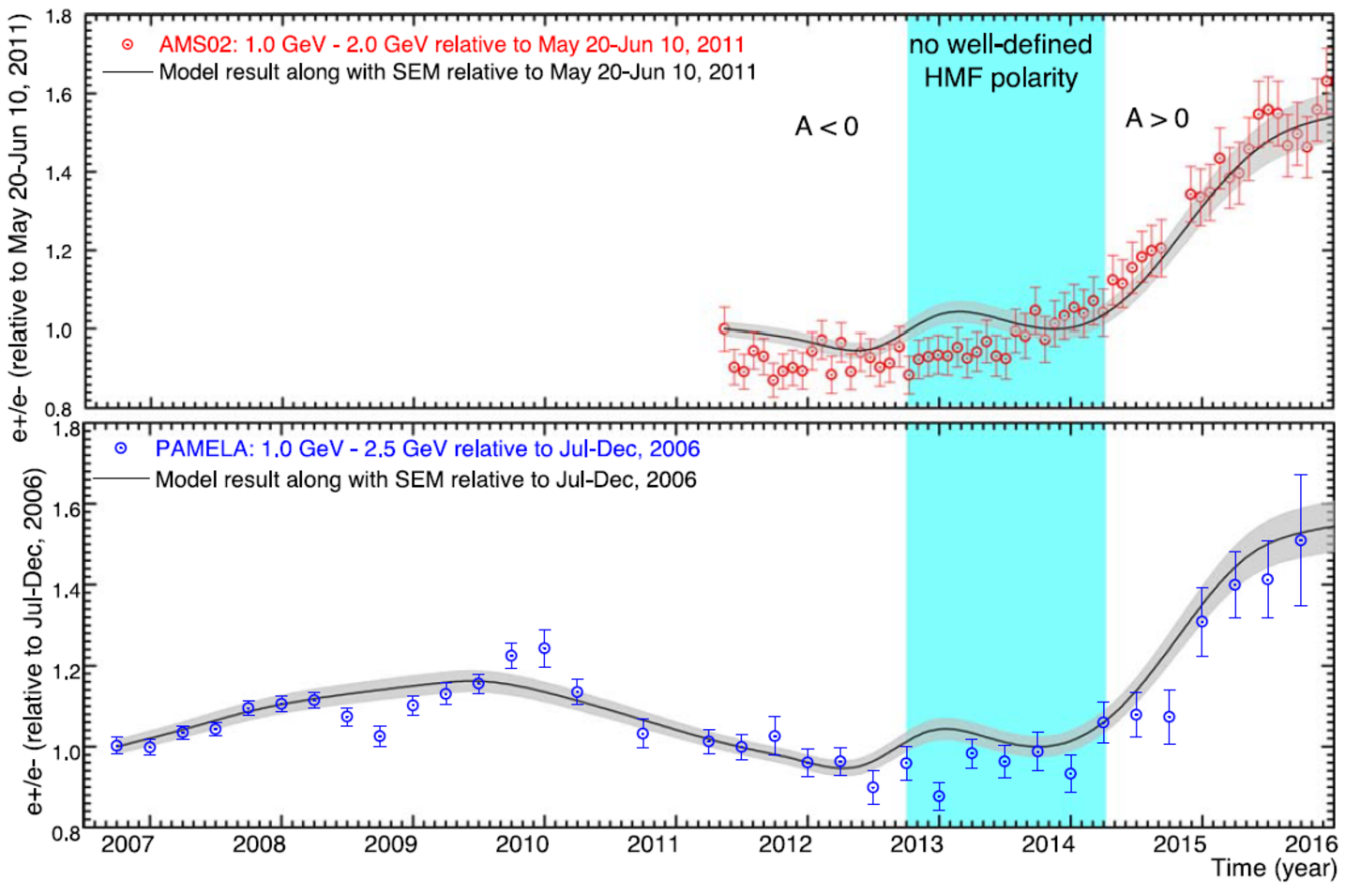
Top panel: Computed $e^{+}$/$e^{-}$ (solid line) is shown in comparison with AMS–02 observations for 1.0–2.0 GeV (red dots; Aguilar (2018a)), averaged over Bartels rotations for May 2011 to December 2015 and normalized with respect to May 2011. Bottom panel: Computed $e^{+}$/$e^{-}$ is shown in comparison with the observed ratio by PAMELA for 1.0–2.5 GeV (blue dots; Adriani et al. 2016), averaged over 3 months from July 2006 to December 2015. Both ratios are normalized to July–December 2006. Shaded regions indicate the period without a well-defined HMF polarity. From Fig. 4 of Aslam et al. (2021)

The simultaneous observations of GCR electrons and positrons from PAMELA and AMS are most suitable for the numerical modeling of the modulation of these particles below 50 GeV. Aslam et al. (2019, 2021) presented a numerical modelling study of GCR positrons and electrons done with a 3D drift modulation model for the period of 2006 to 2015. They compared their simulations of the positron to electron ratio ($e^{+}$/$e^{-}$) with PAMELA and AMS–02 observations up to 2016, including the HMF reversal period in 2013–2014. Their study was focused on how the main modulation processes, including particle drifts, had evolved over these years and how the corresponding charge sign-dependent modulation subsequently had occurred, specifically how much particle drift was needed to explain the time dependence exhibited by the observed ratio, especially during the polarity reversal phase when no well-defined magnetic polarity was found (Sun et al. 2015). Their simulations displayed both qualitative and quantitative agreement with the main observed features, which is also qualitatively similar to Ulysses observations (Heber and Potgieter 2006). The comparison of their computed electron to positron ratio with observations is shown in Fig. 11. There is clearly no large ‘jump’ in the intensities of GCRs during the reversal period as computed by Tomassetti et al. (2017). The required changes to the rigidity and time-dependence of the diffusion coefficients and the drift coefficient to obtain these ratios were also shown and discussed by Aslam et al. (2021); see their Figs. 5 to 8. Concerning modelling of the 22-year cycle, Potgieter and Vos (2017) used their comprehensive 3D modulation model to illustrate how electrons and protons are differently modulated down to 1 MeV, based on new VLIS’s and observations of these GCRs spectra by PAMELA as mentioned above. They computed spectra for protons and electrons for the two HMF polarity cycles and showed that a cross-over of $\text{A}>0$ and $\text{A}<0$ spectra could occur and made predictions of what may be observed during the present $\text{A}>0$ solar minimum period (2020 to 2022) if similar conditions would prevail as in 2006 to 2009. These spectral cross-overs are required to explain why proton spectra at lower KE, less than about 500 MeV, is usually lower in $\text{A}<0$ cycles than in $\text{A}>0$ cycles (except for 2009; see Fig. 3) but at higher KE, above about 5 GeV, the intensity is usually higher during $\text{A}<0$ cycles than in $\text{A}>0$ cycles; see reviews by Potgieter (2013b,a) and recent work on these spectral cross-overs by Krainev et al. (2021) and references therein.

## Temporal and Spatial Variations of Galactic Cosmic Rays Throughout the Heliosphere

As discussed in Sect. 5 above, GCR modulation is caused by a number of physical processes, including spatial diffusion in the turbulent heliospheric magnetic field, convection and adiabatic deceleration in the expanding solar wind, gradient and curvature drift in the large scale magnetic fields. Jokipii et al. (1977) pointed out that gradient and curvature drifts in the large-scale HMF, approximated by a three-dimensional Archimedean spiral (Parker 1958), should also be an important element of GCR modulation. The strength and relative importance of these processes varies with the location in the heliosphere and with the 22-year solar magnetic cycle. While continuous measurements of GCRs goes back to the invention of the Neutron Monitor (NM), measurements resolving the energy spectra and chemical composition became possible with instrumentation on balloons and spacecraft. Electron (negatrons including positrons) observations go back to the 1960’s on balloons (Webber et al. 1973; Freir and Waddington 1965) and on spacecraft to the 1970’s with the launch of the Orbiting Geophysical Observatories (OGO)-5 and International Sun Earth Explorer (ISEE)-3/International Cometary Explorer (ICE) close to Earth (Burger and Swanenburg 1973; Garcia-Munoz et al. 1986; Clem et al. 1996). Until the 1990’s there had been no mission exploring GCR electron fluxes beyond the Earth orbit due to the limitation of the instrumentation and Jupiter’s dominance as a source of electrons in the intermediate heliosphere out to at least 20 AU. (Ferreira et al. 2004; Strauss et al. 2013a). However, Nndanganeni and Potgieter (2018), in an updated modelling of jovian electrons, showed that the contribution of GCR electrons below 100 MeV becomes increasingly dominant with radial distances beyond 30 AU.

In terms of measurements, the past decade has also been distinctly characterized by multi-spacecraft observations. Spatial gradients (Vos and Potgieter 2016), both in heliospheric latitude and solar radial distance, were observed by early measurements of the two Voyager spacecraft, as well as by Ulysses’s first fast scan (see e.g., McKibben 1975; Cummings et al. 1987; Heber et al. 1996a,b; Ferrando et al. 1996; Heber et al. 2008). Nevertheless, the study of spatial gradients was far from complete as interplanetary probes have continued to move through the heliosphere. Multi-spacecraft observations serve as a powerful tool for determining the spatial distribution of cosmic rays. Although the Ulysses mission ended its long journey in 2009, it provided a unique view of our heliosphere away from the ecliptic plane. These observations, combined with PAMELA measurements, enabled measurements of the latitudinal gradient during the 2006–2009 solar minimum (de Simone et al. 2011; Gieseler and Heber 2016) and also confirmed the importance of charge-sign dependent effects for particle propagation in off-equatorial regions of the heliosphere. High-precision Earth-orbit data provide on-orbit calibrations for other instruments flying aboard deep space missions, allowing for instruments to re-adapt to measurements for which they were not originally designed. This was the case of LEMMS instruments on-board Cassini, originally designed to study low energy particles in the Saturn magnetosphere (see, e.g., Roussos et al. 2011, 2019). The combination of LEMMS with PAMELA and AMS–02 observations provided Roussos et al. (2020) with a long-term estimation of radial intensity gradients from 1 to 9.5 AU. They found that this quantity has a solar cycle dependence; observations revealed a radial gradient value of $\sim 3.5\%/\text{AU}$ that was quasi constant between 2006 to 2014, followed by a steady drop which began in 2014 and eventually reached $\sim 2.0\%/\text{AU}$ in 2017, after the reversal of the global HMF.

### Temporal Variations: GCR Observations and Charge-Sign Dependent Modulation Prior to PAMELA

**Fig. 12:**
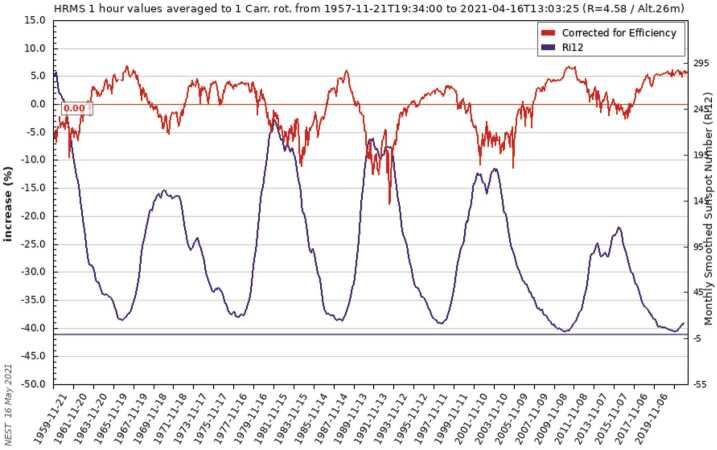
Monthly smoothed Sunspot number (violet curve) and GCR variation as measured by the Hermanus NM from 1958 to May 2021 (taken from the Neutron Monitor Data Base (NMDB) webpage)

Figure 12 displays the time variation of the Hermanus Neutron Monitor (red curve) and that of the smoothed sunspot number (violet curve). At solar maximum, the sunspot number is high and the GCR flux low and vice versa. Drift effects naturally explain the fact that in a so-called $\text{A}<0$ magnetic epoch (like in the 1960s, 1980s, and 2000s), a more peaked time profile for positively charged particles is expected compared to an $\text{A}>0$ solar magnetic epoch like in the 1970’s, 1990’s and the recent period from 2014 onward. During an $\text{A}>0$ solar magnetic epoch, the magnetic field is pointing outward over the northern and inward over the southern hemisphere, and positively charged particles drift into the inner heliosphere mostly through the polar regions and then mostly out along the Heliospheric Current Sheet (HCS).

**Fig. 13:**
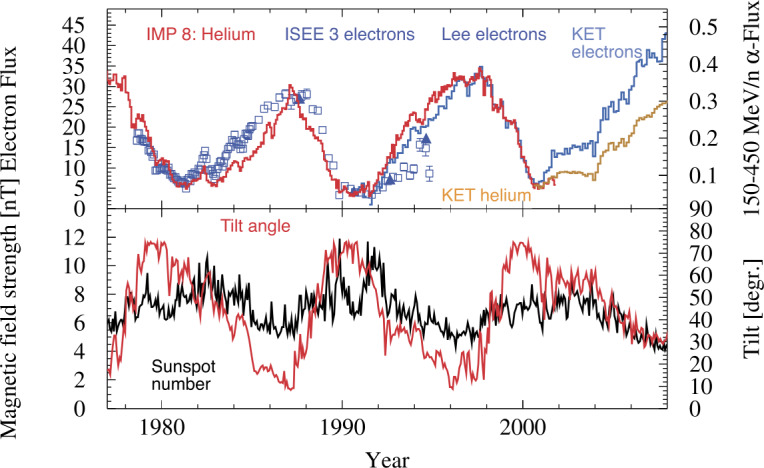
Count rate variation of GCR electrons and helium. The 70–95 MeV/nuc He flux and the $\sim1.2~\text{GV}$ He were measured at 1 AU (Earth) by the University of Chicago experiments on IMP 8 and by the Kiel Electron Telescope (KET) aboard Ulysses (Evenson et al. 1983; Heber et al. 2009) and the $\sim1~\text{GV}$ electrons by ISEE-3 and the Ulysses KET instrument. For details see text. Figure adapted from Heber et al. (2009)

**Fig. 14:**
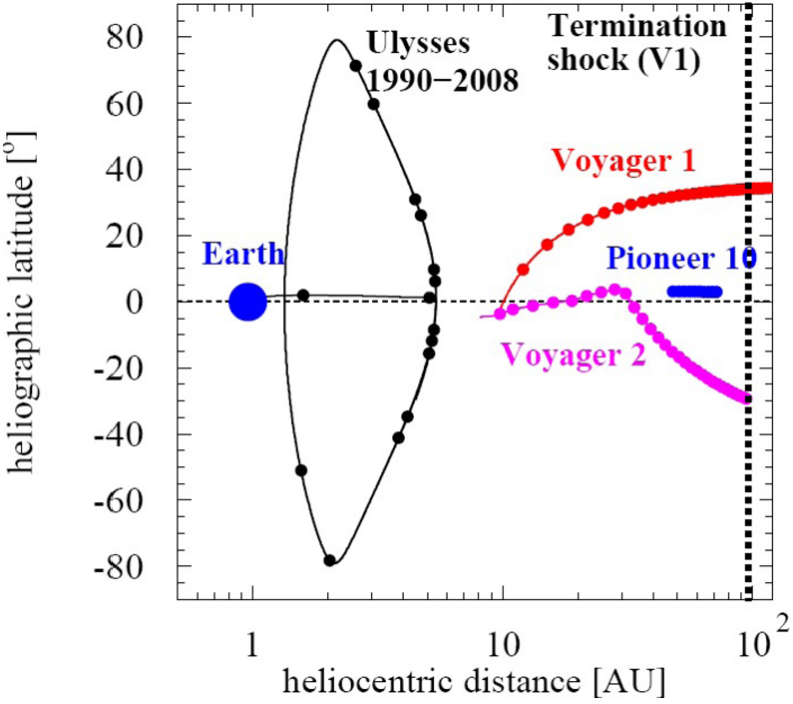
Trajectories of Voyager 1 and 2, Pioneer 10 and 11, and the Ulysses mission from 0.5 to beyond 100 AU, with distances shown on a logarithmic scale and spacecraft latitude on a linear scale (adapted from Heber and Potgieter 2006)

The upper panel of Fig. 13 displays the count rate variation of GCR electrons and helium at a rigidity of about 1 GV (Evenson et al. 1983; Heber et al. 2009) taken by ISEE-3 (electrons), IMP 8 (He) and the Ulysses KET both from 1978 to 2008 (Heber et al. 2009). The lower panel shows interplanetary magnetic field strength obtained from https://omniweb.gsfc.nasa.gov/ and the heliospheric current sheet’s tilt angle computed by the Wilcox Solar Observatory (WSO) obtained from http://wso.stanford.edu/. The time period shown includes three solar magnetic field reversals as summarized in Table 1 from Pishkalo (2019). During the first and third period the field reversed from an $\text{A}>0$ to an $\text{A}<0$ solar magnetic epoch and reversed again during the second period. Ulysses KET measurements must be disentangled for temporal and spatial variations along the Ulysses trajectory (see Sect. 6.2 and Fig. 14 for more details) before they can be compared to measurements at 1 AU. However, for helium, IMP-8 data were taken through the polarity reversal of solar cycle (SC)-23 (see Table 1). Therefore, as shown by Heber et al. (2009), KET electrons can be corrected for Ulysses’ radial variation by utilizing the radial gradient of helium in the same rigidity range and assuming a vanishing latitudinal gradient in the 1990’s. In Fig. 13 the electrons were not corrected for the latitudinal gradients during the 2000’s $\text{A}<0$ solar magnetic epoch, showing the characteristic variation in 2007 and 2008. Temporal variation of electrons and protons in this and the following SC is discussed above in detail.

**Table 1:** Epochs of the Sun’s polar magnetic field reversals in Cycles 21 to 24 taken from Pishkalo (2019). Payload for Antimatter Matter Exploration and Light-nuclei Astrophysics (PAMELA) and Alpha Magnetic Spectrometer (AMS) measurements took place during Solar Cycle 24. The dates listed here are for qualitative purposes, as there is no clear consensus amongst models concerning the exact times of the reversals (see also, e.g., Sun et al. 2015)

Solar Cycle	Begin	End
21	1979.95	1981.6
22	1990.05	1991.8
23	1999.82	2001.9
24	2012.44	2015.3

The opposite temporal variation is expected for negative charged particles – as shown in Fig. 13 and reported by many authors (e.g., Evenson et al. 1983; Clem et al. 2000; Evenson 1998; Heber et al. 2009; Aguilar 2018b). During the polarity reversal around solar maximum, the ratio of positive to negative charged particles changes in a regular pattern; specifically, the flux of positive charged particles recovers faster than the one of negative charged particles in an $\text{A}>0$ solar magnetic epoch and vice versa. Thus all charge sign-dependent observations confirm the well-established result that there are major shifts in the relative abundance of positive and negative charged particles when the solar magnetic polarity changes.

Another prediction of modulation models is a characteristic variation of the charge-sign-dependent fluxes around solar minimum when the maximum latitudinal extent of the HCS reaches low values (Heber et al. 2009). The lower panel of Fig. 13 displays the HCS tilt angle $\alpha $ (red curve) – as calculated by Hoeksema (1995) – together with the interplanetary magnetic field strength (black curve). When normalizing the electron and ion measurements near solar maximum, the fluxes evolve in SC 22 and 23 such that the fluxes approach the same values at solar minimum.

### Spatial Variations: Radial and Latitudinal Gradients of GCRs in the Heliosphere

Another prediction of numerical drift modulation models is the charge sign-dependent difference of radial and latitudinal gradients of GCRs as mentioned above (Potgieter 2014). The first evidence for positive and negative latitudinal gradients came from the Pioneer and Voyager missions in the outer heliosphere (McKibben et al. 1979; Cummings et al. 1987; Christon et al. 1986). For example, Cummings et al. (1987) report latitudinal gradients ranging from $-0.34\%/^{ \circ}$ for above 70 MeV protons to $-3.7\%/^{\circ}$ for anomalous Oxygen during an $\text{A}<0$ solar-magnetic epoch at a radial distance of 25 AU (see their Table 1). However, with the launch of the Ulysses mission in 1990, the systematic exploration of the latitudinal dependence of the GCR transport became possible. Figure 14 shows trajectories of Voyager 1 and 2, Pioneer 10 and 11, and Ulysses, plotted in a coordinate system that emphasizes the latitudinal coverage of the Ulysses mission. Ulysses’ latitudinal measurements played an important role in our understanding of energetic particle transport in the heliosphere.

**Fig. 15:**
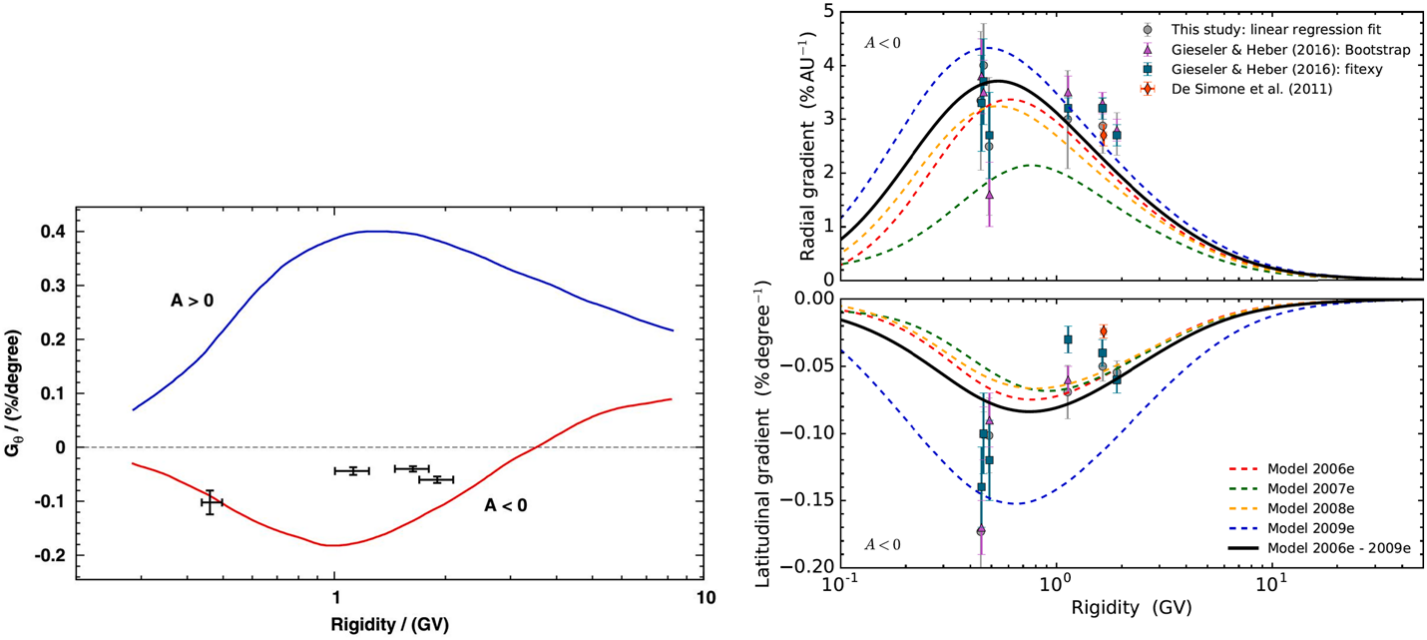
Left panel: Computed latitudinal gradients for protons during the last $\text{A}>0$ solar minimum based on Ulysses/KET measurements (blue line), and a model prediction for the $\text{A}<0$ solar minimum (red line) in comparison with the mean latitudinal gradients, plotted in black, found by Gieseler and Heber (2016). The right panel shows updated radial and latitudinal gradients computed for the previous $\text{A}<0$ solar minimum cycle for the years as indicated compared to observational values; this panel is taken from Vos and Potgieter (2016); see also references there-in

Persistent evidence of a latitudinal variation of the GCR flux were observed in Ulysses measurements from both KET and the High Energy Telescope (HET) (Simpson et al. 1992). Simpson et al. (1995) and Heber et al. (1996a,b) reported latitudinal gradients varying between 0.0 and $0.25\%/^{\circ}$ in an $\text{A}>0$ solar-magnetic epoch. During an $\text{A}<0$ solar-magnetic epoch, the latitudinal gradient of GCR protons was found to be very small with a maximum of $-0.1\%/^{\circ}$ (de Simone et al. 2011; Gieseler and Heber 2016) – that is, a factor of 4 smaller than the Voyager results mentioned above. The left panel of Fig. 15 displays the observational results from Gieseler and Heber (2016). The blue and red curves show the computed rigidity dependence of the latitudinal gradient from Potgieter and Ferreira (2001). It turned out that the modulation parameters used in this drift model from the early 2000’s could not explain the Ulysses measurements made during the previous $\text{A}<0$ solar minimum. These parameters were adjusted for the modulation conditions observed during this previous solar minimum period and used in an updated and comprehensive drift model reproducing the observations for this $\text{A}<0$ minimum as shown in the right panel from Vos and Potgieter (2016).

In order to determine the latitudinal gradient of GCR electrons observed by Ulysses without having a baseline measurement close to Earth, Heber et al. (2008) assumed that electrons and protons had the same temporal recovery (during the fast latitude scan in 2007 and 2008) and radial gradient. The latter was motivated by the finding of Clem et al. (2002), who made the first determination of the radial gradient of cosmic ray electrons in the heliosphere at rigidities of 1.2 and 2.5 GV, from 1 to 5 AU. They found that electron radial gradients in the range of 2%/AU and 5%/AU appeared to be the same as for positive particles of the same rigidity. Note that both assumptions have to be seen critically, because it is known that the electron and proton temporal recovery might be different (see above and e.g. Aguilar 2018b, and references therein) and that radial gradients have large uncertainties when determined within 5 AU so that small differences might not be found from such measurements (see e.g. Fujii and McDonald 1997, and references therein). Nevertheless, the above assumptions enabled Heber et al. (2008) to determine, for the first time, a latitudinal gradient of about $0.2\%/^{\circ}$. The results showed good agreement with that of protons during Ulysses’ first latitude scan.

### Temporal and Spatial Modulation of Galactic Cosmic Rays in the Heliosheath

When the two Voyager spacecraft explored the subsonically-flowing plasma of the heliosheath, they encountered a much different, more variable environment than the well-studied supersonic solar wind (e.g., Burlaga et al. 2006; Richardson 2015; Burlaga et al. 2018b, 2021a, and references therein). Several important open questions have since emerged concerning the nature of solar modulation in this region beyond the TS: 1) To what extent does modulation differ in the heliosheath compared to the inner heliosphere? 2) How do drifts behave in the heliosheath, and are their patterns at all similar to those of the inner heliosphere? 3) How does the modulation vary as a function of longitude and latitude? 4) To what extent are these processes influenced by the asymmetries and overall motion of the heliospheric boundaries?

**Fig. 16:**
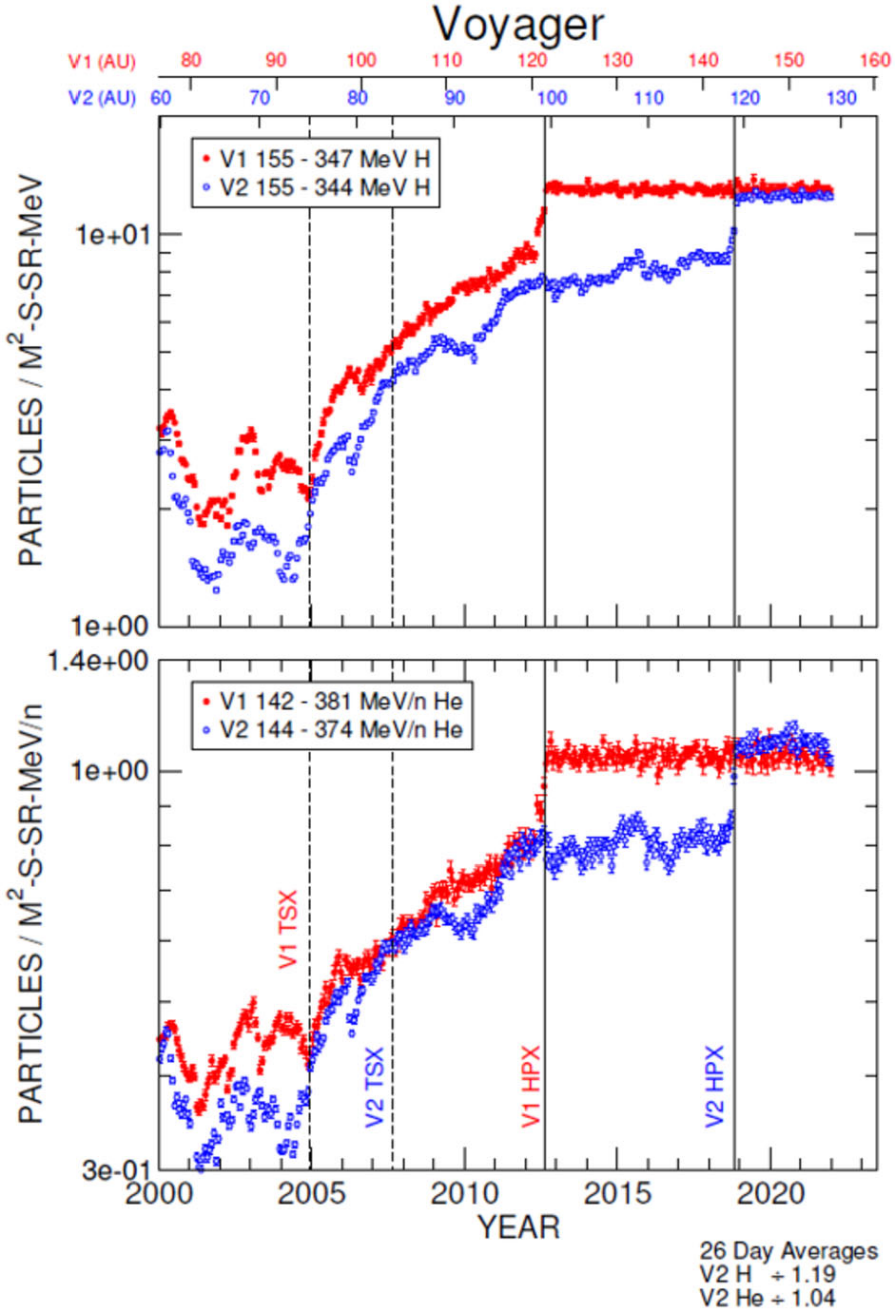
26-day averages of GCR Hydrogen (top panel) and Helium (bottom) measured by Voyager 1 (red) and Voyager 2 (blue) as a function of time (bottom) and radial distance (top) in the heliosheath and VLISM. Termination shock (TSX; dashed lines) and heliopause crossings (HPX; solid lines) are also denoted for each spacecraft. We thank the Voyager CRS team for the contribution of this figure

The Voyagers’ situ-measurements have provided many important clues about both short-term and long-term modulation of GCRs in this unusual regime, along with direct measurements of their radial distributions. For example, GCRs in the heliosheath are most strongly modulated by merged interaction regions (MIRs): large transient events that merge from a pile-up of solar events, cross the TS, and temporarily modify the heliosheath’s magnetic fields and plasma. The influence of these short-term events on GCRs in the outer heliosheath is detailed in Sect. 7. Evidence of the heliosheath’s evolution on solar cycle time scales ($\sim11$-year and $\sim22$-year patterns) is not obvious from the Voyager observations of GCRs, which are dominated by both a strong radial trend and a few transients, as shown in Fig. 16. In general, Voyager’s observations emphasize that a major element of solar modulation takes place in the heliosheath, but there is still more work to be done.

Fully investigating the above questions also necessitates an understanding of the 3D complexities of the TS-heliosheath-HP system; therefore, advances in models have also been essential for both interpreting the data and gaining insight into the above questions. Florinski and Pogorelov (2009) used a 3D MHD model of the global heliosphere under solar minimum like conditions as the background for GCR propagation. They estimated that GCR residence times in the heliosheath region were 3–6 times longer than in the supersonic solar wind. The model predicted a steady radial gradient throughout the heliosheath along the Voyager trajectories which turned out to be mostly consistent with later Voyager observations, with the exception of the rapid increases within the last 1 AU (see the next section); the predicted gradients in the heliosheath were smaller than subsequently observed (Webber et al. 2013) because the width of the heliosheath was not known at the time. Luo et al. (2013) studied the effects of the TS on the radial variation of GCR modulation using a MHD heliosphere model produced by Pogorelov et al. (2013). In computing radial profiles for 100 MeV protons in several directions, they found that flux in the heliosheath is highly dependent on longitude. Other factors also contribute, including latitude, energy, and the nature of diffusion coefficients in the heliosheath compared to the VLISM (detailed in Sect. 7). These and other examples have shown that a complete understanding of modulation in the heliosheath cannot be fully captured by Voyager’s two-point measurements.

The time-varying complexity of the plasma flows and magnetic fields, combined with pronounced asymmetries in the heliosheath – observed by the Voyagers from their TS crossings (at 94 AU and 84 AU for V1 and V2 respectively) and supported by global observations from IBEX (Stone et al. 2005, 2008; McComas et al. 2020, and references therein) provide an additional challenge for models to connect what is known from in-situ observations of the heliosheath to the current understanding of solar modulation at 1 AU.

Many models have demonstrated that the extent of solar modulation in the heliosheath is largely dependent upon the heliosheath’s thickness as well as the configuration of the TS and HP boundaries. Observations from the Voyager probes indicate that more than 50% of GCR flux reduction due to solar modulation occurs in the heliosheath. Thus, a proper model for the TS and HP is mandatory to assess the correct solar modulation level in the inner heliosphere. A representative example of such a model can be found in Boschini et al. (2019). In that work, the TS and HP are described using a time-dependent model that allows for a non-spherically symmetric shape of the heliosphere. The authors found that at high energy (for particle rigidity $>\sim3~\text{GV}$) and 1 AU the effects due to the shapes of the TS and HP are below the numerical method uncertainties. On other hand, at lower energies (e.g., those measured by Voyager) the observations cannot be re-created without accounting for the time-moving boundaries. This led the authors to conclude that, at these energies, the boundary position and the heliosphere shape cannot be simply assumed as fine-tuning parameters.

## Modulation at and Beyond the Heliopause

The heliopause is the plasma boundary of the solar system, a separatrix layer between the cold, partially ionized and strongly magnetized local interstellar medium (LISM) and the warm inner heliosheath. The existence of the heliopause, long since predicted by theory (Parker 1961; Axford et al. 1963; Baranov et al. 1976) and models (Baranov and Malama 1993; Pauls and Zank 1996; Pogorelov and Matsuda 1998), has been firmly established during the past decade through its encounters by NASA’s Voyager 1 and Voyager 2 deep space probes. The faster traveling Voyager 1 has crossed the heliopause in mid-2012 at a distance of about 122 AU from the Sun (Stone et al. 2013), while Voyager 2 had its heliopause encounter in late 2018 at a distance of 119 AU (Burlaga et al. 2019b; Stone et al. 2019).

**Fig. 17:**
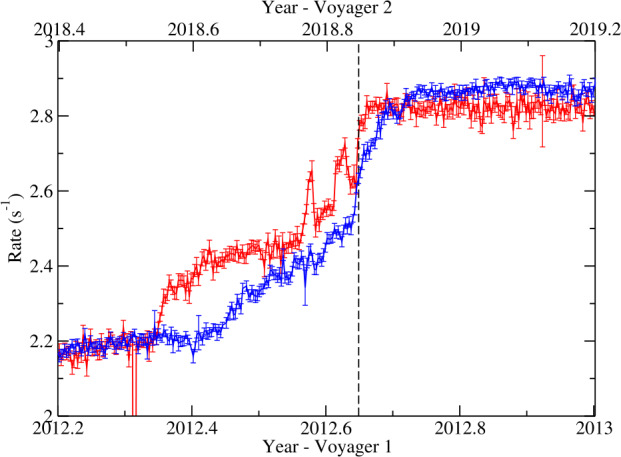
Daily averaged Voyager 1 (red) and Voyager 2 (blue) $>70~\text{MeV}$ penetrating particle count rates for $\sim10$-month periods including the spacecraft’s respective heliopause crossings (vertical dashed line). Data source: https://voyager.gsfc.nasa.gov/data.html

The heliopause is not a simple current layer similar to those routinely observed in the solar wind. Between the two plasmas, the Voyagers have uncovered a transition layer with intricate structure, revealing that the heliopause is much more complex than the isolated tangential discontinuity that it was previously believed to be. Figure 17 compares count rates of $>70~\text{MeV}$ particles from Voyager 1 and 2 during their respective heliopause encounters. Voyager 1 measured two rapid (step-like) increases in GCR fluxes. The first of these occurred 110 days before the heliopause crossing and the second was right at the heliopause. In addition to those persistent increases Voyager 1 detected two transient magnetic field increases on the heliospheric side, where GCR intensities were nearly as high as their interstellar values. Voyager 2 saw a broad magnetic barrier where the field was enhanced by a factor of $\sim 3$ compared to typical heliosheath values; GCR intensities rose gradually as the spacecraft was traversing the barrier, but increased sharply at the heliopause. The distance between the heliopause precursor events, the leading edges of the magnetic barriers and the related step increases in GCR fluxes, and the magnetic boundary itself was about 1.1 AU at Voyager 1 and 0.7 AU at Voyager 2 (Burlaga et al. 2019b). On the interstellar side, Voyager 2 detected a new region ($\sim0.6~\text{au}$) of weak GCR modulation (Stone et al. 2019). The total width of the heliopause “transition region” is therefore of the order of 1.5 AU.

Since leaving the transition layer, neither of the Voyagers observed a measurable long-term change in GCR fluxes (Cummings et al. 2016). A lack of modulation beyond the HP was theoretically demonstrated by Jokipii (2001) who argued that magnetic fluctuations responsible for energetic particle scattering are weak in the VLISM, owing to the vast disparity in size between the size of turbulent eddies (thousands of AU) and the scales on which wave-particle interactions occur (cyclotron radius, a fraction of an AU). Because GCRs travel almost scatter free along the magnetic field lines, any possible gradient would be quickly erased. Voyager 1 indeed found that the VLISM was very “quiet” in the sense that the magnetic fluctuation intensity was very small beyond the heliopause (Burlaga et al. 2015, 2018a).

This perspective was challenged by Scherer et al. (2011). Using a stochastic model of GCR transport in a simple spherical model of the heliosphere they obtained results that exhibited a significant degree of additional modulation in the outer heliosheath (OHS; the region between the bow shock and the HP). In a time-independent model, cosmic-ray deceleration in an expanding flow (such as the supersonic solar wind) is the cause of modulation, and a significant fraction of particles were found to re-enter the OHS after having spent some time in the solar wind. The results were not in agreement with subsequent Voyager observations. This could be attributed to the isotropic diffusion model used by the authors. It is very likely, however, that GCR diffusion coefficients in the VLISM are very anisotropic with the ratio of the perpendicular and parallel diffusion coefficients $\eta =\kappa _{\perp}<10^{-5}\kappa _{\parallel}$ owing to the very low intensity of magnetic fluctuations.

Subsequent work on VLISM modulation used MHD models to obtain the plasma and magnetic field background. This allows one to properly incorporate transport parallel and perpendicular to the field lines. Strauss et al. (2013b) and Guo and Florinski (2014b) performed computer simulations with similar MHD and GCR transport models, but obtained qualitatively different results. While both models featured very long parallel mean free paths in the VLISM ($10^{4}$ to $10^{5}~\text{AU}$ at 100 MeV), the former calculated that 100 MeV protons were attenuated by a few tens of % between the bow wave and the heliopause, and the latter found that GCR intensity in the VLISM was essentially constant. Kóta and Jokipii (2014) theoretically demonstrated that modulation beyond the heliopause is non-existant for plausible values of $\kappa _{\parallel}$, and that an increase in perpendicular diffusion could not lead to an increase in modulation, in contrast to the findings of Strauss et al. (2013b). Modulation at the heliopause is therefore regulated by the ratio of diffusion coefficients in the inner heliosheath and VLISM.

Zhang et al. (2015), Luo et al. (2015, 2016, 2017) reached similar conclusions; they found that if the GCR diffusion coefficients are roughly the same within a factor of a few, heliospheric modulation of GCRs will extend deep (tens to hundred AU) into the VLISM and the GCR intensity will keep rising well beyond what Voyager observed. Luo et al. (2015) were able to re-create the observations only when they dramatically decreased the perpendicular and pitch-angle diffusion coefficients in the VLISM – by several orders of magnitude – compared to those derived from the magnetic field in the heliosheath. Zhang et al. (2015) determined that, for 100-MeV GCRs, the diffusion coefficient was required to change by 2 to 3 orders of magnitude in order to agree with the Voyager observations. They also found that re-creating the GCR intensity jump required a change in the parallel-to-perpendicular diffusion ratio from 10 to 100 on the heliospheric side to $10^{6}$ to $10^{8}$ on the interstellar side. According to their simulation, such a jump could only occur outside the HP as a tangential discontinuity. Zhang et al. (2015) concluded that the GCR modulation boundary is a fraction of an AU beyond the HP, but a simple model using a heliospheric magnetic field was insufficient to reproduce the results; an accurate model must also include the interstellar magnetic field and dramatic change of diffusion coefficient at the HP (see also Luo et al. 2016, 2017).

**Fig. 18:**
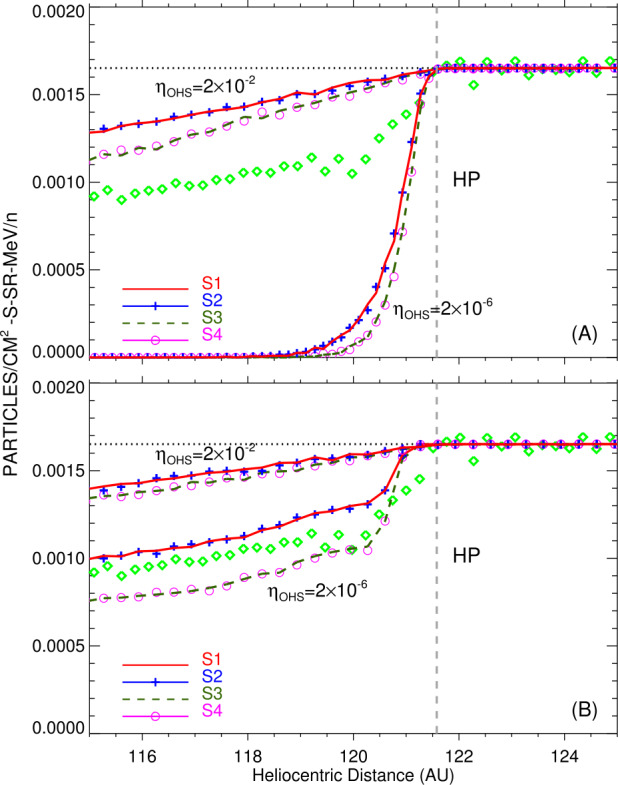
A comparison between Voyager 1’s observations of 180 MeV GCR protons (diamonds) and results from a 3D simulation of Guo and Florinski (2014b) using four different diffusion models (solid and dashed lines). Panel **A** was obtained with drift transport disabled, while Panel **B** corresponds to a simulation with the drift terms included. The figure is from Guo and Florinski (2014b)

It was also found that a very small ratio of $\eta =\kappa _{\perp}/\kappa _{\parallel}$ was required to explain the very sharp intensity increase at the heliopause. Figure 18 compares the model-derived radial intensity gradient with Voyager 1 observations for 180 MeV protons. The observations could not be reproduced using a large relative ratio $\eta =0.02$. Moreover, in using a much smaller ratio of $\eta =2\times 10^{-6}$, Guo and Florinski (2014b) found that the model overestimated the intensity increase across the heliopause unless drift effects (both along the surface of the heliopause and in the inner heliosheath) were also included. These effects can be seen by comparing the top and the bottom panels of Fig. 18.

## New Observations of GCRs in the VLISM: The Discovery of a Time-Dependent, Pitch-Angle-Dependent, Species-Dependent Anisotropy

Shortly after Voyager 1 crossed the heliopause, it made an unexpected discovery about the pitch-angle distribution of cosmic rays in the VLISM. The phenomenon was first reported by Krimigis et al. (2013) via the Low Energy Charged Particle Experiment (LECP). Although the expected isotropic and mostly uniform distributions of GCR protons ($\gtrsim211~\text{MeV}$) were observed in the $0^{\circ}$ and $45^{\circ}$ pitch-angle viewing sectors of their rotating bi-directional telescope, the $90^{\circ}$ sector revealed statistically significant and smoothly varying episodes of cosmic-ray intensity depletion (see also Krimigis et al. 2019). The Cosmic Ray Subsystem (CRS) also observed these events in their $\gtrsim20$ and $\gtrsim70~\text{MeV}$ proton-dominated rates (median energies of $\sim500~\text{MeV}$). From 2012.65 up to 2018.0, three distinct episodes were observed, as reported by Rankin et al. (2019b).

**Fig. 19:**
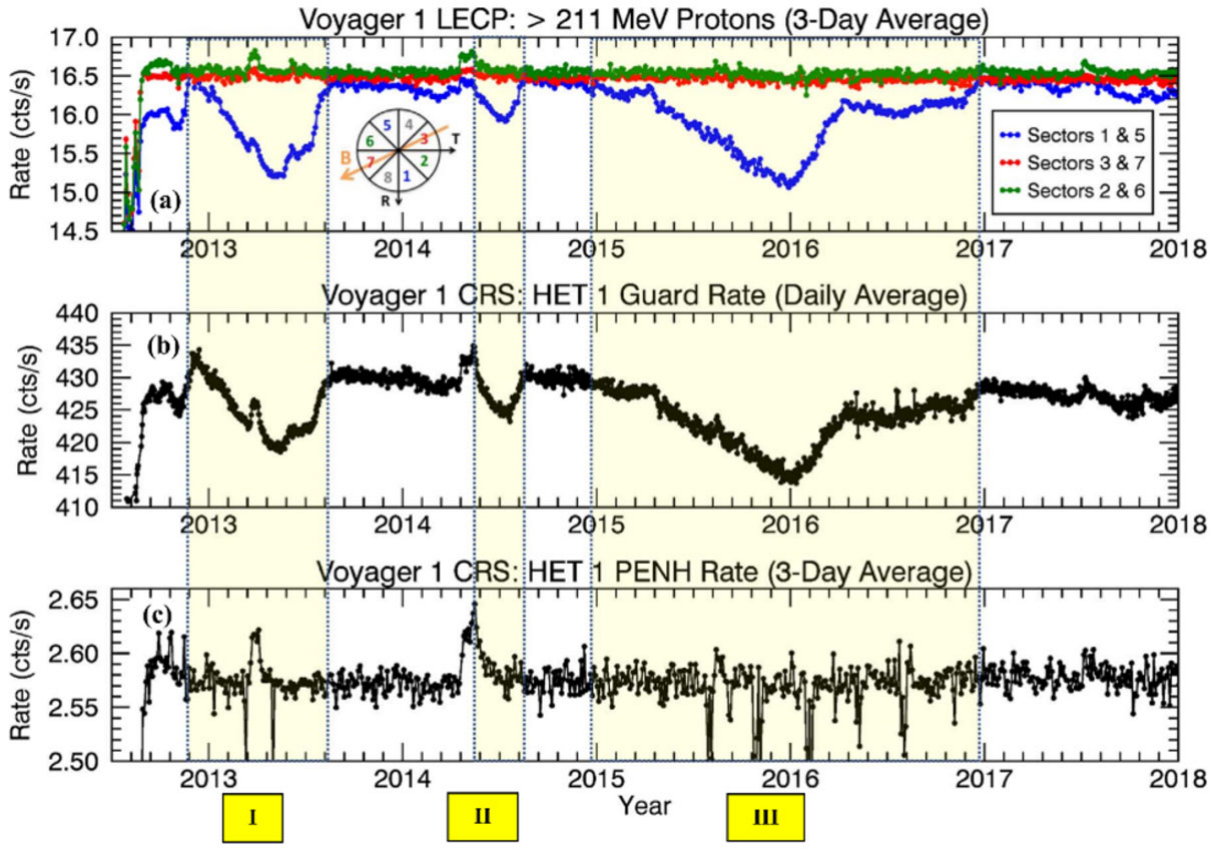
V1 observations of GCR counting rates in the VLISM detected by LECP (**a**), and CRS (**b**, **c**). Three large anisotropy events (shaded yellow) occurred between 2012.65 (shortly after the HP crossing) and 2018. Events I, II, and III lasted $\sim265$, $\sim100$, and $\sim630$ days, respectively. (**a**) LECP’s $>211~\text{MeV}$ proton channel reveals the events’ directionally-dependent nature (e.g., circular diagram, with Sectors 1 and 5 perpendicular to the field), while (**b**) CRS’s omnidirectional detectors (HET 1 Guard Rate; $\gtrsim20~\text{MeV}$; proton-dominated) reveal a time profile marked by very high statistical accuracy. (**c**) For nominal spacecraft orientations, the body-fixed CRS telescopes do not typically view the anisotropy, as indicated by the HET 1 PENH rate shown here ($\gtrsim70~\text{MeV}$; proton-dominated). However, occasional pointing do allow the telescopes to temporarily view the $90^{\circ}$ pitch angle sector, as evidenced by the periodic dips. Short-lived GCR intensity enhancements also accompany these long-duration periods of depletion and are indicative of remote connections to several solar-transient-induced shocks (further addressed in Sect. 10). Figure from Rankin et al. (2019b)

These unusual events were characterized by small changes in intensity (up to 3.8% reduction viewed by omni-directional counters), they were also remarkably long lasting ($\sim100$ to $\sim600~\text{days}$) – see Fig. 19. Since the CRS telescopes are body-fixed, Rankin et al. (2019b) relied on a series of magnetometer calibration rolls and offset pointing maneuvers^1^ to evaluate the extent of the pitch angle distribution. They confirmed that the affected distribution was centered on $90^{\circ}$ ($\pm 8.6^{\circ}$) in pitch angle space, and characterized by a broad and shallow depletion region – on average $22^{\circ}$ wide and 15% deep.

**Fig. 20:**
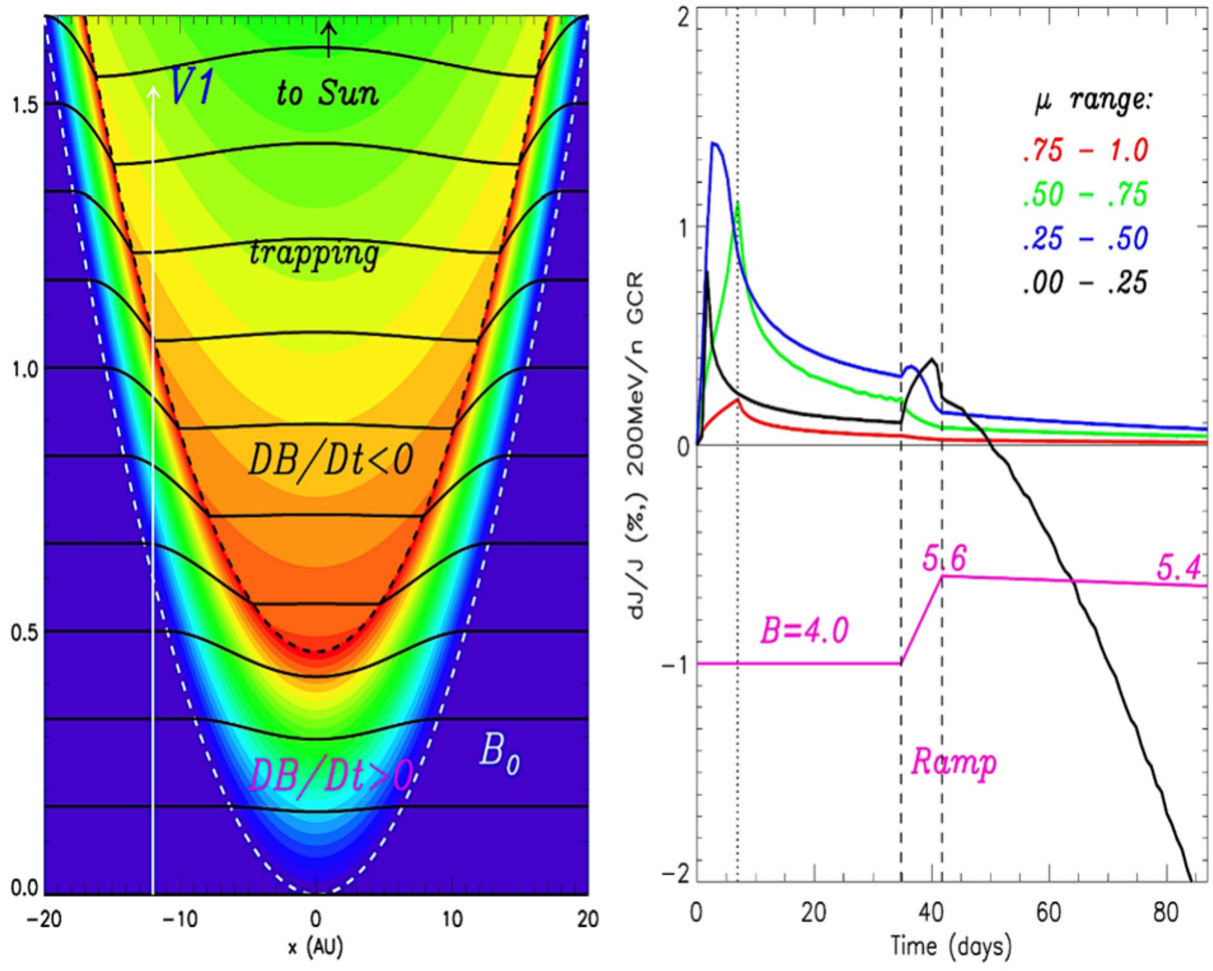
Numerical results from Kóta and Jokipii (2017)’s adiabatic cooling mechanism applied to a simple parabolic shock. The left panel depicts the simulated magnetic structure downstream of a shock as it moves outward into the interstellar medium at just above the Alfvèn speed ($40~\text{km}\,\text{s}^{-1}$). As a shock passes over Voyager, the spacecraft first encounters a compression region characterized by enhanced magnetic fields ($\text{DB/Dt} > 0$; between the two dashed lines) followed by a cooling region characterized by adiabatically-expanding fields (weak fields shown in blue, strong fields shown in red). The right panel displays the simulation result for 200 MeV cosmic rays interacting with a simple spherical-shell compression that is smoothly increasing over time (magenta). Responses were simulated for 4 pitch-angle segments, $\alpha $ (where $\mu = \cos \alpha $), each $25^{\circ}$ wide. Particles with $75^{\circ}$ to $90^{\circ}$ pitch angles ($\mu = 0.00$ to 0.25) undergo a clear intensity reduction, in agreement with observations. Figure adapted from Kóta and Jokipii (2017)

So far, the most plausible explanation for these events was that proposed by Jokipii and Kóta (2014), who suggested that the anisotropy arose due to the trapping and cooling of energetic particles in the magnetic fields downstream of the weak shocks observed by the Voyager magnetometer in VLISM (see, e.g. Burlaga and Ness 2016; Gurnett et al. 2013, 2015, 2021; Mostafavi et al. 2022). In a follow-on study, Kóta and Jokipii (2017) numerically demonstrated that the adiabatically-expanding fields more effectively trapped and cooled large-pitch-angle particles (thereby producing the anisotropy events), while particle acceleration at the compressed magnetic fields of the shock’s boundary could explain short-lived ($\sim25~\text{days}$) cosmic-ray intensity enhancements typically preceding the shocks (Fig. 19c). Results from the above-described adiabatic heating and cooling model are shown in Fig. 20. As the spacecraft nears the VLISM shock, it first encounters the gradual compression ($\text{DB/Dt} > 0$) of the shock’s boundary. In this region of enhanced magnetic fields, some fraction of GCRs are accelerated, leading to the formation of the precursor increases. Upon crossing the shock, the spacecraft then enters the downstream region of slowly-expanding, adiabatic fields ($\text{DB/Dt} < 0$), in which particles with the largest pitch angles (e.g., near $90^{\circ}$) are the most effectively trapped and cooled.

**Fig. 21:**
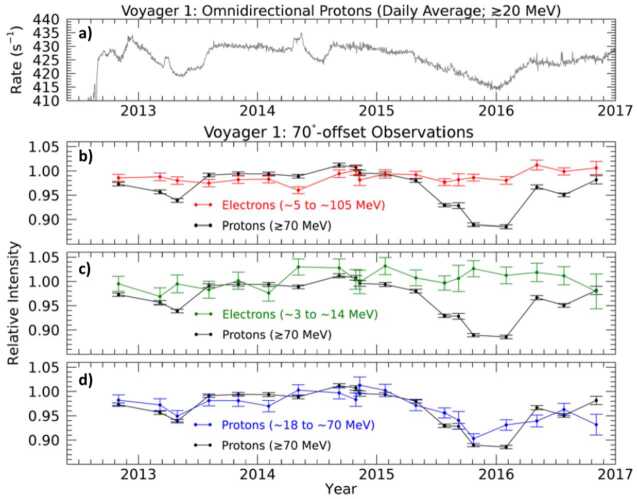
Observations from CRS on V1 reveal a species dependence in the episodes of GCR anisotropy. (**a**) the HET1 omnidirectional ($\gtrsim20~\text{MeV}$; grey) and (**b**–**d**) bi-directional protons ($\gtrsim70~\text{MeV}$; black) show prominent decreases in intensity when the telescope fields of view overlap with $90^{\circ}$ pitch angles during $70^{\circ}$-offset re-pointing maneuvers. (**d**) A clear signature is also evident in low-energy protons ($\sim18$ to $\sim70~\text{MeV}$; blue). In contrast, (**c**) neither low-energy electrons ($\sim3$ to $\sim14~\text{MeV}$; green), nor (**b**) those of similar energy ($\sim5$ to $\sim105~\text{MeV}$) exhibit much of a response, implying that the effect cannot be attributed to energy dependence. Moreover, the protons of (**c**) and electrons of (**d**) are viewed on the same telescope, while the electrons of (**b**) are recorded by a telescope that is more directly aligned with $90^{\circ}$ pitch angle, so the differences cannot be simply explained by viewing direction. Figure from Rankin et al. (2020)

Another unexpected finding is that, while the anisotropic intensity changes are evident in the protons, similar-energy electrons remain mostly unresponsive, as shown in Fig. 21. In presenting these findings, Rankin et al. (2020) quickly ruled out pointing direction and species dependence as plausible culprits and went on to explore the following 5 possibilities: (i) ineffective trapping, (ii) ineffective cooling, (iii) drifts, (iv) turbulence-induced scattering, and (v) alternative sources of scattering. The first two topics addressed the anisotropy mechanism itself: could it be that the interstellar shocks less effectively trapped or cooled the electrons? This explanation did not seem viable for several reasons. For example, the precursor “shock spikes” were clearly present in each species, signifying that both electrons and protons readily interacted with shock boundaries. The authors also ruled out ineffective cooling, as the steeper shape of the VLISM spectrum (recall Sect. 2, Fig. 1; Cummings et al. 2016) implied that electrons should undergo more effective cooling than protons, resulting in a greater, not lesser intensity change. A third possibility was that drifts, due to their charge-dependent influence on the particle propagation paths, could play some role in the formation (or hindrance) of the anisotropy for one species and not the other. However, this, too, was ruled out because curvature-gradient drifts – the type which dominates in the VLISM – have zero divergence and therefore could not directly contribute to particle energy loss (Jokipii et al. 1977). While drifts could still influence GCRs in some other way, some additional mechanism would also be needed to fully explain the observations. Concerning turbulence and scattering, it seems plausible that electrons could be more easily scattered than protons, thereby erasing their pitch angle distributions. However, the negatively-sloped magnetic power spectrum (Burlaga et al. 2015, 2018a; Zank et al. 2017, 2019) reveals turbulence amplitudes at resonant wave numbers that are 2 to 3 orders of magnitude larger for the lowest-energy protons compared to the highest energy electrons used in the Rankin et al. (2020) study, implying that protons – not electrons – should be more efficiently scattered by ambient fluctuations in the VLISM. Nevertheless, turbulence may still impact the formation of the GCR anisotropy in some other way. For example, it may contribute to the effective trapping of protons. Giacalone and Jokipii (2015) used an isotropic turbulence model to demonstrate that suprathermal protons having near-$90^{\circ}$ pitch angles could be effectively mirrored and trapped by the ambient turbulence of the VLISM. The results were used to provide an alternative explanation for formation of the IBEX ribbon (Zirnstein et al. 2020). Although the Voyager anisotropies result from energy losses (rather than gains) and affect GCRs at much higher energies (few to hundred MeVs instead of keVs) the role of turbulence in the formation of these events in GCR protons merits further investigation. As for electron scattering – several authors have found the local turbulence conditions to be appreciably modified by the VLISM shocks, generating a different frequency spectrum than observed during quiet times (Fraternale et al. 2019; Zank et al. 2019). In a multi-scale, high resolution (48 s cadence) study of V1 turbulence observations in the VLISM, Fraternale and Pogorelov (2021) found evidence of significant large-scale fluctuations, small-scale intermittency, and turbulence related to the shocks. They also found that the magnetic energy flux was significantly larger than reported by prior studies (e.g., Burlaga et al. 2018a, and references therein), which led the authors to suggest that the resulting high-frequency turbulence – likely caused by PUI instabilities – could potentially isotropize $\sim1$ to 100 MeV electrons. This too, is an interesting topic for further study. Lastly, Rankin et al. (2020) considered other mechanisms beyond the ones described above; they argued that the best mechanism to explain their observations would most likely: (i) depend on mass or charge, (ii) enable scattering through $90^{\circ}$ pitch angle near the resonant gap, and (iii) as a result of effective pitch-angle scattering, increase the probability for electrons to escape the magnetic trap and thereby prevent effective cooling. They further suggested that electric fields – particularly electromagnetic ion cyclotron waves – were a likely candidate to fulfill many of these conditions.

So far, a promising mechanism has been proposed to explain the pitch-angle anisotropy in GCR protons (Kóta and Jokipii 2017) and several reasonable possibilities have been presented to account for the lack of response in electrons. However, many aspects of the observations remain yet unexplained. For example, the location, timing, and recovery of the events is not entirely consistent (see Sect. 10 for further discussion), and why analogous intensity changes fail to manifest in the electrons is still an open question. New events seen by Voyager 1 and Voyager 2 will undoubtedly lead to further understanding, but, as conveyed by Rankin et al. (2020), the theoretical and modeling communities are also encouraged to “push deeper into the explanation of these surprising and therefore fundamentally important observations.”

## Galactic Cosmic Rays Perturbed by Transients in the Solar Wind

The intensity variations of GCRs through the heliosphere (and beyond) are caused by the temporal evolution of the environment in response to activity from the Sun. So far, this review has addressed long-term and large-scale variations that evolve with the 11-year solar cycle and change as a function of radial and latitudinal location in the heliosphere (Sects. 3–6). In the VLISM, these effects are no longer present, but it is also not a place of quiescent, undisturbed plasma (Sects. 7 & 8). In fact, as the proton pitch-angle anisotropy events demonstrate, the Sun still influences the VLISM in surprising ways – not so much by the presence of physical material, but rather by the influence of shorter-lived transients which begin their journey near the Sun.

### Cosmic Ray Transport Modified by Co-rotating Interaction Regions and Forbush Decreases in the Inner Heliosphere

Co-rotating interaction regions (CIRs) are the cause of the 27-day recurrent intensity modulation of cosmic rays that has been observed for many decades (Duggal et al. 1981; Burlaga et al. 1984; Richardson et al. 1996; Rouillard and Lockwood 2007). These recurrent structures of the solar wind are produced when a high speed solar-wind stream from a coronal hole overtakes slow wind from the equatorial regions (Crooker et al. 1999; Gosling and Pizzo 1999; Gazis 2000). This leads to a formation of a forward and reverse shock pair with a tangential discontinuity called the stream interface (SI) in between that separates the two streams. A sector boundary corresponding to the crossing of the heliospheric current sheet (HCS), is embedded in the slow wind ahead of the SI. Physical factors that can influence GCR propagation in a CIR include plasma compressions, magnetic field enhancements, turbulence generated by the stream interaction, and magnetic sector boundaries that correspond to HCS crossings. Regions of enhanced magnetic field and turbulence tend to sweep up the particles as the CIR travels outward, leading to a local enhancement ahead of the CIR or inside its low speed stream. The HCS provides an efficient inward route for positive ions during the negative solar minima (a drift effect), and is expected to establish a negative latitudinal gradient of cosmic rays. However, if drifts were chiefly responsible for GCR modulation inside CIRs, larger variations would be expected during the times of the negative magnetic polarity, which is the opposite of what is observed (Richardson 2004). Stream interfaces tend to present obstacles for cosmic rays by inhibiting magnetic field line meandering across the HCS which could lead, in some cases, to energetic particles pileups near the reverse shock (Intriligator et al. 2001).

Recently, the AMS collaboration Aguilar (2021a) reported periodicities of 27-days, 13.5 days and 9 days in the daily proton fluxes measured by AMS in the period of time from May 2011 to the end of October 2019. As first observed in 1938, recurrent variations with a period of 27 days, corresponding to the synodic solar rotation and at multiple of that frequency (e.g. periods of 13.5 and 9 days) are related to the passage of corotating interaction regions originating from one or more coronal holes of the Sun (Modzelewska and Gil 2021). Until the AMS measurements the general idea was that the strength of the periodicity steadily decreases with increasing rigidity of cosmic rays, differently in solar maximum and minimum (Gil and Alania 2013). AMS measured a 27-day significant periodicity with 95% confident level only from 2014 to 2018 with a rigidity dependence significant up to 20 GV that varies in different time intervals. The 9-day and 13.5-day periodicities are visible in 2016, their strength unexpectedly increases with increasing rigidity up to $\approx10~\text{GV}$ and $\approx20~\text{GV}$ respectively, and then decreases with increasing rigidities.

Modzelewska et al. (2020) reported on PAMELA and ARINA measurements made of the 27-day intensity variations in GCR proton fluxes in 2007–2008. These data sets allow for the first time a study of time profiles and the rigidity dependence of these variations observed directly in space in a wide rigidity range from 300 MV to several GV. They found that the rigidity dependence of the amplitude of these variations cannot be described by the same power-law at both low and high energies. A flat interval occurs at rigidity $R = 0.6$ to 1.0 GV with a power-law index of $-(0.13\pm 0.44)$ for PAMELA, whereas for above 1 GV the power-law dependence is $-(0.51 \pm 0.11)$.

**Fig. 22:**
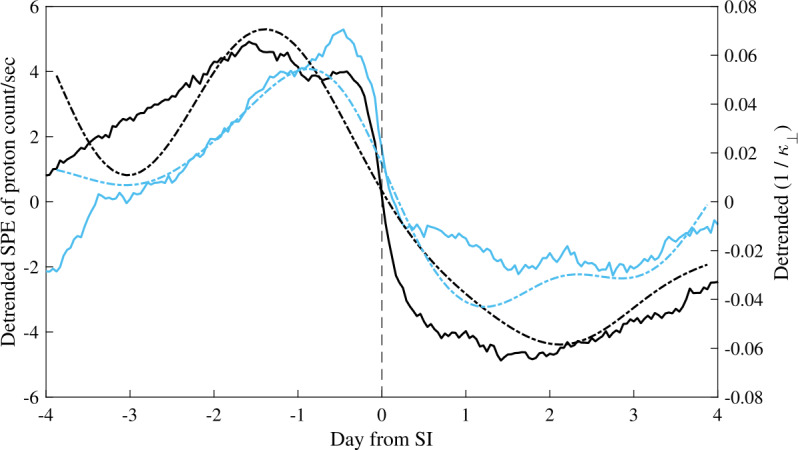
Superposed epoch analysis of ACE/CRIS proton count rates (solid lines) and the perpendicular diffusion coefficient inferred from turbulence measurements (dashed lines) between four days before and four days after the passage of the SI that was used as the zero epoch. Black lines correspond to the 2007–2008 period ($\text{A}<0$ solar minimum), and blue lines are for 2017–2018 ($\text{A}>0$) period. From Ghanbari et al. (2019)

Studies based on superposed epoch analysis (SPE) and NM data favor associations with HCS crossings (El-Borie 2001; Thomas et al. 2014), while those using high temporal resolution spacecraft data show that compressions near stream interfaces are mainly responsible for intensity depressions (Richardson 2004). Thomas et al. (2014) additionally showed that while modulation by strong compression CIRs is independent of the sense of the HCS crossing, modulation by weak compression CIRs has different pattern at AT (away-to-toward) and TA (toward-to-away) crossings and in different magnetic polarity cycles. More recently, Ghanbari et al. (2019) examined the relationship between GCR intensities during CIR passages and diffusion coefficients that were calculated based on measurements of the variance and correlation length of the magnetic fluctuations using the OMNI data set. They found that the temporal profiles of $>120~\text{MeV}$ proton fluxes from the CRIS instrument on ACE closely mirrored the behavior of the perpendicular diffusion coefficient. This result is reproduced in Fig. 22 showing SPE analysis of proton fluxes and $\kappa _{\perp}$ during 2007–2008 and 2017–2018 that correspond to the two most recent solar minima. The value of $\kappa _{\parallel}$ was of the order of $10^{22}~\text{cm}^{2}\,\text{s}^{-1}$ for both periods, with a depression right at the SI, while $\kappa _{\perp}$ was two order of magnitude smaller, increasing starting a day before the SI and remaining relatively large in the fast solar wind. The correlation between $\kappa _{\perp}$ and the GCR counts implied that perpendicular diffusion had the dominant effect on cosmic rays in a typical CIR.

Guo et al. (2021a) attempted to disentangle the drift and diffusive effects by performing SPE analyses with respect to both SI and SB crossings. They also studied a set of isolated HCS crossings without a nearby stream boundary. It was found that cosmic-ray profiles at isolated HCS crossings peaked at the zero epoch unlike the events with a SI nearby that exhibited as step-like behavior. The peak during the $\text{A} > 0$ period was twice that for the $\text{A} < 0$ which conforms with the general expectation that drift effects are more prominent during positive cycles when particles are drifting inward along the surface of the HCS.

**Fig. 23:**
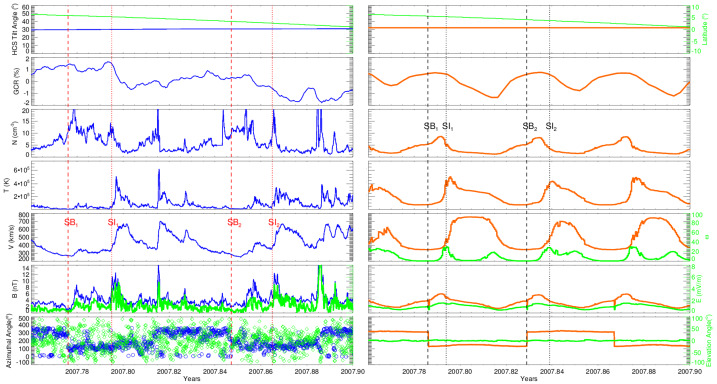
Left panels: temporal variations of the HCS tilt angle, $\sim2~\text{GV}$ proton intensities based on neutron monitor data, solar-wind number density, temperature, speed, magnetic field strength and its azimuthal angle for one particular 50-day period during 2007. Right panels: corresponding quantities obtained in a computer simulation. Solar-wind stream interfaces and sector boundaries are marked with dotted and dashed vertical lines, respectively. Figure reproduced from Guo and Florinski (2016)

CIR modulation has been the subject of much computer modeling, primarily using prescribed periodic velocity and magnetic fields of a tilted rotating dipole (Kóta and Jokipii 1991; McKibben et al. 1999; Alania et al. 2011). More recently, Guo and Florinski (2014a, 2016) have introduced a physics-based modeling framework for CIR modulation combining an MHD-derived solar wind background, cosmic-ray transport based on stochastic trajectory integration method, and a propagation model for incompressible MHD turbulence. Simulated variations of $\sim 2~\text{GV}$ protons were generally consistent with neutron monitor measurements during 2007–2009, although the predicted intensity decreases following the SIs were more gradual than observed. Figure 23 compares observed GCR count rates and solar-wind parameters in the left panel with the simulated profiles shown on the right. Model results indicated that depressions in the GCR intensity were caused by longitudinal and radial decreases in diffusion coefficients from the slow solar wind to the fast and that drift effects were less important. Luo et al. (2020) also reported on a comprehensive hybrid-type numerical study of the effects on GCR transport in the heliosphere by a CIR, emphasizing the need for further comprehensive modelling of CIRs.

Strong transient disturbances like CIRs and Coronal Mass Ejections (CMEs) can interact with GCRs in the inner heliosphere and produce effects that last days to weeks. Temporary decreases in the GCR flux due to heliospheric disturbances – first reported by Forbush (1937) – are known as Forbush Decreases (FDs) and they are observed for particle rigidities up to tens of GV. While many advancements have been made in the understanding of FDs over the past decade, it is still an open question as to how FDs behave with rigidity and how that behavior relates to solar wind conditions. However this reliance on the products of cosmic ray showers means that the information on the GCR rigidity and species is ultimately lost. This leaves an important measurement gap for instruments such as PAMELA and AMS in the study of FDs (Bindi 2021a,b,c).

Munini et al. (2018) reported on the features of a FD that commenced on 14 December 2006, following a CME at the Sun on 13 December 2006, over a wide rigidity range (0.4–20 GV) and for different species of GCRs detected directly in space. The rigidity dependence of the amplitude and the recovery time of the FD were investigated, and for the first time, the temporal variations of the helium and electron intensities during a FD were studied. They found that the temporal evolution of the helium and proton intensities were in good agreement during the FD, but the low rigidity electrons ($<2~\text{GV}$) displayed a faster recovery as evidence of charge-sign dependence. Luo et al. (2018) reported on comprehensive 3D numerical modelling of proton and electron FDs, the first of its kind. They found that during an $\text{A}>0$ cycle, the recovery time of 1 GV protons during a FD is remarkably shorter than 1 GV electrons. This model clearly predicts a charge-sign dependent effect in the recovery time of FDs but less so for their magnitude.

From May 2011 to October 2019, AMS has measured more than hundred FD events with high precision daily proton flux from 1 to 100 GV. AMS is currently computing the electron daily analysis. Once these results will be published we it will be possible to compute complementary studies in the charge/sign behavior for several new events.

### Transient Disturbances of Cosmic Rays by MIRs and GMIRs into the Heliosheath

GCRs beyond $\sim10~\text{AU}$ are primarily disturbed by Merged Interaction Regions (MIRs) – solar wind structures characterized by high plasma densities, enhanced solar wind speeds and increased magnetic fields. MIRs form beyond $\sim5~\text{AU}$ as the result of the build-up of multiple large solar transient events, including CIRs and CMEs (e.g., Burlaga et al. 1984, 1985). In the distant solar wind and the heliosheath, these can merge into even larger-scale structures known as Global Merged Interaction Regions (GMIRs). The study of MIR and GMIR effects on GCRs has been ongoing for the past several decades (see review by Richardson 2018). Much like FDs, MIRs and GMIRs act as propagating diffusive barriers, and strong magnetic fields inside a MIR/GMIR can trap particles, thereby causing GCRs to lose energy and decrease intensity. These events can strongly influence solar modulation in the heliosheath, as their frequency increases with solar activity and they can temporarily suppress GCR recovery as the solar cycle declines (e.g., McDonald et al. 1995; Le Roux and Potgieter 1995; Ferreira and Potgieter 2004). Likewise, due to their enormous size, they can disrupt GCR response to the changing solar cycle at 1 au. Recently, Luo et al. (2019) performed a numerical study of the first GMIR event of solar cycle 24, and accurately reproduced proton flux variations observed by AMS–02; this was seen to validate the GMIR concept for influencing solar modulation.

**Fig. 24:**
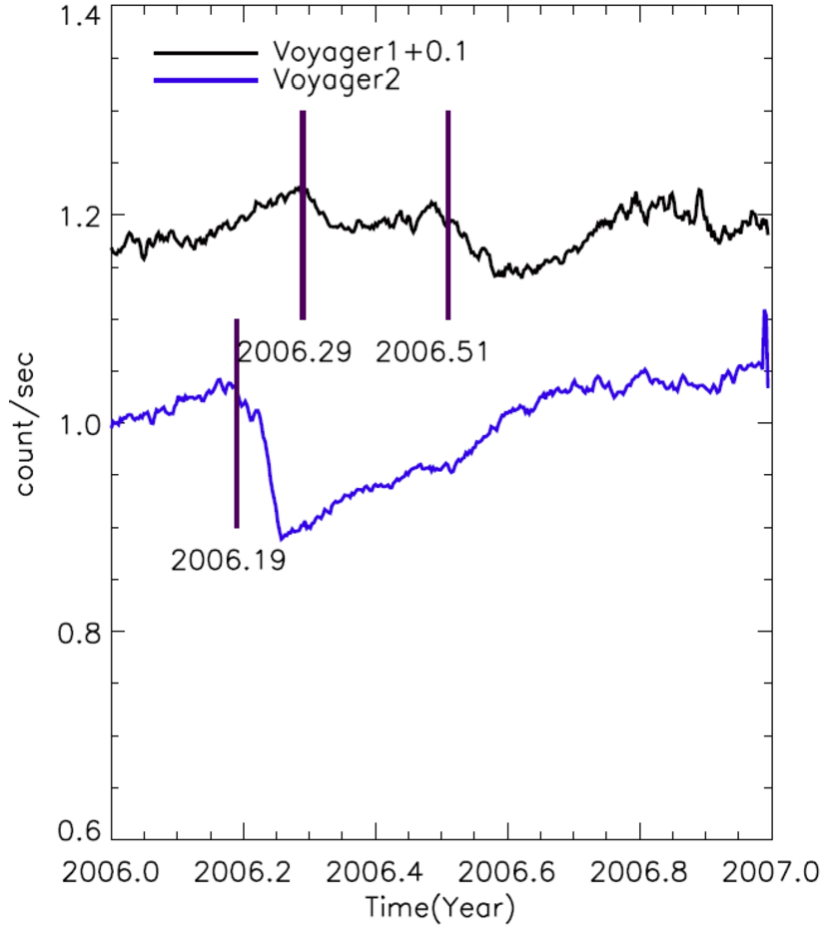
Comparison of $\text{E}>70~\text{MeV}$ Galactic cosmic ray measurements by Voyager 2 (blue) in the supersonic solar wind and Voyager 1 (black) in the heliosheath. Figure from Luo et al. (2011)

GMIRs originating from the inner heliosphere can propagate across the termination shock boundary and into the heliosheath (Burlaga et al. 2003). Direct observation of such an event was captured by the two Voyagers in 2006 when they were on opposite sides of the termination shock. A series of CMEs ejected from the Sun in September 2005 propagated and coalesced into a strong GMIR prior to arrival at Voyager 2 (Richardson et al. 2006) and Voyager 1 (Webber et al. 2007, 2009) in early 2006. Figure 24 shows the GCR intensity seen by the two spacecraft. The blue curve is what Voyager 2 saw at $\sim79~\text{AU}$, ${27}^{\circ}~\text{S}$ heliographic latitude inside the termination shock. The arrival of GMIR is characterized by enhanced solar wind velocity, density, and magnetic field observed by Voyager 2; these coincide with the decrease of GCR intensity shown here. The black curve shows observations from Voyager 1 at $\sim99~\text{AU}$, ${35}^{\circ}~\text{N}$ latitude. Unlike Voyager 2, two decreases were observed, the first of which only occurred 0.1 years later – too soon to be attributed to the GMIR’s arrival. This puzzle was pursued by Luo et al. (2011) who attributed the second decrease to local arrival at Voyager 1 and the first to the GMIR’s arrival at the termination shock. Assuming a constant GMIR propagation speed for their model, they derived a termination shock (TS) radial distance at 91 AU suggesting the TS had likely moved inward by 3 AU from Voyager 1’s crossing in December 2004 (Stone et al. 2005 and references therein). This finding supported the interpretation that Voyager 2’s assymetric TS crossing (84 au; Richardson et al. 2008; Stone et al. 2008, and references therein) was partly caused by the TS motion, a result consistent with decreasing solar wind ram pressure during that period.

## Cosmic Rays Perturbed by Propagating Plasma Disturbances in the Very Local Interstellar Medium

The influence of solar transients on the VLISM had long ago been anticipated by Gurnett et al. (1993) who remotely detected strong radio signals early on in the Voyager mission, and following each successive solar maxima. The first large radio events were particularly pronounced and took place some $\sim400$ days after several of the largest Forbush decreases ever observed, from 1983 to 1984 and 1992 to 1994. Moreover, the profiles of the radio signatures viewed by each spacecraft were very similar, despite significantly different trajectories. This led Gurnett et al. (1993) to postulate that large solar events had coalesced to form merged interaction regions (MIRs), survived out to the heliopause, and drove shocks into the surrounding interstellar material, thereby producing the electron beams responsible for the plasma emissions.

### Transients in the Heliosheath vs. the Very Local Interstellar Medium

**Fig. 25:**
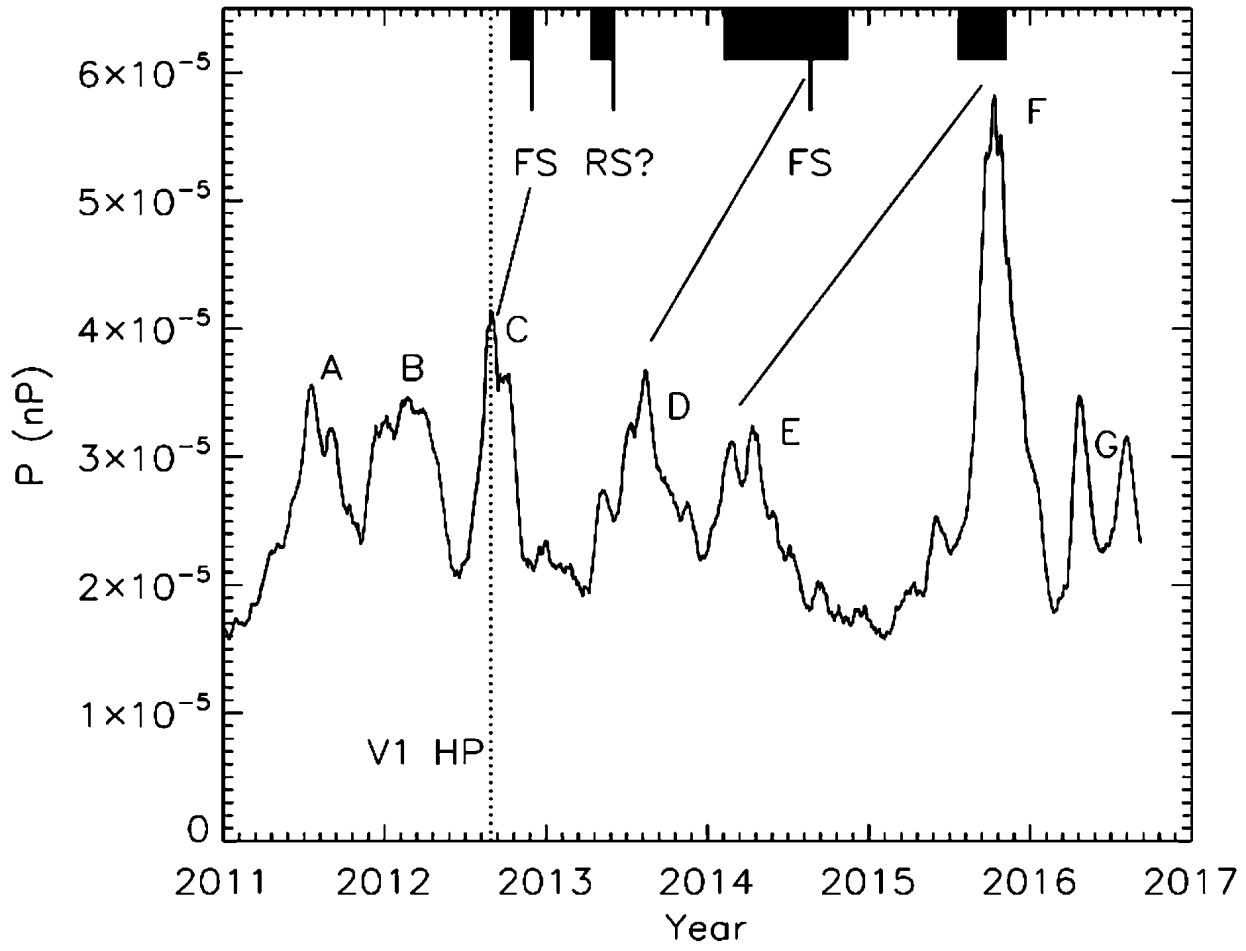
Pressure pulses caused by GMIRs in the heliosheath, as detected by the V2 plasma instrument (PWS). The dashed black line denotes the timing of the V1 heliopause crossing (“V1 HP”). The angled solid black lines serve to link V2 pressure pulses in the heliosheath to: i) candidate V1 plasma wave events in the VLISM (thick black bars at the top) and ii) possible forward (FS) and reverse (RS) shocks (thin black lines). Figure from Richardson et al. (2017)

A prime opportunity to gain more insight into GMIR interactions at the heliosphere-interstellar boundary occurred during the $\sim6$ years that Voyager 2 was in the heliosheath while Voyager 1 was in the VLISM, with the onset of solar maximum in the outer heliosheath arriving in mid-2012. Figure 25 shows 5 possible GMIRs observed by Voyager 2 (from 2012.5 to 2016.5), identified by (Richardson et al. 2017) as candidates for disturbances that could eventually be seen by Voyager 1. The events in the heliosheath were marked by $\sim50\%$ to $\sim300\%$ increases in plasma pressure, followed by pronounced decreases in cosmic ray intensity (magnetic field data were lacking during this time). By comparing arrival times of GMIRs in the heliosheath to the timing of plasma oscillations and shocks in the VLISM, (Richardson et al. 2017) concluded: “the data seem consistent with the hypothesis that the pressure pulses observed at [Voyager 2] are driving the transients observed in the [V]LISM by [Voyager 1].” This conjecture has also been supported by many data-driven models (e.g., Kim et al. 2017; Washimi et al. 2017; Guo et al. 2021b; Richardson et al. 2022; Mostafavi et al. 2022). The largest of these pressure pulses (observed by Voyager 2 in the heliosheath around late 2015; event “F” of Fig. 25) was soon afterward associated with a large pressure front seen by IBEX via enhanced ENA emissions that arrived at 1 AU in late 2017 (McComas et al. 2020, and references therein) that inflated the heliosphere. The event was also observed in situ by Voyager 1 in the VLISM, via an unusually long magnetic disturbance interpreted as a “pressure front” (Burlaga et al. 2019a, see next subsection) and a similarly broad anisotropic disturbance of GCR’s.

**Fig. 26:**
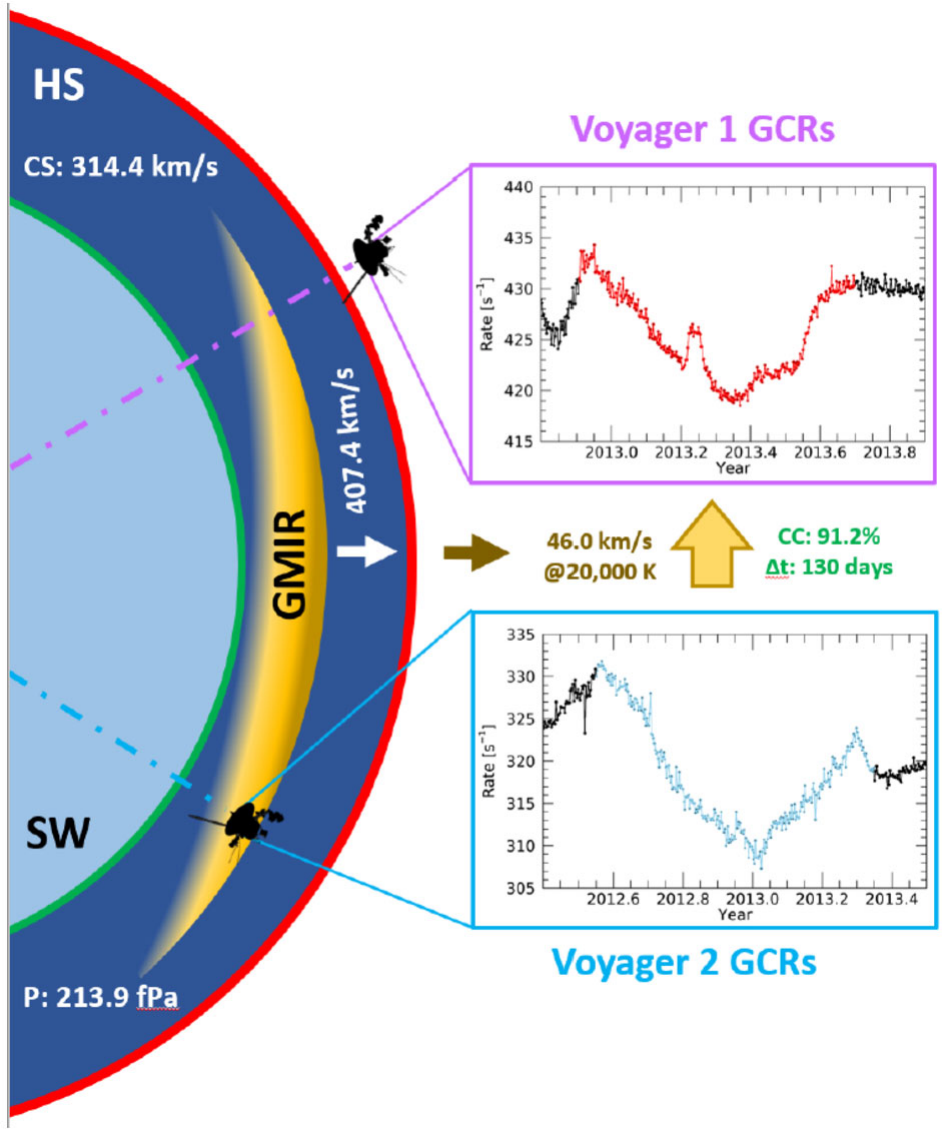
Schematic of a GMIR that grew from a coalescence of solar events in the interplanetary medium, traveled through the heliosheath (HS), interacted with the heliopause (HP) and transmitted a pressure wave into the VLISM. Such an event likely traversed the heliosphere and produced the data observed on the right: (bottom) an isotropic response from GCRs that was detected by V2 in the heliosheath (blue plot), followed by (top) the anisotropic disturbance viewed by V1 in the VLISM 130 days later. The heliosheath sound speed (CS) and pressure (P) shown here were calculated assuming a VLISM temperature of $20,000$ K. However, a range of values was presented by the authors and more relevant values informed by recent knowledge acquired from V2’s HP crossing are discussed in the text. Figure from Rankin et al. (2019a)

Rankin et al. (2019a) found direct evidence of such an event in mid-2012 that passed Voyager 2 in the heliosheath, and 130 days later, generated a causally-related disturbance at Voyager 1 in the VLISM; observations at each spacecraft showed remarkably similar time profiles in the GCRs, with a high coefficient of correlation (91.2%). They used the time delay, in-situ measurements of the magnetic field and plasma, average speed in the heliosheath, and well-supported assumptions about the plasma in the VLISM to derive previously unmeasured heliosheath quantities – a range of plausible sound speeds and total effective pressures. Figure 26 presents a summary of their findings; results were obtained for a range of VLISM temperatures,^2^ the values discussed (and shown in Fig. 26) focused on a nominal temperature of 20,000 K, which was consistent with modeling expectations at the time (Zank et al. 2010). More recent observations from V2 have revealed somewhat higher VLISM temperatures: 30,000 to 50,000 K (Richardson et al. 2019), so it is the 40,000 K results that are highlighted here (ref. Rankin et al. 2019a, Tables 1 and 2). The assumption of $T_{\text{VLISM}} = 40{,}000~\text{K}$ yielded speeds of $v_{VLISM} = 51.6 \pm 4.9~\text{km}\,\text{s}^{-1}$ for the pressure pulse in the VLISM and $v_{HS} = 392 \pm 40~\text{km}\,\text{s}^{-1}$ for the GMIR in the heliosheath. From the latter, an average sound speed of $C_{HS} = 299 \pm 31~\text{km}\,\text{s}^{-1}$ and total effective heliosheath pressure of $P_{total} = 242 \pm 50~\text{fPa}$ were derived. In the context of previously-determined partial pressures (inferred from Voyager and IBEX; summarized in Table 1 of Rankin et al. 2019a), constituents of this total pressure are as follows: $\text{thermal}= 1.3\%$, $\text{magnetic}= 2.2\%$, solar wind dynamic $\text{pressure}= \sim 12\%$, ACRs and $\text{GCRs}= \sim 21\%$, pickup $\text{ions}= \sim 45\%$. The remaining $18.5\%$ is not presently accounted for – possibly due to electrons (Fahr and Heyl 2020b,a), which are not directly measured by Voyager or IBEX. Recent studies of pressures in the heliosheath show general agreement (Dialynas et al. 2020; Reisenfeld et al. 2021), but achieving more direct measurements of the total pressure in the heliosheath is a goal for future missions (see also, Dialynas et al. [2022] this journal).

Meanwhile, the present results pose an intriguing question: Given the surprisingly similar GCR time profiles, why are the transient-disturbed fluxes isotropic in the heliosheath but highly anisotropic in the VLISM? An interesting solution was put forth by Zhang and Pogorelov (2020), who simulated the response of GCRs to GMIRs in the heliosheath and VLISM, employing a Vlasov-Fokker-Planck equation to allow for the transport of particles having significant anisotropy. Their model demonstrated that the Forbush-decrease-like disturbance of GCRs by GMIRs in the heliosheath vs. the pitch-angle limited behavior in the VLISM could be fully accounted for by differences in the scattering parameters of the two regimes.

**Fig. 27:**
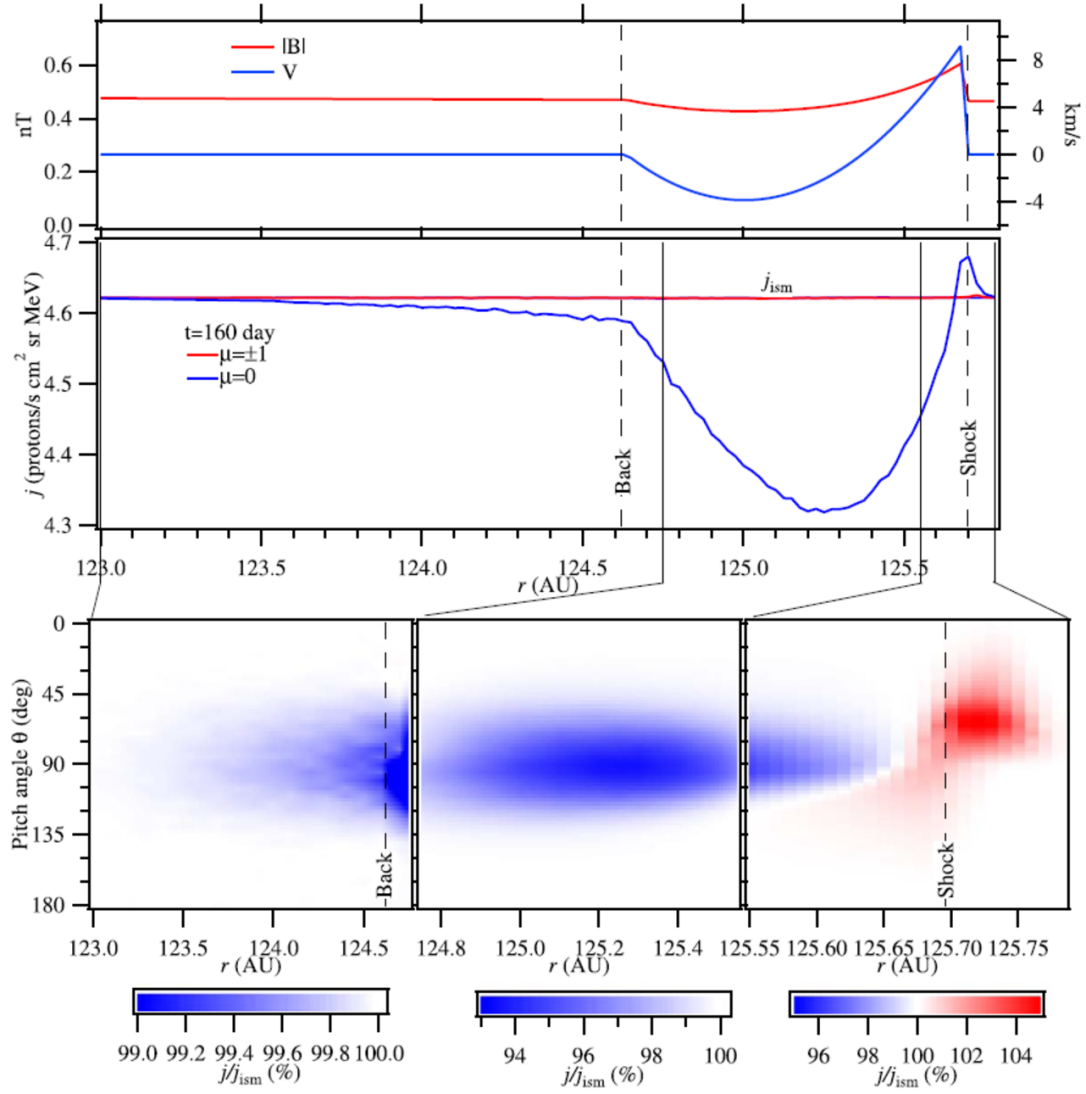
Radial profiles of the magnetic field strength, plasma speed, intensity of 100 MeV GCR protons and pitch-angle distribution proposed by a weak pressure pulse is in the VLISM. Detailed variations of the pitch-angle distribution are shown in three segments with different radial distance and magnitude scales. The GMIR propagates radially at 40 km/s and has a thickness of 1.06 AU. Figure from Zhang and Pogorelov (2020)

Figure 27 exemplifies how the GCR intensity and pitch angle distribution vary in the vicinity of a GMIR-induced plasma perturbation (GMIR) in the VLISM. In this regime, due to weak scattering from the VLISM turbulence, GCRs of different pitch angles do not mix, and they experience different degrees of modulation. As in the theory of Kóta and Jokipii (2017), particles having pitch angles away from ${90}^{\circ}$ only briefly interact with the pressure pulse so their energies remain essentially unchanged. Meanwhile, those with near $\theta ={90}^{\circ}$ pitch angle ($\mu = \cos{\theta}\approx 0$) can be trapped inside the rarefied magnetic fields. Energy loss causes the trapped particles to significantly drop their intensity, resulting in a dumbbell pitch-angle distribution with reduced intensity near ${90}^{\circ}$ pitch angle (blue regions of Fig. 27). In contrast, GCRs in the heliosheath – due to strong levels of turbulence in the heliospheric magnetic field – experience significant mixing so that particles of all pitch angles get effectively trapped behind the GMIR and undergo modulation.

### Manifestation of Transients in the Very Local Interstellar Medium

The above-described GMIR-caused plasma perturbations disturb the otherwise quiescent VLISM in many interesting ways. Manifestations of these Sun-caused transient disturbances have been detected by multiple instruments on both Voyager spacecraft in the form of: (i) short-lived GCR intensity enhancements and long-lived anisotropic depletion episodes (recall Sect. 8), (ii) electron plasma oscillations and radio emissions, and (iii) unusual shocks and magnetic disturbances. For example, several weak, quasi-perpendicular, sub-critical laminar shocks have been observed by the Voyager 1 magnetometer (Burlaga et al. 2013; Burlaga and Ness 2016), which are typically preceded by roughly month-long cosmic-ray intensity enhancements (“shock spikes”; e.g., Fig. 19c of Sect. 8), and locally-generated plasma emissions. Gurnett et al. (2015) formulated a model to describe the relationship and timing of these collective events, known as the “foreshock model”. This model explained the GCR and plasma events as precursors to interstellar shocks, analogous to those typically observed in the solar wind and upstream of planetary bow-shocks.

**Fig. 28:**
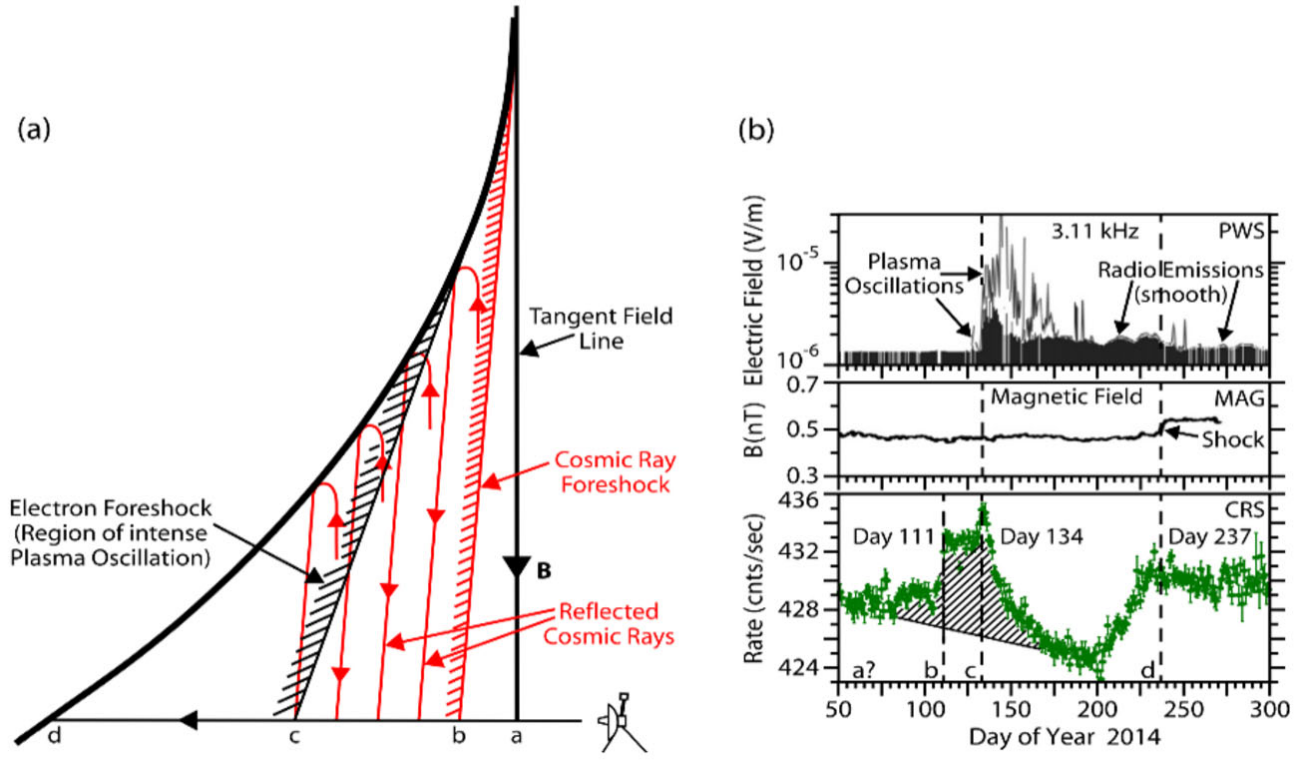
Summary of Gurnett et al. (2015, 2021)’s Foreshock Model, illustrating (**a**) the timing of events encountered by Voyager 1 and Voyager 2 in the VLISM prior to the eventual arrival of a shock, and (**b**) linked to a specific series of transient disturbances viewed by Voyager 1 in 2014. The resulting VLISM pressure wave has been associated with: (i) GCR intensity enhancements and depletions (e.g., bottom, green), (ii) plasma oscillations and radio emissions (e.g., top, black), and (iii) magnetic disturbances and shocks (e.g., middle, black). Figure from Gurnett et al. (2015)

As illustrated by Fig. 28, each event’s arrival time is determined by the spacecraft’s connectivity to the shock front. Reflected and accelerated GCR electrons and protons in the “cosmic ray foreshock region” arrive first due to their higher energies, followed by electron plasma oscillations (few to hundred eV), and in many (but not all) cases, the shock itself. The theory was further developed and tested by Gurnett et al. (2021), who used the timing of GCR electron enhancements ($\sim5$ to $\sim100~\text{MeV}$) along with the plasma oscillation onset times to provide the first calculation of electron plasma beam energies in the VLISM. The determined values were around 20 to 100 eV ($\sim50~\text{eV}$ on average) – similar to those known to drive Type-II radio bursts in the solar wind.

In addition to the GCR and plasma events, several types of magnetic disturbances have also been observed by both Voyagers in the VLISM. These include the above-mentioned weak, quasi-perpendicular, subcritical shocks that are a few thousand times thicker than their 1-AU counterparts (see also, Mostafavi et al. 2022) as well as an unusual event characterized by a $\sim35$-day rise in magnetic field strength at Voyager 1 beginning $\sim2016.95$, followed by a $\sim340$-day decay ending in $\sim2017.97$ (Burlaga et al. 2019a). While the former were likely the result of pressure pulses transmitted by GMIRs from the heliosheath into the VLISM, the latter has been associated with a time-delayed response to a considerable increase in solar wind dynamic pressure. This remarkable phenomenon was first reported by the Interstellar Boundary Explorer, who detected it in late 2017 and instigated a large-scale inflation of the heliosphere that continues to this day (McComas et al. 2018; Zirnstein et al. 2018; McComas et al. 2020). A second unusual magnetic “pressure front” has been recently reported by Burlaga et al. (2021b), but association with a specific period of solar activity is yet to be determined.

**Fig. 29:**
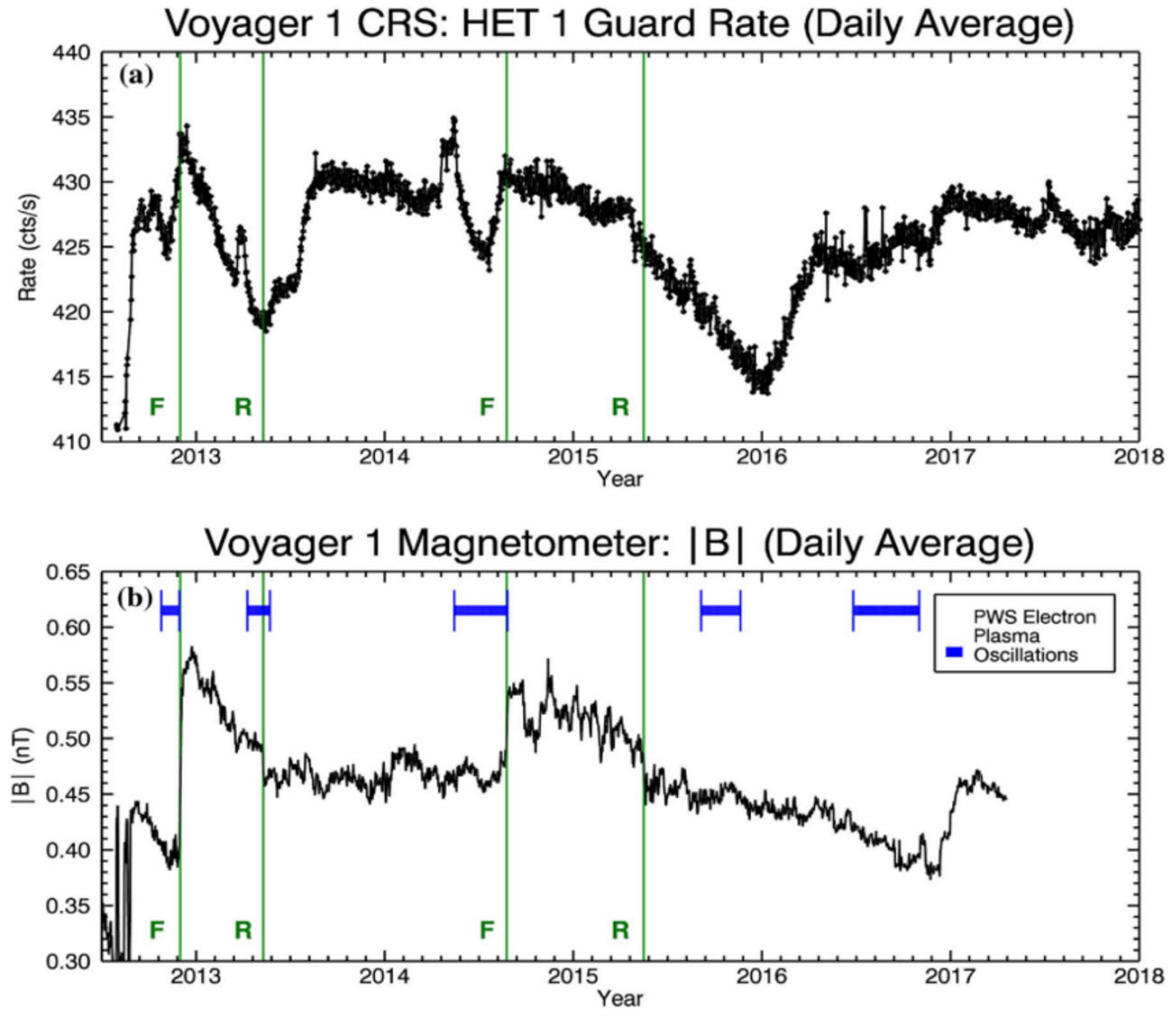
Time-varying responses of $\gtrsim20~\text{MeV}$, proton-dominated GCR’s (**a**) and the magnetic field (**b**) to solar-caused plasma disturbances in the VLISM. The green lines indicate onset times of forward (F) and (possibly) reverse (R) shocks reported by the V1 Magnetometer (Burlaga and Ness 2016). The horizontal blue bars (**b**; top) indicate the timing of electron plasma oscillations reported by PWS (Gurnett et al. 2015)

As one might expect, episodes of GCR anisotropy have been associated with each of these magnetic disturbances. Rankin et al. (2019b) contended that the first large event detected by V1 in 2013 provided compelling evidence to support the adiabatic cooling mechanism proposed by Kóta and Jokipii (2017). Following an abrupt shock passage around $\sim2012.9$, enhanced fields encountered by Voyager 1 weakened until $\sim2013.35$ and then stabilized to $\sim0.46~\text{nT}$; see Fig. 29. At the same time, the particle intensities declined and achieved a few-day minimum. Finally, since the fields were no longer expanding, the GCRs recovered – evidently the shock had passed and V1 was no longer connected particles trapped downstream. The episode beginning in 2015 (recall region II of Fig. 19), displayed a similar, but not identical behavior; the field weakened in two phases while GCRs responded with a two-step intensity decline (from $\sim2014.65$ to $\sim2015.35$ and $\sim2015.35$ to $\sim2016.0$).

However, the data also reveal noticeable inconsistencies with the proposed theory. For example, no obvious magnetic disturbance preceded the 2014 episode (recall region II of Fig. 19), and the local fields appeared neither expanded nor compressed. An equally puzzling process began in 2016, in which GCR intensities recovered despite a continued drop in the local magnetic field. Attempts to explain/resolve these issues have been made by several authors, although no clear solution has emerged. Rankin et al. (2019b) explored several configurations of where trapping and cooling might occur with respect to the spacecraft. They concluded that Voyager could view the anisotropy both in-situ and remotely, because its region of formation, “in addition to being affected by temporarily compressed fields from traveling disturbances, could also be affected by the presence of a steady state enhanced magnetic field near the heliopause”. In other words, the permanently draped field around the heliopause as observed by IBEX via the ribbon (McComas et al. 2009; Zirnstein et al. 2016) likely plays some role on the magnetic trapping process. Hill et al. (2020) agreed that the draping of the magnetic fields around the heliopause could be essential to the anisotropy’s formation, but they came to a different conclusion about where the trapping might occur: “the anisotropy-causing physical process that suppresses intensities at $\sim90^{\circ}$ pitch angles relies upon [GCRs] escaping from a single compression in the draping region, not on [GCRs] trapped between two compressions.” Therefore, although the mechanism introduced by Jokipii and Kóta (2014), Kóta and Jokipii (2017) is consistent with the observations in several important ways, there are still many aspects of the events that are not yet fully understood.

## Cosmic Rays on Larger Scales: An Astrophysical Perspective

In-situ observations of GCRs in the VLISM, an increased understanding of their modulation by the Sun and its activity, as well as newly-obtained insights about the global heliosphere have also led to intriguing results from an astrophysical perspective. Detailed examples can be found elsewhere in this volume (see, e.g. Brandt et al. 2022; Herbst et al. 2022; Kleimann et al. 2022; Linsky et al. 2022; Richardson et al. 2022), but two topics reviewed briefly here are as follows: (i) interactions of TeV to PeV GCRs with the structure of the heliosphere, and (ii) GCR acceleration and propagation processes in the Local Interstellar Medium (LISM).

Concerning (i), the magnetic field between the heliopause and the heliospheric bow wave differs from that of the pristine LISM. The trajectories and energies of TeV to PeV cosmic rays can be altered by the large-scale electromagnetic fields of the heliosphere, which produces a significant cosmic ray anisotropy when viewed from Earth. After correction for heliosphere modulation effects, the inferred TeV cosmic ray anisotropy in the LISM manifests as a nearly pure dipole aligned with the local interstellar magnetic field. The heliosphere induces a medium-scale anisotropy from the dipole anisotropy, leaving various imprints of its structure in cosmic ray anisotropy maps, detailed below.

Regarding (ii), PAMELA, AMS–02, CREAM, CALET, DAMPE and NUCLEON experiments have recently performed precise measurements of primary and secondary cosmic rays fluxes in the GeV-TeV energy regime, integrated over long time periods. These measurements reveal a number of very interesting spectral features such as spectral breaks and bumps with previously unexpected behaviors which show clear departures from a single power-law spectra of GCRs (see e.g. Lipari 2018). The measured spectral features likely point to the presence of local cosmic ray accelerators which could be related to either past activity of supernovae from the star-forming complexes in the solar vicinity (Zucker et al. 2022) or energetic astrospheres associated with nearby pulsars or massive stars (see review by Herbst et al. 2022). The secondary over primary ratios of GCRs allows for better constrains of propagation parameters, such as diffusion coefficients and their energy dependence in the LISM. High energy nuclei fluxes and their relative flux ratios integrated over years are key measurements for understanding the acceleration and propagation processes of GCRs in LISM as well as assessing astrophysical antimatter backgrounds.

### TeV Cosmic Ray Anisotropy

**Fig. 30:**
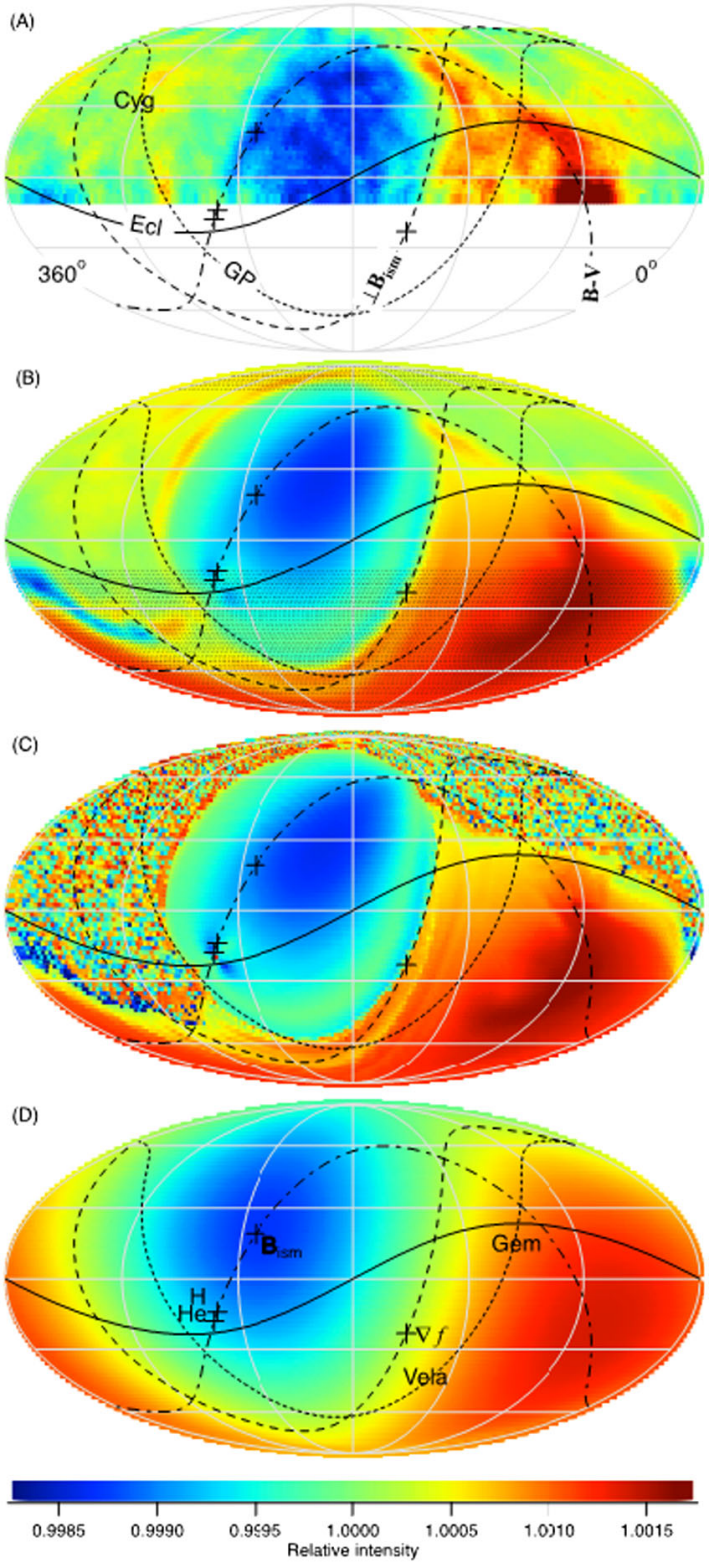
Anisotropy maps of 4 TeV cosmic ray relative intensity in the celestial coordinate system. (**A**) Tiber $\text{AS}\gamma $ measurements at Earth sampled at $2^{\circ}\times 2^{\circ}$ resolution and averaged over $5^{\circ}$ radius (Amenomori 2006). (**B**) Model calculation of anisotropy expected at Earth after the distortion by the heliosphere in $5^{\circ}$ averages with light shading outside of the Tibet $\text{AS}\gamma $ field of view. (**C**) Model calculation of anisotropy with $2^{\circ}\times 2^{\circ}$ resolution without average. (**D**) Inferred large-scale anisotropy in the local interstellar medium if the heliosphere is not present. The curves represent the locations of the ecliptic (ECL), Galactic plane (GP), Hydrogen deflection plane (B-V) and the plane perpendicular to the local interstellar magnetic field $({\perp \mathbf{B}}_{ism})$. The crosses indicate the directions of local interstellar helium inflow (He), deflected hydrogen inflow (H), local interstellar magnetic field $(\mathbf{B}_{ism})$ and cosmic ray density gradient $(\nabla{f})$. The labeling Syg, Vela, and Gem indicates the directions of Cygnus, Vela, and Geminga supernova remnants, respectively. Figure from Zhang et al. (2020)

The anisotropy of TeV-energy GCRs arriving at Earth has been measured by many air shower experiments, such as Tibet $\text{AS}\gamma $, IceCube, Super-Kamiokande, Milagro, ARGO-YBG, HAWC, and others (Amenomori 2006, 2010; Abdo et al. 2009; Guillian et al. 2007; Abbasi et al. 2011; Di Sciascio 2012; Abeysekara et al. 2014). Several experiments have obtained enough particle counting statistics to construct two-dimensional anisotropy sky maps (e.g., Amenomori 2006; Abeysekara et al. 2019) which reveal a small anisotropy of ${10}^{-4}$ to ${10}^{-3}$ in relative intensity amplitude. As shown by the example of Fig. 30, the observed anisotropy maps present a pattern that is quite complicated, with many medium- (tens of degrees) to fine-scale (a few degrees) structures superimposed on an overall large-scale pattern. A number of mechanisms have been proposed to explain the origins of these non-uniform enhancements of GCRs, such as: (i) natural propagation from specific point sources in the Galaxy (Amenomori 2006; Abbasi et al. 2011), (ii) local variations of turbulence in the LISM (Giacinti and Sigl 2012; Ahlers and Mertsch 2015; Battaner et al. 2015; Giacinti and Kirk 2019), and (iii) modulation by the heliosphere. Evidence for the latter was initially found by the Tibet team, who noticed that some of their observed anisotropy features were aligned with the so-called hydrogen deflection plane (HDP) – the plane containing the original LISM (neutral helium) flow vector and the flow vector of deflected neutral hydrogen caused by the solar wind (SW)-LISM interaction (Amenomori 2010). Several authors have proposed theories and performed calculations with models to explain how the heliosphere can affect the anisotropy of TeV cosmic rays (see, e.g. Desiati and Lazarian 2013; O’c Drury 2013; Schwadron et al. 2014; Zhang et al. 2014, 2020). For example, Schwadron et al. (2014) showed that the anisotropy’s global direction was consistent with the mean field derived from the IBEX ribbon, implying that GCRs propagate mainly along the field, with very little cross-field diffusion.

Zhang et al. (2014) employed a Liouville flux-mapping technique to calculate particle trajectories and simulate the TeV anisotropy, using the heliosphere simulation with Multi-Scale Fluid-Kinetic Simulation Suite (MS-FLUKSS) developed by the University of Alabama Huntsville group. Best fits to Tibet $\text{AS}\gamma $ observations enabled them to constrain values such as neutral hydrogen density and ion density in the local interstellar medium. Results are listed in Table 2. In a follow-on study, Zhang et al. (2020) found that the influence of the heliosphere’s electromagnetic fields on particle propagation could almost entirely account for the anisotropy’s medium-scale variations. The small-scale effects, they argued, were likely to come from the original fields of the LISM with a weak influence from turbulence. They additionally determined a gradient of $0.021\pm0.001\%~\text{R}_{g}^{-1}$ perpendicular to the LISM magnetic field (gyroradius $\text{R}_{g} = 254~\text{AU}$ for 4 TeV protons in a 3.5 μG field), pointing to the Vela supernova remnant and Local Bubble as possible GCR sources.

**Table 2:** Interstellar parameters as derived from fits to TeV cosmic ray anisotropy measurements by Zhang et al. (2020)

Local interstellar magnetic field vector	
Strength ${B}_{ism}$	3.5 μG
Declination $\delta _{\mathbf{B}_{i}sm}$	19^∘^
Right Ascension $\alpha _{\mathbf{B}_{i}sm}$	232.5^∘^
Interstellar neutral density $n_{H}$	$0.184~\text{cm}^{-3}$
Interstellar ion density $n_{p}$	$0.065~\text{cm}^{-3}$
4 TeV cosmic ray distribution function	
Amplitude of pitch-angle dipole $A_{1}$	0.00165
Amplitude of pitch-angle quadrupole $A_{2}$	0.00015
Density gradient vector	
Magnitude |*G*⊥|	$8.3\times{10}^{-7}~\text{AU}^{-1}$
Declination $\delta _{\mathbf{G}_{||}}$	−23^∘^
Right Ascension $\alpha _{\mathbf{G}_{||}}$	151^∘^

### GeV-TeV Cosmic Ray Leptons in the Heliosphere from Nearby Sources

Recent precise measurements of the GCR lepton fluxes by *PAMELA*, *AMS–02*, *Fermi LAT*, *DAMPE* and *CALET* have revealed a number of important spectral features. Namely, the increase of the positron fraction in the leptonic GCRs above 10 GeV measured by *PAMELA* (Adriani et al. 2009) and *AMS–02* (Accardo et al. 2014; Aguilar et al. 2019) is inconsistent with the secondary positron component produced by inelastic collisions of the observed GCR nucleons with the interstellar gas.

The origin of the positron component may be either due to the long sought annihilation/decays of dark matter candidate particles (e.g. Bertone et al. 2005) or associated with high energy positrons accelerated in the local sources like pulsars or supernova remnants (see e.g. Hooper et al. 2009; Blasi and Amato 2011; Recchia et al. 2019; Evoli et al. 2021). A spectral break at about 1 TeV and some hints of a spectral bump at about 1.4 TeV were identified in the lepton spectrum by the ground-based Cherenkov telescope *H.E.S.S.* (Aharonian et al. 2009) and the space experiments *Fermi LAT* (Abdollahi et al. 2017), *DAMPE* (DAMPE Collaboration 2017), and *CALET* (Adriani 2018). The revealed spectral features in the TeV CRs lepton regime can help to distinguish between the different scenarios and to constrain the characteristics of the possible local sources or the mass of hypothetical dark matter particles.

On the basis of a general discussion of the observed high-energy electron-positron spectra, López-Coto et al. (2018) concluded that a yet undiscovered pulsar in the Local Bubble is needed to explain the data. A few known nearby middle-aged pulsars PSR B1055-52, Geminga and PSR B06564+14 are considered as possible candidates to contribute at some level to the observed lepton fluxes (Di Mauro et al. 2019; Fang et al. 2019). Extensive multi-wavelength observations are available for the pulsars and their parallax measurements (apart from PSR B1055-52) were used to estimate the distances of $250^{+120}_{-62}~\text{pc}$ for Geminga and $288^{+33}_{-27}~\text{pc}$ for PSR B06564+14 (see e.g. Abeysekara et al. 2017). All of the three pulsars are gamma-ray sources of a few 100 kyr age, have similar spin-down power $\dot{E}\sim10^{34}~\text{erg}\,\text{s}^{-1}$ and magnetic fields $\sim10^{12}~\text{G}$ (see e.g. Posselt et al. 2015; Bîrzan et al. 2016; Posselt et al. 2017). The superb angular resolution of the *Chandra* observatory allowed us to study spatially resolved spectra of the extended synchrotron pulsar wind nebulae. The observed X-ray and gamma-ray photon spectra of pulsar wind nebulae produced by synchrotron and inverse Compton processes are used to model the spectra of GeV-TeV regime positrons and electrons which then diffusively propagated to the Solar System (e.g. Della Torre et al. 2015; Bykov et al. 2017).

Abeysekara et al. (2017) reported the detection with the High Altitude Water Cherenkov Observatory (*HAWC*) of the extended TeV emission from Geminga and PSR B0656+14. These authors proposed that the profiles of the TeV halos detected by HAWC imply a slow diffusion of particles emitting TeV gamma-rays and that these source unlikely contribute to the positron fluxes detected by *PAMELA* and *AMS–02*. More recently, Di Mauro et al. (2019) estimated the upper limit of the GCR positron flux from Geminga in the Solar System to be about 20% of that was measured. In a contrast to the idea of a slow diffusion of TeV leptons in the vicinity of the pulsars. Recchia et al. (2021) account for the transition from the quasi-ballistic to the diffusive transport regime in the vicinity of Geminga and PSR J0622+3749 and provided a good fit to the HAWC and LHAASO gamma-ray data without the suppression of the diffusion coefficient. The issue of the slow self-generated diffusion in the vicinity of CR sources certainly needs further efforts (Bao et al. 2021).

Most of the studies use two zone diffusion models of the GeV-TeV energy particle transport from the sources. Namely, the energy dependent CR diffusion coefficient as a function of the distance $r$ from a source is assumed to be $D(r,E) = D_{1,2} (E/1~\text{GeV})^{\delta _{1,2}}$ where the diffusion coefficient within the inner region $r \leq r_{b}$ is labeled by 1 and the outer region has label 2. The indexes $\delta _{1,2}$ are usually about 0.33. The boundary $r_{b}$ between the two zones is not well determined but is larger than 10–20 pc. The diffusion coefficient in the inner zone $D_{1}$ is supposed to be $\sim 10^{26}~\text{cm}^{2}\,\text{s}^{-1}$, which is very slow compared to the standard models of the global CR diffusion over galactic scales (see e.g. Strong et al. 2007, for a review) but it is consistent with the values derived from the gamma-ray profiles model suggested by the HAWC observations (Abeysekara et al. 2017). The diffusion coefficient in the outer region $D_{2} \gtrsim 10^{28}~\text{cm}^{2}\,\text{s}^{-1}$ is consistent with the average diffusion coefficient in galactic CR propagation models. To explain the recent DAMPE measurements of the proton spectra Fang et al. (2020) discussed a model where the diffusion coefficient is homogeneous in the nearby ISM (within 50 pc vicinity of the Solar system) and is very slow with $D_{1} \sim 10^{26}~\text{cm}^{2}\,\text{s}^{-1}$. The large regions with the slow CR diffusion can be produced by old extended supernova remnants or by other CRs and MHD turbulence sources. Recent study by (Zucker et al. 2022) associated the Local Bubble origin with a burst of stellar birth and supernovae about 14 Myr ago. Multiple shocks produced by supernovae could efficiently enhance MHD turbulence and regulate the CR transport in superbubbles (see e.g. Bykov and Toptygin 1987; Bykov et al. 2020). The issue of CR diffusion in the Local Bubble certainly requires further investigation.

**Fig. 31:**
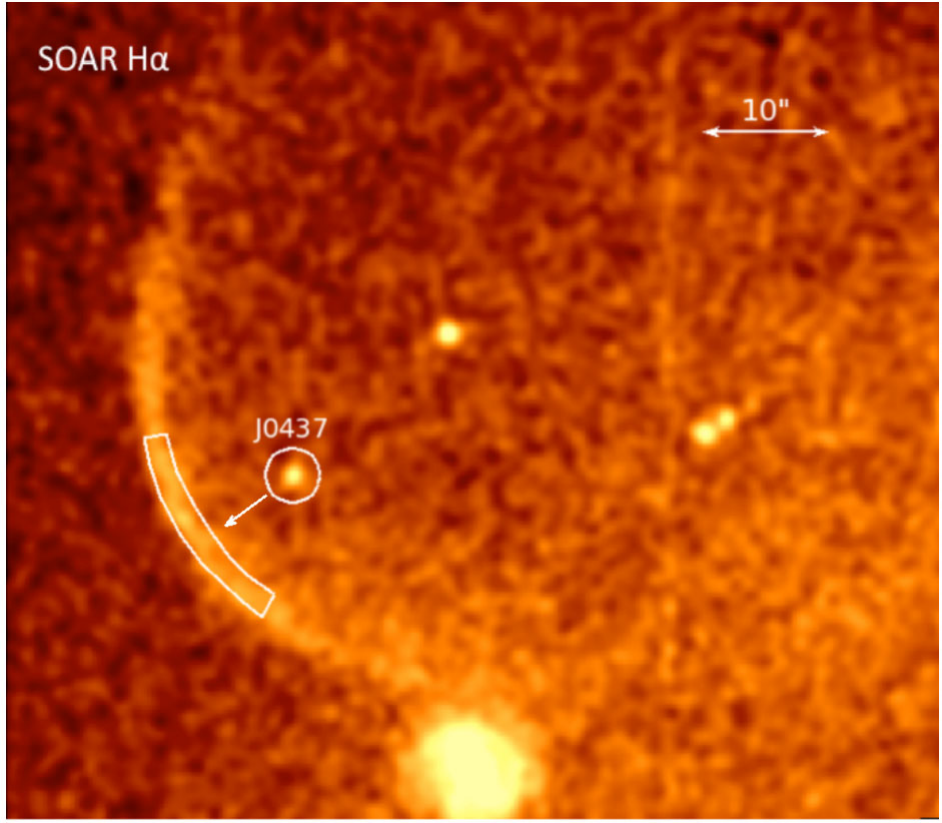
$\text{H}_{\alpha}$ image of the bow shock of PSR J0437-4715 taken with the SOAR telescope (Brownsberger and Romani 2014; Rangelov et al. 2016). The pulsar has the transverse proper velocity of about $104~\text{km}\,\text{s}^{-1}$. The image was smoothed with a 5 pixel Gaussian kernel

**Fig. 32:**
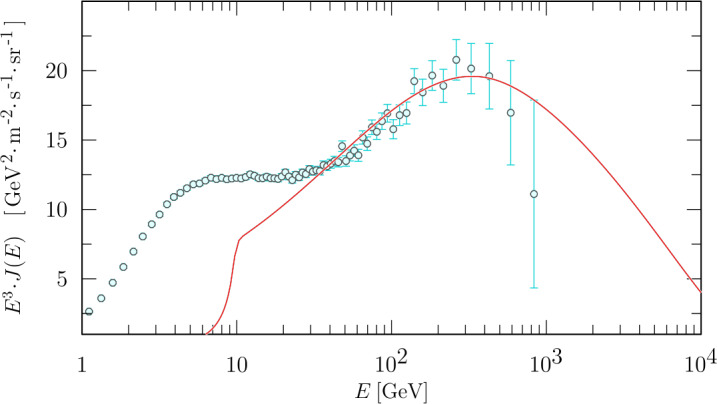
Model spectrum of cosmic-ray positrons (red) in the solar system produced by the nebula of PSR J0437-4715 after diffusion through the local interstellar medium with account of synchrotron/Compton energy losses (see Bykov et al. 2019). The observed AMS–02 spectrum (Aguilar et al. 2019) is shown in blue points

**Fig. 33:**
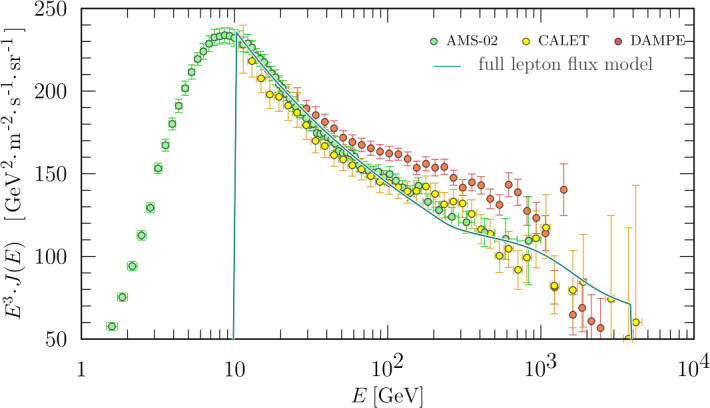
Model spectrum of CR leptons originating from the nebula of PSR J0437–4715 confronted with the data measured by AMS–02, DAMPE and CALET experiments Bykov et al. (see for details 2019)

Apart from the middle-aged pulsars discussed above there is an interesting object, the millisecond pulsar PSR J0437-4715 of a period 5.8 ms located at the distance $156.79\pm0.25~\text{pc}$ (as measured the by parallax), which is the nearest pulsar observed so far. PSR J0437-4715 is a very old recycled pulsar with a white dwarf binary companion (see e.g. Rangelov et al. 2016, and references therein). High angular resolution observations of PSR J0437-4715 with Hubble Space Telescope and Chandra by Rangelov et al. (2016) showed the presence of a bow shock type pulsar wind nebula. The $\text{H}_{\alpha}$ filter image of the bow shock is shown in Fig. 31 (Brownsberger and Romani 2014; Rangelov et al. 2016). The optical, UV and X-ray spectra of PSR J0437-4715 observed by *HST* and *Chandra* (see Rangelov et al. 2016) were used by Bykov et al. (2019) to construct a kinetic model of acceleration of positrons and electrons in the extended bow shock pulsar wind nebula. The $\text{e}^{\pm}$ pairs are accelerated at the relativistic pulsar wind termination shock and then are further accelerated in a semi-relativistic colliding MHD flow between the wind termination surface and the bow shock (Bykov et al. 2017) which is produced due to the supersonic motion of PSR J0437-4715 (see Fig. 31). The positrons and the total leptonic spectra at the Solar System simulated within the model are shown in Figs. 32 & 33 and compared to the data available from *AMS–02*, *DAMPE* and *CALET* observations. Therefore one may indeed expect that either an individual source or a combination of contributions from a few nearby pulsars may explain the observed features in electron and positron spectra.

## Conclusions

**Fig. 34:**
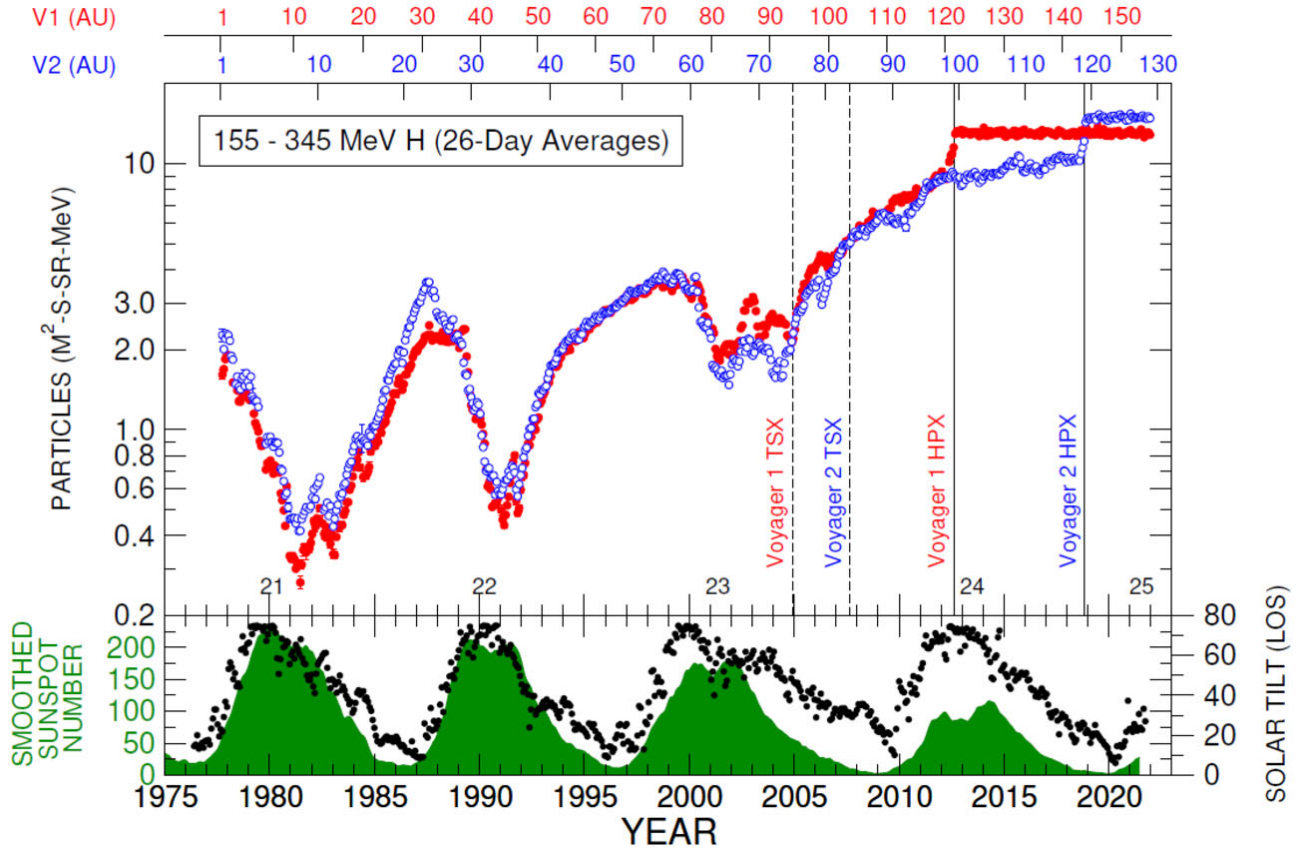
26-day averaged radial and temporal profiles of 155-345 MeV GCR Hydrogen measured along Voyager 1 (V1; red) and Voyager 2 (V2; blue) trajectories through the heliosphere and in to the interstellar medium. Termination shock (TSX; dashed line) and heliopause crossings (HPX; solid line) are denoted for each spacecraft by vertical lines. The anisotropic disturbances in the VLISM are not visible here (HPX and beyond) due to the orientation of the two telescopes, which in their nominal configuration do not view pitch angles near $90^{\circ}$. Smoothed sunspot numbers and line-of-sight (LOS) solar tilt angles measured at 1 AU are included for reference. Solar maximum peaks are marked by numbers which enumerate different epochs of the solar cycle. Voyager data are derived from PENH rates of two high-energy telescopes (HET 1 & HET 2) on the Cosmic Ray Subsystem (CRS). The smoothed sunspot numbers are from the SILSO Royal Observatory of Belgium, Brussels (https://wwwbis.sidc.be/silso/DATA/SN_ms_tot_V2.0.txt), while the computed LOS Tilt angle of the Heliospheric Current Sheet comes from the Wilcox Solar Observatory (http://wso.stanford.edu/Tilts.html). We thank the Voyager CRS team for the contribution of this figure

In the decade of work since the centennial anniversary of the discovery of cosmic rays, substantial progress has been made towards understanding fundamental cosmic ray physics and also towards broader questions in heliophysics. We have sampled and modeled diverse plasma environments, probed the global properties of the solar wind, measured how the interstellar medium influences the heliosphere, and witnessed how the Sun and heliosphere interact with their surroundings. In-situ measurements by the two Voyager spacecraft in the VLISM, as well as nearly a solar cycle of observations from PAMELA and continued high-precision measurements from AMS–02 have resulted in rich new data sets, which, combined with more sophisticated numerical models, will undoubtedly continue to yield fresh insights and exciting discoveries about cosmic-rays and their interactions with and within the global heliosphere. We have reviewed recent advances in space-based GCR observations and modeling, detailing key measurements of the spectra, large-scale, long-term spatial and temporal intensity variations from 1 AU to the heliopause, and summarizing advances in models that have reinforced the paradigms of solar modulation. We have also highlighted new measurements from the VLISM, including the first in-situ measurements of the low-energy GCR spectra (below 100 MeV), the behavior of cosmic rays at the heliopause boundary, and the discovery of weak, but durable episodes of pitch-angle dependent anisotropy. We have shown how transient events from the Sun at 1 AU can be linked to disturbances in the VLISM and have considered the story of cosmic rays near our star from a broader, astrophysical point of view. These unprecedented achievements point to the emergence of a new, global perspective, summarized by Fig. 34.

As evidenced by the two Voyager trajectories (Fig. 34; V1 in red, V2 in blue), the effects of solar modulation are clearly prominent in the inner heliosphere leading up to the termination shock (TSX; supersonic solar wind plasma), far less prominent in the heliosheath (TSX to HPX; subsonic solar wind plasma) and conspicuously absent in the VLISM (HPX onward). Occasional shorter-term transient perturbations caused by MIRs and GMIRs are also evident in both Voyagers, up to the heliopause, with more events encountered by V2 due to the arrival of solar maximum at the time of its passage through the heliosheath. Superimposed on the trend of cyclical modulation of GCRs by the solar wind is a significant gradient, which becomes more pronounced in the heliosheath and is conspicuously absent in the VLISM. Collectively, this captures much of the complexity, and particularly the limitations of interpreting single-vantage-point observations within the heliosphere. Therefore, in conjunction with multi-spacecraft observations, sophisticated modeling has been vital for filling in the gaps. Figure 34’s GCR intensities also reflect an ever-present challenge of de-coupling temporally and spatially varying effects. Despite this, it can be seen that, in general, as this high-intensity galactic radiation makes its way through the solar wind, a surprising amount of filtration occurs. For example, at 1 AU during solar minimum, roughly 70% of the GCR intensity gets filtered out, while during solar maximum (with respect to ∼10 AU), the value increases to $>85\%$ (e.g, just beyond the peak of the spectrum, in the 155 to 345 MeV energy range shown here; cf. Fig. 1). Thus, in some sense, culmination of our current understanding leads us to a startling new question – without the heliosphere, would life on earth even exist?

Indeed, this has been a historic decade in which humankind is now beginning to explore and discover our stellar neighborhood from the outside-in, rather than inside-out. But there is also still more work to be done and we look forward to future studies and missions that will undoubtedly address open questions, further current understanding, and help us gain new wisdom about our place within the galaxy.

## Footnotes

1 Multi-day re-pointing campaigns that occurred roughly every other month and continued at that frequency until 2017. These maneuvers fortuitously positioned the CRS telescope boresights so that they overlapped with the $90^{\circ}$ pitch-angle viewing direction and therefore were able to characterize the anisotropy events.

2 Rankin et al. (2019a) performed their calculations for VLISM temperatures ranging from 7,500 to 40,00 K and achieved pressure estimates ranging from $292 \pm 60$ to $252 \pm 50~\text{fPa}$.

## References

[CR1] Abbasi R., the IceCube Collaboration (2011). Observation of anisotropy in the arrival directions of galactic cosmic rays at multiple angular scales with IceCube. Astrophys. J..

[CR2] Abdo A.A., Ackermann M., Ajello M. (2009). Measurement of the cosmic ray $\text{e}^{+}+\text{e}^{-}$ spectrum from 20 GeV to 1 TeV with the Fermi Large Area Telescope. Phys. Rev. Lett..

[CR3] Abdollahi S., the Fermi-LAT Collaboration (2017). Cosmic-ray electron-positron spectrum from 7 GeV to 2 TeV with the Fermi Large Area Telescope. Phys. Rev. D.

[CR4] Abeysekara A.U., Alfaro R., Alvarez C., Álvarez J.D., Arceo R., Arteaga-Velázquez J.C., Ayala Solares H.A., Barber A.S., Baughman B.M., Bautista-Elivar N., Belmont E., BenZvi S.Y., Berley D., Bonilla Rosales M., Braun J., Caballero-Mora K.S., Carramiñana A., Castillo M., Cotti U., Cotzomi J., de la Fuente E., De León C., DeYoung T., Diaz Hernandez R., Díaz-Vélez J.C., Dingus B.L., DuVernois M.A., Ellsworth R.W., Fiorino D.W., Fraija N., Galindo A., Garfias F., González M.M., Goodman J.A., Gussert M., Hampel-Arias Z., Harding J.P., Hüntemeyer P., Hui C.M., Imran A., Iriarte A., Karn P., Kieda D., Kunde G.J., Lara A., Lauer R.J., Lee W.H., Lennarz D., León Vargas H., Linnemann J.T., Longo M., Luna-García R., Malone K., Marinelli A., Marinelli S.S., Martinez H., Martinez O., Martínez-Castro J., Matthews J.A.J., McEnery J., Mendoza Torres E., Miranda-Romagnoli P., Moreno E., Mostafá M., Nellen L., Newbold M., Noriega-Papaqui R., Oceguera-Becerra T., Patricelli B., Pelayo R., Pérez-Pérez E.G., Pretz J., Rivière C., Rosa-González D., Ruiz-Velasco E., Ryan J., Salazar H., Salesa Greus F., Sandoval A., Schneider M., Sinnis G., Smith A.J., Sparks Woodle K., Springer R.W., Taboada I., Toale P.A., Tollefson K., Torres I., Ukwatta T.N., Villaseñor L., Weisgarber T., Westerhoff S., Wisher I.G., Wood J., Yodh G.B., Younk P.W., Zaborov D., Zepeda A., Zhou H., HAWC Collaboration (2014). Observation of small-scale anisotropy in the arrival direction distribution of TeV cosmic rays with HAWC. Astrophys. J..

[CR5] Abeysekara A.U., Albert A., Alfaro R., Alvarez C., Álvarez J.D., Arceo R., Arteaga-Velázquez J.C., Avila Rojas D., Ayala Solares H.A., Barber A.S. (2017). Extended gamma-ray sources around pulsars constrain the origin of the positron flux at Earth. Science.

[CR6] Abeysekara A.U., the HAWC Collaboration the IceCube Collaboration (2019). All-sky measurement of the anisotropy of cosmic rays at 10 TeV and mapping of the local interstellar magnetic field. Astrophys. J..

[CR7] Accardo L., Aguilar M., Aisa D., Alvino A., Ambrosi G., Andeen K., Arruda L., Attig N., Azzarello P., Bachlechner A. (2014). High statistics measurement of the positron fraction in primary cosmic rays of 0.5–500 GeV with the Alpha Magnetic Spectrometer on the International Space Station. Phys. Rev. Lett..

[CR8] Adriani O., the Calet Collaboration (2018). Extended measurement of the cosmic-ray electron and positron spectrum from 11 GeV to 4.8 TeV with the Calorimetric Electron Telescope on the International Space Station. Phys. Rev. Lett..

[CR9] Adriani O., Barbarino G.C., Bazilevskaya G.A., Bellotti R., Boezio M., Bogomolov E.A., Bonechi L., Bongi M., Bonvicini V., Bottai S., Bruno A., Cafagna F., Campana D., Carlson P., Casolino M., Castellini G., de Pascale M.P., de Rosa G., de Simone N., di Felice V., Galper A.M., Grishantseva L., Hofverberg P., Koldashov S.V., Krutkov S.Y., Kvashnin A.N., Leonov A., Malvezzi V., Marcelli L., Menn W., Mikhailov V.V., Mocchiutti E., Orsi S., Osteria G., Papini P., Pearce M., Picozza P., Ricci M., Ricciarini S.B., Simon M., Sparvoli R., Spillantini P., Stozhkov Y.I., Vacchi A., Vannuccini E., Vasilyev G., Voronov S.A., Yurkin Y.T., Zampa G., Zampa N., Zverev V.G. (2009). An anomalous positron abundance in cosmic rays with energies 1.5–100 GeV. Nature.

[CR10] Adriani O., Barbarino G.C., Bazilevskaya G.A., Bellotti R., Boezio M., Bogomolov E.A., Bongi M., Bonvicini V., Bottai S., Bruno A., Cafagna F., Campana D., Carlson P., Casolino M., Castellini G., De Santis C., Di Felice V., Galper A.M., Karelin A.V., Koldashov S.V., Koldobskiy S.A., Krutkov S.Y., Kvashnin A.N., Leonov A., Malakhov V., Marcelli L., Martucci M., Mayorov A.G., Menn W., Mergé M., Mikhailov V.V., Mocchiutti E., Monaco A., Mori N., Munini R., Osteria G., Panico B., Papini P., Pearce M., Picozza P., Ricci M., Ricciarini S.B., Simon M., Sparvoli R., Spillantini P., Stozhkov Y.I., Vacchi A., Vannuccini E., Vasilyev G.I., Voronov S.A., Yurkin Y.T., Zampa G., Zampa N., Potgieter M.S., Vos E.E. (2016). Time dependence of the electron and positron components of the cosmic radiation measured by the PAMELA experiment between July 2006 and December 2015. Phys. Rev. Lett..

[CR11] Adriani O., Barbarino G.C., Bazilevskaya G.A., Bellotti R., Boezio M., Bogomolov E.A., Bongi M., Bonvicini V., Bottai S., Bruno A., Cafagna F., Campana D., Carlson P., Casolino M., Castellini G., De Santis C., Di Felice V., Galper A.M., Karelin A.V., Koldashov S.V., Koldobskiy S., Krutkov S.Y., Kvashnin A.N., Leonov A., Malakhov V., Marcelli L., Martucci M., Mayorov A.G., Menn W., Mergè M., Mikhailov V.V., Mocchiutti E., Monaco A., Munini R., Mori N., Osteria G., Panico B., Papini P., Pearce M., Picozza P., Ricci M., Ricciarini S.B., Simon M., Sparvoli R., Spillantini P., Stozhkov Y.I., Vacchi A., Vannuccini E., Vasilyev G., Voronov S.A., Yurkin Y.T., Zampa G., Zampa N. (2017). Ten years of PAMELA in space. Riv. Nuovo Cimento.

[CR12] Aguilar M., AMS Collaboration (2018). Observation of fine time structures in the cosmic proton and helium fluxes with the Alpha Magnetic Spectrometer on the International Space Station. Phys. Rev. Lett..

[CR13] Aguilar M., the AMS Collaboration (2018). Observation of complex time structures in the cosmic-ray electron and positron fluxes with the Alpha Magnetic Spectrometer on the International Space Station. Phys. Rev. Lett..

[CR14] Aguilar M., the AMS Collaboration (2021). Periodicities in the daily proton fluxes from 2011 to 2019 measured by the Alpha Magnetic Spectrometer on the International Space Station from 1 to 100 GV. Phys. Rev. Lett..

[CR15] Aguilar M., the AMS Collaboration (2021). The Alpha Magnetic Spectrometer (AMS) on the International Space Station: part II — results from the first seven years. Phys. Rep..

[CR16] Aguilar M., Ali Cavasonza L., Ambrosi G., Arruda L., Attig N., Azzarello P., Bachlechner A., Barao F., Barrau A., Barrin L. (2019). Towards understanding the origin of cosmic-ray positrons. Phys. Rev. Lett..

[CR17] Aharonian F., Akhperjanian A.G., Anton G., Barres de Almeida U., Bazer-Bachi A.R., Becherini Y., Behera B., Bernlöhr K., Bochow A., Boisson C., Bolmont J., Borrel V., Brucker J., Brun F., Brun P., Bühler R., Bulik T., Büsching I., Boutelier T., Chadwick P.M., Charbonnier A., Chaves R.C.G., Cheesebrough A., Chounet L.M., Clapson A.C., Coignet G., Dalton M., Daniel M.K., Davids I.D., Degrange B., Deil C., Dickinson H.J., Djannati-Ataï A., Domainko W., O’C Drury L., Dubois F., Dubus G., Dyks J., Dyrda M., Egberts K., Emmanoulopoulos D., Espigat P., Farnier C., Feinstein F., Fiasson A., Förster A., Fontaine G., Füßling M., Gabici S., Gallant Y.A., Gérard L., Gerbig D., Giebels B., Glicenstein J.F., Glück B., Goret P., Göring D., Hauser D., Hauser M., Heinz S., Heinzelmann G., Henri G., Hermann G., Hinton J.A., Hoffmann A., Hofmann W., Holleran M., Hoppe S., Horns D., Jacholkowska A., de Jager O.C., Jahn C., Jung I., Katarzyński K., Katz U., Kaufmann S., Kendziorra E., Kerschhaggl M., Khangulyan D., Khélifi B., Keogh D., Kluźniak W., Kneiske T., Komin N., Kosack K., Kossakowski R., Lamanna G., Lenain J.P., Lohse T., Marandon V., Martin J.M., Martineau-Huynh O., Marcowith A., Masbou J., Maurin D., McComb T.J.L., Medina M.C., Moderski R., Moulin E., Naumann-Godo M., de Naurois M., Nedbal D., Nekrassov D., Nicholas B., Niemiec J., Nolan S.J., Ohm S., Olive J.F., de Oña Wilhelmi E., Orford K.J., Ostrowski M., Panter M., Paz Arribas M., Pedaletti G., Pelletier G., Petrucci P.O., Pita S., Pühlhofer G., Punch M., Quirrenbach A., Raubenheimer B.C., Raue M., Rayner S.M., Reimer O., Renaud M., Rieger F., Ripken J., Rob L., Rosier-Lees S., Rowell G., Rudak B., Rulten C.B., Ruppel J., Sahakian V., Santangelo A., Schlickeiser R., Schöck F.M., Schröder R., Schwanke U., Schwarzburg S., Schwemmer S., Shalchi A., Sikora M., Skilton J.L., Sol H., Spangler D., Stawarz Ł., Steenkamp R., Stegmann C., Stinzing F., Superina G., Szostek A., Tam P.H., Tavernet J.P., Terrier R., Tibolla O., Tluczykont M., van Eldik C., Vasileiadis G., Venter C., Venter L., Vialle J.P., Vincent P., Vivier M., Völk H.J., Volpe F., Wagner S.J., Ward M., Zdziarski A.A., Zech A. (2009). Probing the ATIC peak in the cosmic-ray electron spectrum with H.E.S.S.. Astron. Astrophys..

[CR18] Ahlers M., Mertsch P. (2015). Small-scale anisotropies of cosmic rays from relative diffusion. Astrophys. J. Lett..

[CR19] Alania M.V., Modzelewska R., Wawrzynczak A. (2011). On the relationship of the 27-day variations of the solar wind velocity and galactic cosmic ray intensity in minimum epoch of solar activity. Sol. Phys..

[CR20] Amenomori M., the Tibet ASγ Collaboration (2006). Anisotropy and corotation of galactic cosmic rays. Science.

[CR21] Amenomori M., the Tibet ASγ Collaboration (2010). On temporal variations of the multi-TeV cosmic ray anisotropy using the Tibet III air shower array. Astrophys. J..

[CR22] Aslam O.P.M., Bisschoff D., Potgieter M.S., Boezio M., Munini R. (2019). Modeling of heliospheric modulation of cosmic-ray positrons in a very quiet heliosphere. Astrophys. J..

[CR23] Aslam O.P.M., Bisschoff D., Ngobeni M.D., Potgieter M.S., Munini R., Boezio M., Mikhailov V.V. (2021). Time and charge-sign dependence of the heliospheric modulation of cosmic rays. Astrophys. J..

[CR24] Axford W.I., Dessler A.J., Gottlieb B. (1963). Termination of solar wind and solar magnetic field. Astrophys. J..

[CR25] L.Z. Bao, K. Fang, X.J. Bi, Slow diffusion is necessary to explain the gamma-ray pulsar halos. ArXiv e-prints (2021). 2107.07395

[CR26] Baranov V.B., Malama Y.G. (1993). Model of the solar wind interaction with the local interstellar medium: numerical solution of self-consistent problem. J. Geophys. Res..

[CR27] Baranov V.B., Krasnobaev K.V., Ruderman M.S. (1976). On the model of the solar wind – interstellar medium interaction with two shock waves. Astrophys. Space Sci..

[CR28] Battaner E., Castellano J., Masip M. (2015). Magnetic fields and cosmic-ray anisotropies at TeV energies. Astrophys. J..

[CR29] Bertone G., Hooper D., Silk J. (2005). Particle dark matter: evidence, candidates and constraints. Phys. Rep..

[CR30] Bindi V. (2021). High energy solar energetic particles measured by the Alpha Magnetic Spectrometer (AMS) on the International Space Station. 43rd COSPAR Scientific Assembly. Held 28 January–4 February.

[CR31] Bindi V. (2021). The latest low energy results from the Alpha Magnetic Spectrometer (AMS) on the International Space Station. 43rd COSPAR Scientific Assembly. Held 28 January–4 February.

[CR32] Bindi V. (2021). Time variation of galactic cosmic rays and solar energetic particles measured by the Alpha Magnetic Spectrometer (AMS) on the International Space Station. 43rd COSPAR Scientific Assembly. Held 28 January–4 February.

[CR33] Bîrzan L., Pavlov G.G., Kargaltsev O. (2016). Chandra observations of the elusive pulsar wind nebula around PSR B0656+14. Astrophys. J..

[CR34] Bisschoff D., Potgieter M.S. (2014). Implications of Voyager 1 observations beyond the heliopause for the local interstellar electron spectrum. Astrophys. J..

[CR35] Bisschoff D., Potgieter M.S. (2016). New local interstellar spectra for protons, helium and carbon derived from PAMELA and Voyager 1 observations. Astrophys. Space Sci..

[CR36] Bisschoff D., Potgieter M.S., Aslam O.P.M. (2019). New very local interstellar spectra for electrons, positrons, protons, and light cosmic ray nuclei. Astrophys. J..

[CR37] Bisschoff D., Aslam O.P.M., Ngobeni M.D., Mikhailov V.V., Boezio M., Munini R., Potgieter M.S. (2021). On the very local interstellar spectra for helium, positrons, anti-protons, deuteron and anti-deuteron. Proceedings of the 3rd International Symposium on Cosmic Rays & Astrophysics (ISCRA2021).

[CR38] Blasi P., Amato E. (2011). Positrons from pulsar winds. Astrophys. Space Sci. Proc..

[CR39] Boezio M., Munini R., Adriani O., Barbarino G.C., Bazilevskaya G.A., Bellotti R., Bogomolov E.A., Bongi M., Bonvicini G., Bottai S., Bruno A., Cafagna F., Campana D., Carlson P., Casolino M., Castellini G., De Santis C., Di Felice V., Galper A.M., Karelin A.V., Koldashov S.V., Koldobskiy S., Krutkov S.Y., Kvashnin A.N., Leonov A., Malakhov V., Marcelli L., Martucci M., Mayorov A.G., Menn W., Merge’ M., Mikhailov V.V., Mocchiutti E., Monaco A., Mori N., Osteria G., Panico B., Papini P., Pearce M., Picozza P., Ricci M., Ricciarini S.B., Simon M., Sparvoli R., Spillantini P., Stozhkov Y.I., Vacchi A., Vannuccini E., Vasilyev G., Voronov S.A., Yurkin Y.T., Zampa G., Zampa N. (2017). The PAMELA experiment: a cosmic ray experiment deep inside the heliosphere. 35th International Cosmic Ray Conference (ICRC2017).

[CR40] Boschini M.J., Torre S.D., Gervasi M., Grandi D., Johannesson G., Kachelriess M., Vacca G.L., Masi N., Moskalenko I.V., Orlando E., Ostapchenko S.S., Porter S.P.T.A., Quadrani L., Rancoita P.G. (2017). Solution of heliospheric propagation: unveiling the local interstellar spectra of cosmic ray species. Astrophys. J..

[CR41] Boschini M.J., Torre S.D., Gervasi M., Grandi D., Johannesson G., Vacca G.L., Masi N., Moskalenko I.V., Pensotti S., Porter T.A., Quadrani L., Rancoita P.G., Rozza D., Tacconi M. (2018). Helmod in the works: from direct observations to the local interstellar spectrum of cosmic-ray electrons. Astrophys. J..

[CR42] Boschini M.J., Torre S.D., Gervasi M., Grandi D., Jóhannesson G., Vacca G.L., Masi N., Moskalenko I.V., Pensotti S., Porter T.A., Quadrani L., Rancoita P.G., Rozza D., Tacconi M. (2018). Deciphering the local interstellar spectra of primary cosmic-ray species with HelMod. Astrophys. J..

[CR43] Boschini M.J., Della Torre S., Gervasi M., La Vacca G., Rancoita P.G. (2019). The HELMOD model in the works for inner and outer heliosphere: from AMS to Voyager probes observations. Adv. Space Res..

[CR44] Boschini M.J., Della Torre S., Gervasi M., Grandi D., Jøhannesson G., La Vacca G., Masi N., Moskalenko I.V., Pensotti S., Porter T.A., Quadrani L., Rancoita P.G., Rozza D., Tacconi M. (2020). Deciphering the local interstellar spectra of secondary nuclei with the Galprop/Helmod framework and a hint for primary lithium in cosmic rays. Astrophys. J..

[CR45] Boschini M.J., Torre S.D., Gervasi M., Grandi D., Johannesson G., Vacca G.L., Masi N., Moskalenko I.V., Pensotti S., Porter T.A., Quadrani L., Rancoita P.G., Rozza D., Tacconi M. (2020). Inference of the local interstellar spectra of cosmic-ray nuclei $z<28$ with the GalProp-HelMod framework. Astrophys. J. Suppl. Ser..

[CR46] Boschini M.J. (2020). Deciphering the local interstellar spectra of secondary nuclei with the Galprop/Helmod framework and a hint for primary lithium in cosmic rays. Astrophys. J..

[CR47] Boschini M.J., Torre S.D., Gervasi M., Grandi D., Johannesson G., Vacca G.L., Masi N., Moskalenko I.V., Pensotti S., Porter T.A., Quadrani L., Rancoita P.G., Rozza D., Tacconi M. (2021). The discovery of a low-energy excess in cosmic-ray iron: evidence of the past supernova activity in the local bubble. Astrophys. J..

[CR48] Boschini M.J., Della Torre S., Gervasi M., Grandi D., Jóhannesson G., La Vacca G., Masi N., Moskalenko I.V., Pensotti S., Porter T.A., Quadrani L., Rancoita P.G., Rozza D., Tacconi M. (2022). A hint of a low-energy excess in cosmic-ray fluorine. Astrophys. J..

[CR49] P. Brandt, E. Provornikova, S. Bale, A. Cocoros, R. DeMajistre, K. Dialynas, S. Eriksson, B. Fields, A. Galli, M. Hill, M. Horanyi, T. Horbury, P. Kollmann, J. Kinnison, G. Fountain, S. Krimigis, W. Kurth, J. Linsky, C. Lisse, K. Mandt, W. Magnes, R. McNutt, J. Miller, E. Moebius, P. Mostafavi, M. Opher, L. Paxton, F. Plaschke, A. Poppe, E. Roelof, K. Runyon, S. Redfield, N. Schwadron, V. Sterken, P. Swaczyna, J. Szalay, D. Turner, H. Vannier, R. Wimmer-Schweingruber, P. Wurz, E. Zirnstein, Future exploration of the outer heliosphere and very local interstellar medium by interstellar probe. Space Sci. Rev. **218** (2022) 10.1007/s11214-022-00943-x PMC9974711 36874191

[CR50] Brownsberger S., Romani R.W. (2014). A survey for $\text{H}\alpha$ pulsar bow shocks. Astrophys. J..

[CR51] Burger J.J., Swanenburg B.N. (1973). Energy dependent time lag in the long-term modulation of cosmic rays. J. Geophys. Res..

[CR52] Burlaga L.F., Ness N.F. (2016). Observations of the interstellar magnetic field in the outer heliosheath: Voyager 1. Astrophys. J..

[CR53] Burlaga L.F., McDonald F.B., Ness N.F., Schwenn R., Lazarus A.J., Mariani F. (1984). Interplanetary flow systems associated with cosmic ray modulation in 1977–1980. J. Geophys. Res..

[CR54] Burlaga L.F., McDonald F.B., Goldstein M.L., Lazarus A.J. (1985). Cosmic ray modulation and turbulent interaction regions near 11 AU. J. Geophys. Res..

[CR55] Burlaga L.F., Wang C., Richardson J.D., Ness N.F. (2003). Evolution of magnetic fields in corotating interaction regions from 1 to 95 AU: order to chaos. Astrophys. J..

[CR56] Burlaga L.F., Ness N.F., Acũna M.H. (2006). Multiscale structure of magnetic fields in the heliosheath. J. Geophys. Res. Space Phys..

[CR57] Burlaga L.F., Ness N.F., Gurnett D.A., Kurth W.S. (2013). Evidence for a shock in interstellar plasma: Voyager 1. Astrophys. J. Lett..

[CR58] Burlaga L.F., Florinski V., Ness N.F. (2015). In situ observations of magnetic turbulence in the local interstellar medium. Astrophys. J. Lett..

[CR59] Burlaga L.F., Florinski V., Ness N.F. (2018). Turbulence in the outer heliosheath. Astrophys. J..

[CR60] Burlaga L.F., Ness N.F., Richardson J.D. (2018). Heliosheath magnetic field and plasma observed by Voyager 2 during 2015 near solar maximum. Astrophys. J..

[CR61] Burlaga L.F., Ness N.F., Berdichevsky D.B., Jian L.K., Park J., Mostafavi P., Richardson J.D. (2019). A magnetic pressure front upstream of the heliopause and the heliosheath magnetic fields and plasma, observed during 2017. Astrophys. J..

[CR62] Burlaga L.F., Ness N.F., Berdichevsky D.B., Park J., Jian L.K., Szabo A., Stone E.C., Richardson J.D. (2019). Magnetic field and particle measurements made by Voyager 2 at and near the heliopause. Nat. Astron..

[CR63] Burlaga L.F., Berdichevsky D.B., Jian L.K., Koval A., Ness N.F., Park J., Richardson J.D., Szabo A. (2021). Magnetic fields observed by Voyager 2 in the heliosheath. Astrophys. J..

[CR64] Burlaga L.F., Kurth W.S., Gurnett D.A., Berdichevsky D.B., Jian L.K., Ness N.F., Park J., Szabo A. (2021). Magnetic field and plasma density observations of a pressure front by Voyager 1 during 2020 in the very local interstellar medium. Astrophys. J..

[CR65] Bykov A.M., Toptygin I.N. (1987). Effect of shocks on interstellar turbulence and cosmic-ray dynamics. Astrophys. Space Sci..

[CR66] Bykov A.M., Amato E., Petrov A.E., Krassilchtchikov A.M., Levenfish K.P. (2017). Pulsar wind nebulae with bow shocks: non-thermal radiation and cosmic ray leptons. Space Sci. Rev..

[CR67] Bykov A.M., Petrov A.E., Krassilchtchikov A.M., Levenfish K.P., Osipov S.M., Pavlov G.G. (2019). GeV-TeV cosmic-ray leptons in the solar system from the bow shock wind nebula of the nearest millisecond pulsar J0437-4715. Astrophys. J. Lett..

[CR68] Bykov A.M., Marcowith A., Amato E., Kalyashova M.E., Kruijssen J.M.D., Waxman E. (2020). High-energy particles and radiation in star-forming regions. Space Sci. Rev..

[CR69] Cholis I., Hooper D., Linden T. (2016). A predictive analytic model for the solar modulation of cosmic rays. Phys. Rev. D.

[CR70] Cholis I., Linden T., Hooper D. (2019). A robust excess in the cosmic-ray antiproton spectrum: implications for annihilating dark matter. Phys. Rev. D.

[CR71] Christon S.P., Cummings A.C., Stone E.C., Behannon K.W., Burlaga L.F., Jokipii J.R., Kota J. (1986). Differential measurement and model calculations of cosmic ray latitudinal gradient with respect to the heliospheric current sheet. J. Geophys. Res..

[CR72] Clem J.M., Clements D.P., Esposito J., Evenson P., Huber D., L’Heureux J., Meyer P., Constantin C. (1996). Solar modulation of cosmic electrons. Astrophys. J..

[CR73] Clem J.M., Evenson P., Huber D., Pyle R., Lopate C., Simpson J.A. (2000). Charge sign dependence of cosmic ray modulation near a rigidity of 1 GV. J. Geophys. Res..

[CR74] Clem J., Evenson P., Heber B. (2002). Cosmic electron gradients in the inner heliosphere. Geophys. Res. Lett..

[CR75] Corti C., Bindi V., Consolandi C., Whitman K. (2016). Solar modulation of the local interstellar spectrum with Voyager 1, AMS-02, PAMELA, and BESS. Astrophys. J..

[CR76] Corti C., Potgieter M.S., Bindi V., Consolandi C., Light C., Palermo M., Popkow A. (2019). Numerical modeling of galactic cosmic-ray proton and helium observed by AMS-02 during the solar maximum of solar cycle 24. Astrophys. J..

[CR77] Corti C., Potgieter M.S., Bindi V., Consolandi C., Light C., Palermo M., Popkow A. (2019). Numerical modeling of galactic cosmic-ray proton and helium observed by AMS-02 during the solar maximum of solar cycle 24. Astrophys. J..

[CR78] Crooker N.U., Gosling J.T., Bothmer V., Forsyth R.J., Gazis P.R., Hewish A., Horbury T.S., Intriligator D.S., Jokipii J.R., Kóta J. (1999). Cir morphology, turbulence, discontinuities, and energetic particles. Space Sci. Rev..

[CR79] Cui M.Y., Pan X., Yuan Q., Fan Y.Z., Zong H.S. (2018). Revisit of cosmic ray antiprotons from dark matter annihilation with updated constraints on the background model from AMS-02 and collider data. J. Cosmol. Astropart. Phys..

[CR80] Cummings A.C., Stone E.C., Webber W.R. (1987). Latitudinal and radial gradients of anomalous and galactic cosmic rays in the outer heliosphere. Geophys. Res. Lett..

[CR81] Cummings A.C., Stone E.C., Heikkila B.C., Lal N., Webber W.R., Jóhannesson G., Moskalenko I.V., Orlando E., Porter T.A. (2016). Galactic cosmic rays in the local interstellar medium: Voyager 1 observations and model results. Astrophys. J..

[CR82] Cuoco A., Heisig J., Korsmeier M., Krämer M. (2018). Constraining heavy dark matter with cosmic-ray antiprotons. J. Cosmol. Astropart. Phys..

[CR83] DAMPE Collaboration (2017). Direct detection of a break in the teraelectronvolt cosmic-ray spectrum of electrons and positrons. Nature.

[CR84] de Simone N., di Felice V., Gieseler J., Boezio M., Casolino M., Picozza P., Heber B., PAMELA Collaboration (2011). Latitudinal and radial gradients of galactic cosmic ray protons in the inner heliosphere – PAMELA and Ulysses observations. Astrophys. Space Sci. Trans..

[CR85] Della Torre S., Gervasi M., Rancoita P.G., Rozza D., Treves A. (2015). Pulsar wind nebulae as a source of the observed electron and positron excess at high energy: the case of Vela-X. J. High Energy Astrophys..

[CR86] Desiati P., Lazarian A. (2013). Anisotropy of TeV cosmic rays and outer heliospheric boundaries. Astrophys. J..

[CR87] Di Felice V., Munini R., Vos E.E., Potgieter M.S. (2017). New evidence for charge-sign-dependent modulation during the solar minimum of 2006 to 2009. Astrophys. J..

[CR88] Di Mauro M., Manconi S., Donato F. (2019). Detection of a $\gamma$-ray halo around Geminga with the Fermi-LAT data and implications for the positron flux. Phys. Rev. D.

[CR89] Di Sciascio G., ARGO-YBJ Collaboration (2012). Gamma-ray astronomy with ARGO-YBJ. Mem. Soc. Astron. Ital..

[CR90] Dialynas K., Galli A., Dayeh M.A., Cummings A.C., Decker R.B., Fuselier S.A., Gkioulidou M., Roussos E., Krimigis S.M., Mitchell D.G., Richardson J.D., Opher M. (2020). Combined $\sim10~\text{eV}$ to $\sim344~\text{MeV}$ particle spectra and pressures in the heliosheath along the Voyager 2 trajectory. Astrophys. J. Lett..

[CR91] Donnini F. (2021). Precision measurement of the monthly boron, carbon and oxygen fluxes in cosmic rays with the Alpha Magnetic Spectrometer on the International Space Station. 43rd COSPAR Scientific Assembly. Held 28 January–4 February.

[CR92] Duggal S.P., Tsurutani B.T., Pomerantz M.A., Tsao C.H., Smith E.J. (1981). Relativistic cosmic rays and corotating interaction regions. J. Geophys. Res..

[CR93] El-Borie M.A. (2001). Cosmic-ray intensities near the heliospheric current sheet throughout three solar activity cycles. J. Phys. G, Nucl. Part. Phys..

[CR94] Engelmann J.J., Ferrando P., Soutoul A., Goret P., Juliusson E., Koch-Miramond L., Lund N., Masse P., Peters B., Petrou N., Rasmussen I.L. (1990). Charge composition and energy spectra of cosmic-ray nuclei for elements from Be to Ni – results from HEAO-3-C2. Astron. Astrophys..

[CR95] Evenson P. (1998). Cosmic ray electrons. Space Sci. Rev..

[CR96] Evenson P., Garcia-Munoz M., Meyer P., Pyle K.R., Simpson J.A. (1983). A quantitative test of solar modulation theory – the proton, helium, and electron spectra from 1965 through 1979. Astrophys. J. Lett..

[CR97] Evoli C., Amato E., Blasi P., Aloisio R. (2021). Galactic factories of cosmic-ray electrons and positrons. Phys. Rev. D.

[CR98] Fahr H.J., Heyl M. (2020). Probing the thermodynamic conditions of the heliosheath plasma by shock wave propagation. Astron. Astrophys..

[CR99] Fahr H.J., Heyl M. (2020). Suprathermal plasma distribution functions with relativistic cut-offs. Mon. Not. R. Astron. Soc..

[CR100] Fang K., Bi X.J., Yin P.F. (2019). Reanalysis of the pulsar scenario to explain the cosmic positron excess considering the recent developments. Astrophys. J..

[CR101] Fang K., Bi X.J., Yin P.F. (2020). DAMPE proton spectrum indicates a slow-diffusion zone in the nearby ISM. Astrophys. J..

[CR102] Ferrando P., Raviart A., Haasbroek L.J., Potgieter M.S., Droege W., Heber B., Kunow H., Mueller-Mellin R., Sierks H., Wibberenz G., Paizis C. (1996). Latitude variations of $\sim7~\text{MeV}$ and $>300~\text{MeV}$ cosmic ray electron fluxes in the heliosphere: ULYSSES COSPIN/KET results and implications. Astron. Astrophys..

[CR103] Ferreira S.E.S., Potgieter M.S. (2004). Long-term cosmic-ray modulation in the heliosphere. Astrophys. J..

[CR104] Ferreira S.E.S., Potgieter M.S., Webber W.R. (2004). Modulation of low-energy cosmic ray electrons in the outer heliosphere. Adv. Space Res..

[CR105] Florinski V., Pogorelov N.V. (2009). Four-dimensional transport of galactic cosmic rays in the outer heliosphere and heliosheath. Astrophys. J..

[CR106] Forbush S.E. (1937). On the effects in cosmic-ray intensity observed during the recent magnetic storm. Phys. Rev..

[CR107] Fraternale F., Pogorelov N.V. (2021). Waves and turbulence in the very local interstellar medium: from macroscales to microscales. Astrophys. J..

[CR108] Fraternale F., Pogorelov N.V., Richardson J.D., Tordella D. (2019). Magnetic turbulence spectra and intermittency in the heliosheath and in the local interstellar medium. Astrophys. J..

[CR109] Freir P.S., Waddington C.J. (1965). Electron, hydrogen nuclei, and helium nuclei observed in primary cosmic radiation during 1963. J. Geophys. Res..

[CR110] Fujii Z., McDonald F.B. (1997). Radial intensity gradients of galactic cosmic rays (1972–1995) in the heliosphere. J. Geophys. Res. Space Phys..

[CR111] Garcia-Munoz M., Meyer P., Pyle K.R., Simpson J.A., Evenson P. (1986). The dependence of solar modulation on the sign of the cosmic ray particle charge. J. Geophys. Res..

[CR112] Gazis P.R. (2000). A large-scale survey of corotating interaction regions and their successors in the outer heliosphere. J. Geophys. Res..

[CR113] Ghanbari K., Florinski V., Guo X., Hu Q., Leske R.A. (2019). Galactic cosmic rays modulation in the vicinity of corotating interaction regions: observations during the last two solar minima. Astrophys. J..

[CR114] Ghelfi A., Barao F., Derome L., Maurin D. (2016). Non-parametric determination of H and He interstellar fluxes from cosmic-ray data. Astron. Astrophys..

[CR115] Giacalone J., Jokipii J.R. (2015). A new model for the heliosphere’s “IBEX ribbon”. Astrophys. J. Lett..

[CR116] Giacinti G., Kirk J.G. (2019). TeV-PeV cosmic-ray anisotropy and local interstellar turbulence. J. Phys. Conf. Ser..

[CR117] Giacinti G., Sigl G. (2012). Local magnetic turbulence and TeV-PeV cosmic ray anisotropies. Phys. Rev. Lett..

[CR118] Gieseler J., Heber B. (2016). Spatial gradients of GCR protons in the inner heliosphere derived from Ulysses COSPIN/KET and PAMELA measurements. Astron. Astrophys..

[CR119] Gil A., Alania M.V. (2013). Theoretical and experimental studies of the rigidity spectrum of the 27-day variation of the galactic cosmic ray intensity in different epochs of solar activity. Sol. Phys..

[CR120] Gleeson L.J., Axford W.I. (1968). The Compton-Getting effect. Astrophys. Space Sci..

[CR121] Gosling J.T., Pizzo V.J. (1999). Formation and evolution of corotating interaction regions and their three dimensional structure. Space Sci. Rev..

[CR122] Guillian G. (2007). Observation of the anisotropy of 10 TeV primary cosmic ray nuclei flux with the Super-Kamiokande-I detector. Phys. Rev. D.

[CR123] Guo X., Florinski V. (2014). Corotating interaction regions and the 27 day variation of galactic cosmic rays intensity at 1 AU during the cycle 23/24 solar minimum. J. Geophys. Res..

[CR124] Guo X., Florinski V. (2014). Galactic cosmic-ray modulation near the heliopause. Astrophys. J..

[CR125] Guo X., Florinski V. (2016). Galactic cosmic-ray intensity modulation by corotating interaction region stream interfaces at 1 AU. Astrophys. J..

[CR126] Guo X., Florinski V., Wang C., Ghanbari K. (2021). Superposed epoch analysis of galactic cosmic rays and solar wind based on ace observations during two recent solar minima. Astrophys. J..

[CR127] Guo X., Zhou Y., Wang C., Liu Y.D. (2021). Propagation of large-scale solar wind events in the outer heliosphere from a numerical MHD simulation. Earth Planet. Phys..

[CR128] Gurnett D.A., Kurth W.S., Allendorf S.C., Poynter R.L. (1993). Radio emission from the heliopause triggered by an interplanetary shock. Science.

[CR129] Gurnett D.A., Kurth W.S., Burlaga L.F., Ness N.F. (2013). In situ observations of interstellar plasma with Voyager 1. Science.

[CR130] Gurnett D.A., Kurth W.S., Stone E.C., Cummings A.C., Krimigis S.M., Decker R.B., Ness N.F., Burlaga L.F. (2015). Precursors to interstellar shocks of solar origin. Astrophys. J..

[CR131] Gurnett D.A., Kurth W.S., Stone E.C., Cummings A.C., Heikkila B., Lal N., Krimigis S.M., Decker R.B., Ness N.F., Burlaga L.F. (2021). A foreshock model for interstellar shocks of solar origin: Voyager 1 and 2 observations. Astron. J..

[CR132] Heber B., Potgieter M.S. (2006). Cosmic rays at high heliolatitudes. Space Sci. Rev..

[CR133] Heber B., Droege W., Ferrando P., Haasbroek L.J., Kunow H., Mueller-Mellin R., Paizis C., Potgieter M.S., Raviart A., Wibberenz G. (1996). Spatial variation of $>40~\text{MeV/n}$ nuclei fluxes observed during the ULYSSES rapid latitude scan. Astron. Astrophys..

[CR134] Heber B., Dröge W., Kunow H., Müller-Mellin R., Wibberenz G., Ferrando P., Raviart A., Paizis C. (1996). Spatial variation of $>106~\text{Mev}$ proton fluxes observed during the Ulysses rapid latitude scan: Ulysses COSPIN/KET results. Geophys. Res. Lett..

[CR135] Heber B., Gieseler J., Dunzlaff P., Gómez-Herrero R., Klassen A., Müller-Mellin R., Mewaldt R.A., Potgieter M.S., Ferreira S.E.S. (2008). Latitudinal gradients of galactic cosmic rays during the 2007 solar minimum. Astrophys. J..

[CR136] Heber B., Kopp A., Gieseler J., Müller-Mellin R., Fichtner H., Scherer K., Potgieter M.S., Ferreira S.E.S. (2009). Modulation of galactic cosmic ray protons and electrons during an unusual solar minimum. Astrophys. J..

[CR137] Herbst K., Baalmann L., Bykov A., Engelbrecht N., Ferreira S., Izmodenov V., Korolkov S., Levenfish K., Linsky J., Meyer D.A., Scherer K., Strauss R. (2022). Astrospheres of planet-hosting cool stars and beyond – when modeling meets observations. Space Sci. Rev..

[CR138] Hill M.E., Allen R.C., Kollmann P., Brown L.E., Decker R.B., McNutt R.L., Krimigis S.M., Andrews G.B., Bagenal F., Clark G., Elliott H.A., Jaskulek S.E., Kusterer M.B., Leske R.A., Lisse C.M., Mewaldt R.A., Nelson K.S., Richardson J.D., Romeo G., Salazar N.A., Vandegriff J.D., Bernardoni E.A., Gladstone G.R., Horanyi M., Linscott I.R., Singer K.N., Steffl A.J., Summers M.E., Throop H.B., Young L.A., Olkin C.B., Parker J.W., Spencer J.R., Stern S.A., Verbiscer A.J., Weaver H.A. (2020). Influence of solar disturbances on galactic cosmic rays in the solar wind, heliosheath, and local interstellar medium: Advanced Composition Explorer, New Horizons, and Voyager observations. Astrophys. J..

[CR139] Hoeksema J.T. (1995). The large-scale structure of the heliospheric current sheet during the ULYSSES epoch. Space Sci. Rev..

[CR140] Hooper D., Blasi P., Serpico P.D. (2009). Pulsars as the sources of high energy cosmic ray positrons. J. Cosmol. Astropart. Phys..

[CR141] Indriolo N., Neufeld D.A., Gerin M., Schilke P., Benz A.O., Winkel B., Menten K.M., Chambers E.T., Black J.H., Bruderer S., Falgarone E., Godard B., Goicoechea J.R., Gupta H., Lis D.C., Ossenkopf V., Persson C.M., Sonnentrucker P., van der Tak F.F.S., van Dishoeck E.F., Wolfire M.G., Wyrowski F. (2015). Herschel survey of galactic $\text{OH}^{+}$, $\text{H}_{2}\text{O}^{+}$, and $\text{H}_{3}\text{O}^{+}$: probing the molecular hydrogen fraction and cosmic-ray ionization rate. Astrophys. J..

[CR142] Intriligator D.S., Jokipii J.R., Horbury T.S., Intriligator J.M., Forsyth R.J., Kunow H., Wibberenz G., Gosling J.T. (2001). Processes associated with particle transport in corotating interaction regions and near stream interfaces. J. Geophys. Res..

[CR143] Jokipii J.R., Scherer K., Fichtner H., Fahr H.J., Marsch E. (2001). Cosmic rays in the outer heliosphere and nearby interstellar medium. The Outer Heliosphere: The Next Frontiers.

[CR144] Jokipii J.R., Kóta J. (2014). Interpretation of the disturbance in galactic cosmic rays observed on Voyager 1 beyond the heliopause. Astrophys. J. Lett..

[CR145] Jokipii J.R., Levy E.H., Hubbard W.B. (1977). Effects of particle drift on cosmic-ray transport. I. General properties, application to solar modulation. Astrophys. J..

[CR146] Kim T.K., Pogorelov N.V., Burlaga L.F. (2017). Modeling shocks detected by Voyager 1 in the local interstellar medium. Astrophys. J. Lett..

[CR147] Kleimann J., Dialynas K., Fraternale F., Galli A., Heerikhuisen J., Izmodenov V., Kornbleuth M., Opher M., Pogorelov N. (2022). The structure of the large-scale heliosphere as seen by current models. Space Sci. Rev..

[CR148] Kóta J., Jokipii J.R. (1991). The role of corotating interaction regions in cosmic-ray modulation. Geophys. Res. Lett..

[CR149] Kóta J., Jokipii J.R. (2014). Are cosmic rays modulated beyond the heliopause?. Astrophys. J..

[CR150] Kóta J., Jokipii J.R. (2017). Transient cosmic-ray events beyond the heliopause: interpreting Voyager-1 observations. Astrophys. J..

[CR151] Krainev M., Kalinin M., Aslam O.P.M., Ngobeni D., Potgieter M. (2021). On the dependence of maximum GCR intensity on heliospheric factors for the last five sunspot minima. Adv. Space Res..

[CR152] Krimigis S.M., Decker R.B., Roelof E.C., Hill M.E., Armstrong T.P., Gloeckler G., Hamilton D.C., Lanzerotti L.J. (2013). Search for the exit: Voyager 1 at heliosphere’s border with the galaxy. Science.

[CR153] Krimigis S.M., Decker R.B., Roelof E.C., Hill M.E., Bostrom C.O., Dialynas K., Gloeckler G., Hamilton D.C., Keath E.P., Lanzerotti L.J. (2019). Energetic charged particle measurements from Voyager 2 at the heliopause and beyond. Nat. Astron..

[CR154] Le Roux J.A., Potgieter M.S. (1995). The simulation of complete 11 and 22 year modulation cycles for cosmic rays in the heliosphere using a drift model with global merged interaction regions. Astrophys. J..

[CR155] Leske R.A., Cummings A.C., Mewaldt R.A., Stone E.C. (2013). Anomalous and galactic cosmic rays at 1 AU during the cycle 23/24 solar minimum. Space Sci. Rev..

[CR156] Linsky J., Redfield S., Ryder D., Moebius E. (2022). Inhomogeneity in the local ISM and its relation to the heliosphere. Space Sci. Rev..

[CR157] Lipari P. (2018). Spectral features in the cosmic ray fluxes. Astropart. Phys..

[CR158] López-Coto R., Parsons R.D., Hinton J.A., Giacinti G. (2018). Undiscovered pulsar in the local bubble as an explanation of the local high energy cosmic ray all-electron spectrum. Phys. Rev. Lett..

[CR159] Luo X., Zhang M., Rassoul H.K., Pogorelov N.V. (2011). Cosmic-ray modulation by the global merged interaction region in the heliosheath. Astrophys. J..

[CR160] Luo X., Zhang M., Rassoul H., Pogorelov N., Heerikhuisen J. (2013). Galactic cosmic-ray modulation in a realistic global magnetohydrodynamic heliosphere. Astrophys. J..

[CR161] Luo X., Zhang M., Potgieter M., Feng X., Pogorelov N.V. (2015). A numerical simulation of cosmic-ray modulation near the heliopause. Astrophys. J..

[CR162] Luo X., Potgieter M.S., Zhang M., Pogorelov N.V., Feng X., Strauss D.T.R. (2016). A numerical simulation of cosmic ray modulation near the heliopause. II. Some physical insights. Astrophys. J..

[CR163] Luo X., Potgieter M.S., Zhang M., Feng X. (2017). A numerical study of Forbush decreases with a 3D cosmic-ray modulation model based on an SDE approach. Astrophys. J..

[CR164] Luo X., Potgieter M.S., Zhang M., Feng X. (2018). A study of electron Forbush decreases with a 3D SDE numerical model. Astrophys. J..

[CR165] Luo X., Potgieter M.S., Bindi V., Zhang M., Feng X. (2019). A numerical study of cosmic proton modulation using AMS-02 observations. Astrophys. J..

[CR166] Luo X., Zhang M., Feng X., Potgieter M.S., Shen F., Bazilevskaya G. (2020). A numerical study of the effects of corotating interaction regions on cosmic-ray transport. Astrophys. J..

[CR167] Marcelli N., Boezio M., Lenni A., Menn W., Munini R., Aslam O.P.M., Bisschoff D., Ngobeni M.D., Potgieter M.S., Adriani O., Barbarino G.C., Bazilevskaya G.A., Bellotti R., Bogomolov E.A., Bongi M., Bonvicini V., Bruno A., Cafagna F., Campana D., Carlson P., Casolino M., Castellini G., De Santis C., Galper A.M., Koldashov S.V., Koldobskiy S., Kvashnin A.N., Leonov A.A., Malakhov V.V., Marcelli L., Martucci M., Mayorov A.G., Mergè M., Mocchiutti E., Monaco A., Mori N., Mikhailov V.V., Osteria G., Panico B., Papini P., Pearce M., Picozza P., Ricci M., Ricciarini S.B., Simon M., Sotgiu A., Sparvoli R., Spillantini P., Stozhkov Y.I., Vacchi A., Vannuccini E., Vasilyev G.I., Voronov S.A., Yurkin Y.T., Zampa G., Zampa N. (2020). Time dependence of the flux of helium nuclei in cosmic rays measured by the PAMELA experiment between 2006 July and 2009 December. Astrophys. J..

[CR168] Marcelli N., Boezio M., Lenni A., Menn W., Munini R., Aslam O.P.M., Bisschoff D., Ngobeni M.D., Potgieter M.S., Adriani O., Barbarino G.C., Bazilevskaya G.A., Bellotti R., Bogomolov E.A., Bongi M., Bonvicini V., Bruno A., Cafagna F., Campana D., Carlson P., Casolino M., Castellini G., De Santis C., Galper A.M., Koldashov S.V., Koldobskiy S., Kvashnin A.N., Leonov A.A., Malakhov V.V., Marcelli L., Martucci M., Mayorov A.G., Mergè M., Mocchiutti E., Monaco A., Mori N., Mikhailov V.V., Osteria G., Panico B., Papini P., Pearce M., Picozza P., Ricci M., Ricciarini S.B., Simon M., Sotgiu A., Sparvoli R., Spillantini P., Stozhkov Y.I., Vacchi A., Vannuccini E., Vasilyev G.I., Voronov S.A., Yurkin Y.T., Zampa G., Zampa N. (2022). Helium fluxes measured by the PAMELA experiment from the minimum to the maximum solar activity for solar cycle 24. Astrophys. J. Lett..

[CR169] Martucci M., Munini R., Boezio M., Di Felice V., Adriani O., Barbarino G.C., Bazilevskaya G.A., Bellotti R., Bongi M., Bonvicini V., Bottai S., Bruno A., Cafagna F., Campana D., Carlson P., Casolino M., Castellini G., De Santis C., Galper A.M., Karelin A.V., Koldashov S.V., Koldobskiy S., Krutkov S.Y., Kvashnin A.N., Leonov A., Malakhov V., Marcelli L., Marcelli N., Mayorov A.G., Menn W., Mergè M., Mikhailov V.V., Mocchiutti E., Monaco A., Mori N., Osteria G., Panico B., Papini P., Pearce M., Picozza P., Ricci M., Ricciarini S.B., Simon M., Sparvoli R., Spillantini P., Stozhkov Y.I., Vacchi A., Vannuccini E., Vasilyev G., Voronov S.A., Yurkin Y.T., Zampa G., Zampa N., Potgieter M.S., Raath J.L. (2018). Proton fluxes measured by the PAMELA experiment from the minimum to the maximum solar activity for solar cycle 24. Astrophys. J. Lett..

[CR170] McComas D.J., Allegrini F., Bochsler P., Bzowski M., Christian E.R., Crew G.B., DeMajistre R., Fahr H., Fichtner H., Frisch P.C., Funsten H.O., Fuselier S.A., Gloeckler G., Gruntman M., Heerikhuisen J., Izmodenov V., Janzen P., Knappenberger P., Krimigis S., Kucharek H., Lee M., Livadiotis G., Livi S., MacDowall R.J., Mitchell D., Möbius E., Moore T., Pogorelov N.V., Reisenfeld D., Roelof E., Saul L., Schwadron N.A., Valek P.W., Vanderspek R., Wurz P., Zank G.P. (2009). Global observations of the interstellar interaction from the Interstellar Boundary Explorer (IBEX). Science.

[CR171] McComas D.J., Dayeh M.A., Funsten H.O., Heerikhuisen J., Janzen P.H., Reisenfeld D.B., Schwadron N.A., Szalay J.R., Zirnstein E.J. (2018). Heliosphere responds to a large solar wind intensification: decisive observations from IBEX. Astrophys. J. Lett..

[CR172] McComas D.J., Bzowski M., Dayeh M.A., DeMajistre R., Funsten H.O., Janzen P.H., Kowalska-Leszczyńska I., Kubiak M.A., Schwadron N.A., Sokół J.M., Szalay J.R., Tokumaru M., Zirnstein E.J. (2020). Solar cycle of imaging the global heliosphere: Interstellar Boundary Explorer (IBEX) observations from 2009–2019. Astrophys. J. Suppl. Ser..

[CR173] McDonald F.B., Lal N., McGuire R.E. (1995). The initial cosmic ray recovery phase of solar cycle 22. International Cosmic Ray Conference.

[CR174] McKibben R.B. (1975). Cosmic ray intensity gradients in the solar system. Rev. Geophys. Space Phys..

[CR175] McKibben R.B., Pyle K.R., Simpson J.A. (1979). The solar latitude and radial dependence of the anomalous cosmic-ray helium component. Astrophys. J. Lett..

[CR176] McKibben R.B., Jokipii J.R., Burger R.A., Heber B., Kóta J., McDonald F.B., Paizis C., Potgieter M.S., Richardson I.G. (1999). Modulation of cosmic rays and anomalous components by CIRs. Space Sci. Rev..

[CR177] Mechbal S., Mangeard P.S., Clem J.M., Evenson P.A., Johnson R.P., Lucas B., Roth J. (2020). Measurement of low-energy cosmic-ray electron and positron spectra at 1 au with the AESOP-Lite spectrometer. Astrophys. J..

[CR178] Mewaldt R.A., Davis A.J., Lave K.A., Leske R.A., Stone E.C., Wiedenbeck M.E., Binns W.R., Christian E.R., Cummings A.C., de Nolfo G.A., Israel M.H., Labrador A.W., von Rosenvinge T.T. (2010). Record-setting cosmic-ray intensities in 2009 and 2010. Astrophys. J. Lett..

[CR179] Modzelewska R., Gil A. (2021). Recurrence of galactic cosmic rays intensity and anisotropy in solar minima 23/24 and 24/25 by ACE/CRIS, STEREO, SOHO/EPHIN and neutron monitors. Fourier and wavelet analysis. Dedicated to the memory of Michael Alania. Astron. Astrophys..

[CR180] Modzelewska R., Bazilevskaya G.A., Boezio M., Koldashov S.V., Krainev M.B., Marcelli N., Mayorov A.G., Mayorova M.A., Munini R., Troitskaya I.K., Yulbarisov R.F., Luo X., Potgieter M.S., Aslam O.P.M. (2020). Study of the 27 day variations in GCR fluxes during 2007–2008 based on PAMELA and ARINA observations. Astrophys. J..

[CR181] Mostafavi P., Burlaga L.F., Cairns I.H., Fuselier S.A., Fraternale F., Gurnett D.A., Kim T.K., Kurth W.S., Pogorelov N.V., Provornikova E., Richardson J.D., Turner D.L., Zank G.P. (2022). Shocks in the very local interstellar medium. Space Sci. Rev..

[CR182] Munini R., Boezio M., Bruno A., Christian E.C., de Nolfo G.A., Di Felice V., Martucci M., Merge’ M., Richardson I.G., Ryan J.M., Stochaj S., Adriani O., Barbarino G.C., Bazilevskaya G.A., Bellotti R., Bongi M., Bonvicini V., Bottai S., Cafagna F., Campana D., Carlson P., Casolino M., Castellini G., De Santis C., Galper A.M., Karelin A.V., Koldashov S.V., Koldobskiy S., Krutkov S.Y., Kvashnin A.N., Leonov A., Malakhov V., Marcelli L., Mayorov A.G., Menn W., Mikhailov V.V., Mocchiutti E., Monaco A., Mori N., Osteria G., Panico B., Papini P., Pearce M., Picozza P., Ricci M., Ricciarini S.B., Simon M., Sparvoli R., Spillantini P., Stozhkov Y.I., Vacchi A., Vannuccini E., Vasilyev G., Voronov S.A., Yurkin Y.T., Zampa G., Zampa N., Potgieter M.S. (2018). Evidence of energy and charge sign dependence of the recovery time for the 2006 December Forbush event measured by the PAMELA experiment. Astrophys. J..

[CR183] Ngobeni M.D., Aslam O.P.M., Bisschoff D., Potgieter M.S., Ndiitwani D.C., Boezio M., Marcelli N., Munini R., Mikhailov V.V., Koldobskiy S.A. (2020). The 3D numerical modeling of the solar modulation of galactic protons and helium nuclei related to observations by PAMELA between 2006 and 2009. Astrophys. Space Sci..

[CR184] Nndanganeni R.R., Potgieter M.S. (2018). The global modulation of Galactic and Jovian electrons in the heliosphere. Astrophys. Space Sci..

[CR185] O’c Drury L. (2013). The problem of small angular scale structure in the cosmic ray anisotropy data. International Cosmic Ray Conference.

[CR186] Pamela Collaboration (2017). Ten years of PAMELA in space. Riv. Nuovo Cimento.

[CR187] Parker E.N. (1958). Dynamics of the interplanetary gas and magnetic fields. Astrophys. J..

[CR188] Parker E.N. (1961). The stellar-wind regions. Astrophys. J..

[CR189] Parker E.N. (1965). The passage of energetic charged particles through interplanetary space. Planet. Space Sci..

[CR190] Pauls H.L., Zank G.P. (1996). Interaction of a nonuniform solar wind with the local interstellar medium. J. Geophys. Res..

[CR191] Picozza P., Galper A.M., Castellini G., Adriani O., Altamura F., Ambriola M., Barbarino G.C., Basili A., Bazilevskaja G.A., Bencardino R., Boezio M., Bogomolov E.A., Bonechi L., Bongi M., Bongiorno L., Bonvicini V., Cafagna F., Campana D., Carlson P., Casolino M., de Marzo C., de Pascale M.P., de Rosa G., Fedele D., Hofverberg P., Koldashov S.V., Krutkov S.Y., Kvashnin A.N., Lund J., Lundquist J., Maksumov O., Malvezzi V., Marcelli L., Menn W., Mikhailov V.V., Minori M., Misin S., Mocchiutti E., Morselli A., Nikonov N.N., Orsi S., Osteria G., Papini P., Pearce M., Ricci M., Ricciarini S.B., Runtso M.F., Russo S., Simon M., Sparvoli R., Spillantini P., Stozhkov Y.I., Taddei E., Vacchi A., Vannuccini E., Voronov S.A., Yurkin Y.T., Zampa G., Zampa N., Zverev V.G. (2007). PAMELA a payload for antimatter matter exploration and light-nuclei astrophysics. Astropart. Phys..

[CR192] Pishkalo M.I. (2019). On polar magnetic field reversal in solar cycles 21, 22, 23, and 24. Sol. Phys..

[CR193] Pogorelov N.V., Matsuda T. (1998). Influence of the interstellar magnetic field direction on the shape of the global heliopause. J. Geophys. Res..

[CR194] Pogorelov N.V., Suess S.T., Borovikov S.N., Ebert R.W., McComas D.J., Zank G.P. (2013). Three-dimensional features of the outer heliosphere due to coupling between the interstellar and interplanetary magnetic fields. IV. Solar cycle model based on Ulysses observations. Astrophys. J..

[CR195] Posselt B., Spence G., Pavlov G.G. (2015). A Chandra search for a pulsar wind nebula around PSR B1055-52. Astrophys. J..

[CR196] Posselt B., Pavlov G.G., Slane P.O., Romani R., Bucciantini N., Bykov A.M., Kargaltsev O., Weisskopf M.C., Ng C.Y. (2017). Geminga’s puzzling pulsar wind nebula. Astrophys. J..

[CR197] Potgieter M.S. (2013). Cosmic rays in the inner heliosphere: insights from observations, theory and models. Space Sci. Rev..

[CR198] Potgieter M.S. (2013). Solar modulation of cosmic rays. Living Rev. Sol. Phys..

[CR199] Potgieter M.S. (2014). The charge-sign dependent effect in the solar modulation of cosmic rays. Adv. Space Res..

[CR200] Potgieter M.S. (2017). The global modulation of cosmic rays during a quiet heliosphere: a modeling perspective. Adv. Space Res..

[CR201] Potgieter M.S., Ferreira S.E.S. (2001). Modulation of cosmic rays in the heliosphere over 11 and 22 year cycles: a modelling perspective. Adv. Space Res..

[CR202] Potgieter M.S., Nndanganeni R.R. (2013). A local interstellar spectrum for galactic electrons. Astropart. Phys..

[CR203] Potgieter M.S., Vos E.E. (2017). Difference in the heliospheric modulation of cosmic-ray protons and electrons during the solar minimum period of 2006 to 2009. Astron. Astrophys..

[CR204] Potgieter M.S., Vos E.E., Boezio M., De Simone N., Di Felice V., Formato V. (2014). Modulation of galactic protons in the heliosphere during the unusual solar minimum of 2006 to 2009. Sol. Phys..

[CR205] Potgieter M.S., Vos E.E., Munini R., Boezio M., Di Felice V. (2015). Modulation of galactic electrons in the heliosphere during the unusual solar minimum of 2006–2009: a modeling approach. Astrophys. J..

[CR206] Rangelov B., Pavlov G.G., Kargaltsev O., Durant M., Bykov A.M., Krassilchtchikov A. (2016). First detection of a pulsar bow shock nebula in far-UV: PSR J0437-4715. Astrophys. J..

[CR207] Rankin J.S., McComas D.J., Richardson J.D., Schwadron N.A. (2019). Heliosheath properties measured from a Voyager 2 to Voyager 1 transient. Astrophys. J..

[CR208] Rankin J.S., Stone E.C., Cummings A.C., McComas D.J., Lal N., Heikkila B.C. (2019). Galactic cosmic-ray anisotropies: Voyager 1 in the local interstellar medium. Astrophys. J..

[CR209] Rankin J.S., McComas D.J., Schwadron N.A. (2020). Galactic cosmic-ray anisotropies: electrons observed by Voyager 1 in the very local interstellar medium. Astrophys. J..

[CR210] Recchia S., Gabici S., Aharonian F.A., Vink J. (2019). Local fading accelerator and the origin of TeV cosmic ray electrons. Phys. Rev. D.

[CR211] Recchia S., Di Mauro M., Aharonian F.A., Orusa L., Donato F., Gabici S., Manconi S. (2021). Do the Geminga, Monogem and PSR J0622+3749 $\gamma$-ray halos imply slow diffusion around pulsars?. Phys. Rev. D.

[CR212] Reisenfeld D.B., Bzowski M., Funsten H.O., Heerikhuisen J., Janzen P.H., Kubiak M.A., McComas D.J., Schwadron N.A., Sokół J.M., Zimorino A., Zirnstein E.J. (2021). A three-dimensional map of the heliosphere from IBEX. Astrophys. J. Suppl. Ser..

[CR213] Richardson I.G. (2004). Energetic particles and corotating interaction regions in the solar wind. Space Sci. Rev..

[CR214] Richardson J.D. (2015). Plasma and variability in the heliosheath. J. Phys. Conf. Ser..

[CR215] Richardson I.G. (2018). Solar wind stream interaction regions throughout the heliosphere. Living Rev. Sol. Phys..

[CR216] Richardson I.G., Wibberenz G., Cane H.V. (1996). The relationship between recurring cosmic ray depressions and corotating solar wind streams at $<1~\text{AU}$ IMP 8 and Helios 1 and 2 anticoincidence guard rate observations. J. Geophys. Res..

[CR217] Richardson J.D., Liu Y., Wang C., McComas D.J., Stone E.C., Cummings A.C., Burlaga L.F., Acuna M.H., Ness N.F. (2006). Source and consequences of a large shock near 79 AU. Geophys. Res. Lett..

[CR218] Richardson J.D., Kasper J.C., Wang C., Belcher J.W., Lazarus A.J. (2008). Cool heliosheath plasma and deceleration of the upstream solar wind at the termination shock. Nature.

[CR219] Richardson J.D., Wang C., Liu Y.D., Šafránková J., Němeček Z., Kurth W.S. (2017). Pressure pulses at Voyager 2: drivers of interstellar transients?. Astrophys. J..

[CR220] Richardson J.D., Belcher J.W., Garcia-Galindo P., Burlaga L.F. (2019). Voyager 2 plasma observations of the heliopause and interstellar medium. Nat. Astron..

[CR221] Richardson J., Burlaga L., Elliott H., Kurth W., Liu Y., von Steiger R. (2022). Observations of the outer heliosphere, heliosheath, and interstellar medium. Space Sci. Rev..

[CR222] Rouillard A.P., Lockwood M. (2007). The latitudinal effect of corotating interaction regions on galactic cosmic rays. Sol. Phys..

[CR223] Roussos E. (2011). Long- and short-term variability of Saturn’s ionic radiation belts. J. Geophys. Res. Space Phys..

[CR224] Roussos E. (2019). Jovian cosmic-ray protons in the heliosphere: constraints by Cassini observations. Astrophys. J..

[CR225] Roussos E. (2020). Long- and short-term variability of galactic cosmic-ray radial intensity gradients between 1 and 9.5 au: observations by Cassini, BESS, BESS-Polar, PAMELA, and AMS-02. Astrophys. J..

[CR226] Scherer K., Fichtner H., Strauss R.D., Ferreira S.E.S., Potgieter M.S., Fahr H.J. (2011). On cosmic ray modulation beyond the heliopause: where is the modulation boundary?. Astrophys. J..

[CR227] Schwadron N.A., Adams F.C., Christian E.R., Desiati P., Frisch P., Funsten H.O., Jokipii J.R., McComas D.J., Moebius E., Zank G.P. (2014). Global anisotropies in TeV cosmic rays related to the Sun’s local galactic environment from IBEX. Science.

[CR228] Simpson J.A., Anglin J.D., Balogh A., Bercovitch M., Bouman J.M., Budzinski E.E., Burrows J.R., Carvell R., Connell J.J., Ducros R., Ferrando P., Firth J., Garcia-Munoz M., Henrion J., Hynds R.J., Iwers B., Jacquet R., Kunow H., Lentz G., Marsden R.G., Mckibben R.B., Meuller-Mellin R., Page D.E., Perkins M., Raviart A., Sanderson T.R., Sierks H., Treguer L., Tuzzolino A.J., Wenzel K.P., Wibberenz G. (1992). The ULYSSES cosmic ray and solar particle investigation. Astron. Astrophys. Suppl. Ser..

[CR229] Simpson J.A., Connell J.J., Lopate C., McKibben R.B., Zhang M. (1995). The latitude gradients of galactic cosmic ray and anomalous helium fluxes measured on Ulysses from the Sun’s south polar region to the equator. Geophys. Res. Lett..

[CR230] Stone E.C., Cummings A.C., McDonald F.B., Heikkila B.C., Lal N., Webber W.R. (2005). Voyager 1 explores the termination shock region and the heliosheath beyond. Science.

[CR231] Stone E.C., Cummings A.C., McDonald F.B., Heikkila B.C., Lal N., Webber W.R. (2008). An asymmetric solar wind termination shock. Nature.

[CR232] Stone E.C., Cummings A.C., McDonald F.B., Heikkila B.C., Lal N., Webber W.R. (2013). Voyager 1 observes low-energy galactic cosmic rays in a region depleted of heliospheric ions. Science.

[CR233] Stone E.C., Cummings A.C., Heikkila B.C., Lal N. (2019). Cosmic ray measurements from Voyager 2 as it crossed into interstellar space. Nat. Astron..

[CR234] Strauss R.D., Potgieter M.S. (2014). Is the highest cosmic-ray flux yet to come?. Sol. Phys..

[CR235] Strauss R.D., Potgieter M.S. (2014). Where does the heliospheric modulation of galactic cosmic rays start?. Adv. Space Res..

[CR236] Strauss R.D., Potgieter M.S., Kopp A., Büsching I. (2011). On the propagation times and energy losses of cosmic rays in the heliosphere. J. Geophys. Res. Space Phys..

[CR237] Strauss R.D., Potgieter M.S., Ferreira S.E.S. (2013). Modelling and observing Jovian electron propagation times in the inner heliosphere. Adv. Space Res..

[CR238] Strauss R.D., Potgieter M.S., Ferreira S.E.S., Fichtner H., Scherer K. (2013). Cosmic ray modulation beyond the heliopause: a hybrid modeling approach. Astrophys. J. Lett..

[CR239] Strong A.W., Moskalenko I.V., Ptuskin V.S. (2007). Cosmic-ray propagation and interactions in the galaxy. Annu. Rev. Nucl. Part. Sci..

[CR240] Sun X., Hoeksema J.T., Liu Y., Zhao J. (2015). On polar magnetic field reversal and surface flux transport during solar cycle 24. Astrophys. J..

[CR241] Thomas S.R., Owens M.J., Lockwood M., Scott C.J. (2014). Galactic cosmic ray modulation near the heliospheric current sheet. Sol. Phys..

[CR242] Tomassetti N. (2017). Testing universality of cosmic-ray acceleration with proton/helium data from AMS and Voyager-1. Adv. Space Res..

[CR243] Tomassetti N., Orcinha M., Barão F., Bertucci B. (2017). Evidence for a time lag in solar modulation of galactic cosmic rays. Astrophys. J. Lett..

[CR244] Tomassetti N., Barão F., Bertucci B., Fiandrini E., Orcinha M. (2019). Numerical modeling of cosmic-ray transport in the heliosphere and interpretation of the proton-to-helium ratio in solar cycle 24. Adv. Space Res..

[CR245] Vogt A., Heber B., Kopp A., Potgieter M.S., Strauss R.D. (2018). Jovian electrons in the inner heliosphere. Proposing a new source spectrum based on 30 years of measurements. Astron. Astrophys..

[CR246] Vos E.E., Potgieter M.S. (2016). Global gradients for cosmic-ray protons in the heliosphere during the solar minimum of cycle 23/24. Sol. Phys..

[CR247] Washimi H., Tanaka T., Zank G.P. (2017). Time-varying heliospheric distance to the heliopause. Astrophys. J. Lett..

[CR248] W.R. Webber, Observations of the abundances of secondary galactic cosmic rays from Z equals 5 to 28 between 10 and 200 MeV/nuc beyond the heliopause by Voyager, some unexpected anomalies and their interpretation using a LBM for galactic propagation. ArXiv e-prints (2016). 1612.08973

[CR249] Webber W.R., Kish J., Rockstroh J.M. (1973). Measurements of the primary cosmic ray electron spectrum from 1965 to 1972. International Cosmic Ray Conference.

[CR250] Webber W.R., Cummings A.C., McDonald F.B., Stone E.C., Heikkila B., Lal N. (2007). Passage of a large interplanetary shock from the inner heliosphere to the heliospheric termination shock and beyond: its effects on cosmic rays at Voyagers 1 and 2. Geophys. Res. Lett..

[CR251] Webber W.R., Cummings A.C., McDonald F.B., Stone E.C., Heikkila B., Lal N. (2009). Transient intensity changes of cosmic rays beyond the heliospheric termination shock as observed at Voyager 1. J. Geophys. Res. Space Phys..

[CR252] W.R. Webber, P.R. Higbie, F.B. McDonald, The unfolding of the spectra of low energy galactic cosmic ray H and He nuclei as the Voyager 1 spacecraft exits the region of heliospheric modulation. ArXiv e-prints (2013). 1308.1895

[CR253] Wiedenbeck M.E. (2013). Cosmic-ray energy spectra and time variations in the local interstellar medium: constraints and uncertainties. Space Sci. Rev..

[CR254] Zank G.P., Heerikhuisen J., Pogorelov N.V., Burrows R., McComas D. (2010). Microstructure of the heliospheric termination shock: implications for energetic neutral atom observations. Astrophys. J..

[CR255] Zank G.P., Adhikari L., Hunana P., Shiota D., Bruno R., Telloni D. (2017). Theory and transport of nearly incompressible magnetohydrodynamic turbulence. Astrophys. J..

[CR256] Zank G.P., Nakanotani M., Webb G.M. (2019). Compressible and incompressible magnetic turbulence observed in the very local interstellar medium by Voyager 1. Astrophys. J..

[CR257] Zhang M., Pogorelov N. (2020). Modulation of galactic cosmic rays by plasma disturbances propagating through the local interstellar medium in the outer heliosheath. Astrophys. J..

[CR258] Zhang M., Zuo P., Pogorelov N. (2014). Heliospheric influence on the anisotropy of TeV cosmic rays. Astrophys. J..

[CR259] Zhang M., Luo X., Pogorelov N. (2015). Where is the cosmic-ray modulation boundary of the heliosphere?. Phys. Plasmas.

[CR260] Zhang M., Pogorelov N.V., Zhang Y., Hu H.B., Schlickeiser R. (2020). The original anisotropy of TeV cosmic rays in the local interstellar medium. Astrophys. J..

[CR261] Zirnstein E.J., Heerikhuisen J., Funsten H.O., Livadiotis G., McComas D.J., Pogorelov N.V. (2016). Local interstellar magnetic field determined from the Interstellar Boundary Explorer ribbon. Astrophys. J. Lett..

[CR262] Zirnstein E.J., Heerikhuisen J., McComas D.J., Pogorelov N.V., Reisenfeld D.B., Szalay J.R. (2018). Simulation of the solar wind dynamic pressure increase in 2014 and its effect on energetic neutral atom fluxes from the heliosphere. Astrophys. J..

[CR263] Zirnstein E.J., Giacalone J., Kumar R., McComas D.J., Dayeh M.A., Heerikhuisen J. (2020). Turbulence in the local interstellar medium and the IBEX ribbon. Astrophys. J..

[CR264] Zucker C., Goodman A.A., Alves J., Bialy S., Foley M., Speagle J.S., Großschedl J., Finkbeiner D.P., Burkert A., Khimey D., Swiggum C. (2022). Star formation near the Sun is driven by expansion of the Local Bubble. Nature.

